# Genome-wide analysis of tandem repeats in *Daphnia pulex *- a comparative approach

**DOI:** 10.1186/1471-2164-11-277

**Published:** 2010-04-30

**Authors:** Christoph Mayer, Florian Leese, Ralph Tollrian

**Affiliations:** 1 Department of Animal Ecology, Evolution and Biodiversity, Ruhr University Bochum, Universitaetsstrasse 150, 44801 Bochum, Germany

## Abstract

**Background:**

DNA tandem repeats (TRs) are not just popular molecular markers, but are also important genomic elements from an evolutionary and functional perspective. For various genomes, the densities of short TR types were shown to differ strongly among different taxa and genomic regions. In this study we analysed the TR characteristics in the genomes of *Daphnia pulex *and 11 other eukaryotic species. Characteristics of TRs in different genomic regions and among different strands are compared in details for *D. pulex *and the two model insects *Apis mellifera *and *Drosophila melanogaster*.

**Results:**

Profound differences in TR characteristics were found among all 12 genomes compared in this study. In *D. pulex*, the genomic density of TRs was low compared to the arthropod species *D. melanogaster *and *A. mellifera*. For these three species, very few common features in repeat type usage, density distribution, and length characteristics were observed in the genomes and in different genomic regions. In introns and coding regions an unexpectedly high strandedness was observed for several repeat motifs. In *D. pulex*, the density of TRs was highest in introns, a rare feature in animals. In coding regions, the density of TRs with unit sizes 7-50 bp were more than three times as high as for 1-6 bp repeats.

**Conclusions:**

TRs in the genome of *D. pulex *show several notable features, which distinguish it from the other genomes. Altogether, the highly non-random distribution of TRs among genomes, genomic regions and even among different DNA-stands raises many questions concerning their functional and evolutionary importance. The high density of TRs with a unit size longer than 6 bp found in non-coding and coding regions underpins the importance to include longer TR units in comparative analyses.

## Background

The planktonic microcrustacean *Daphnia pulex *is a key species in lake ecosystems and forms an important link between the primary producers and the carnivores. It is among the best-studied animals in ecological, toxicological, and evolutionary research [1-4]. With the availability of the v1.1 draft genome sequence assembly for *D. pulex *it is now possible to analyse the genome in a comparative context.

Tandem repeats (TRs) are characteristic features of eukaryotic and prokaryotic genomes [5-13]. Traditionally, they are categorized according to their unit size into microsatellites (short tandem repeats, STRs, 1-6 bp (1-10 in some publications) repeat unit size), minisatellites (10 to approximately 100 bp repeat unit size), and longer satellite DNA (repeat units of >100 bp). Typically, STRs contribute between 0.5 - 3% to the total genome size.

TR loci in general, and micro- and minisatellite loci in particular, are often highly dynamic genomic regions with a high rate of length-altering mutations [14,15]. Therefore, they are frequently used as informative molecular markers in population genetic, forensic, and molecular ecological studies [6,16-22]. Due to their high abundance in genomes, microsatellites (STRs) are useful markers for genome mapping studies [23-26].

In contrast to the early view that TRs are mostly non-functional "junk DNA", the picture has emerged in recent years that a high proportion of TRs could have either functional or evolutionary significance [27-34]: TRs frequently occur within or in the proximity of genes, i.e., either in the untranslated regions (UTRs) up- and downstream of open reading frames, within introns, or in coding regions (CDS) [32]. Recent evidence supports that TRs in introns, UTRs, and CDS regions can play a significant role in regulating gene expression and modulating gene function [32,35,36]. Highly variable TR loci were shown to be important for rapid phenotypic differentiations [37,38]. They can act as "evolutionary tuning knobs" which allow fast genetic adaptations on ecological timescales [[34] for review, see also [39]]. Furthermore, TRs can be of profound structural as well as evolutionary importance, since genomic regions with a high density of TRs, e.g., telomeric, centromeric, and heterochromatic regions, often have specific properties such as alternative DNA structure and packaging. The structure of DNA can, in turn, influence the level of gene expression in these genomic regions [28,33,34,37,40]. Altogether, the analysis of the TR content of genomes is important for an understanding of genome evolution and organisation as well as gene expression and function.

### TR characteristics in different taxa and different genomic regions

With the rapid accumulation of whole genome sequence data in the last decade, several studies revealed that STR densities, usage of repeat types, length characteristics, and typical imperfection vary fundamentally between taxonomic groups [9,11,41-44] and even among closely related species [45-48]. In addition, strong differences of STR characteristics among different genomic regions have been described [9,12,43,44,49]. The often taxon-specific accumulated occurrence of certain repeat types in different genomic regions can hint at a functional importance of these elements. These characteristics are interesting from a comparative genomics as well as an evolutionary genomics point of view [9,11,12,43,44,50,51].

### Related work

Several studies have been conducted in the past to compare the characteristics of microsatellites (1-6 bp or 1-10 bp) among different taxa and different genomic regions, e.g. [9,44]. In these studies, however, the characteristics of TRs with a unit size >6 bp or >10 bp have been neglected. It has sometimes been argued that repeats with a unit size above 10 bp are generally rare in genomes, a presumption that has never been systematically tested. Furthermore, most studies are restricted to perfect TRs, with the main advantage that this significantly simplifies their identification. Disadvantages of this approach are that imperfections are a taxon-dependent natural feature of TRs and therefore should be included rather than neglected in an analysis. But even more important, TRs with long units tend to be more imperfect [10,52] so that a meaningful survey, which includes repeats with a unit size above 10 bp, has to include imperfect repeats.

Studies on characteristics of microsatellites can also be categorized according to whether they use the TR coverage of a sequence (in this paper referred to as the density, see Methods), or a number count of TRs per sequence length as the main characteristics of TRs. We recommend the use of a TR density (as in [9]) instead of number counts, since the latter do not represent the true TR content of a sequence. For example, the number count of a single perfect, 10000 bp long repeat, which might cover 20% of a sequence, is the same as that of a 20 bp repeat that only covers 0.04% of the same sequence. Depending on the number of mismatches, indels or sequencing errors, as well as the allowed degree of imperfection, the same 10000 bp repeat can be counted as one or a variety of different numbers of satellites. Hence, TR densities have the clear advantage that they show a much smaller dependence on the allowed degree of imperfection of a satellite than number counts.

### Aim

The aim of this comparative genomic study is to analyse the density and length characteristic of perfect and imperfect TRs in the 197.3 Mbp nuclear genome of the newly sequenced model crustacean *D. pulex *http://daphnia.cgb.indiana.edu/ and compare these to the characteristics of TRs in eleven other eukaryotic genomes from very different taxonomic groups ranging in size from 12.1 Mbp to 3080 Mbp (Table 1). For the annotated genomes of *Daphnia pulex*, *Drosophila melanogaster*, and *Apis mellifera *we also compare the repeat characteristics among different genomic regions (5'UTR, 3'UTR, CDS, introns, intergenic regions). In regions with a defined strandedness we also investigate whether the densities of repeat types differ from the densities of their reverse complements.

**Table 1:** List of species genomes analysed in the present study together with basic information on the genome assembly.

Species	Genome size [Mb]	CG content	Evolutionary domain	genome assembly version	source
*Daphnia pulex*	197.3	40.8%	animal/arthropoda	Dappu v1.1 (dpulex_jgi060905)	http://genome.jgi-psf.org/Dappu1/Dappu1.home.html
*Drosophila melanogaster*	168.7	41.7%	animal/arthropoda	dmel_r5.5_FB2008_01	ftp://ftp.flybase.net/genomes
*Apis mellifera*	228.6	32.8%	animal/arthropoda	Amel_2.0	ftp://ftp.hgsc.bcm.tmc.edu/pub/data/Amellifera/fasta/Amel20050120-freeze
*Apis mellifera*	235.2	32.7%	animal/arthropoda	Amel_4.0	ftp://ftp.hgsc.bcm.tmc.edu/pub/data/Amellifera/fasta/Amel20060310-freeze
*Caenorhabditis elegans*	100.3	35.4%	animal/nematoda	CaeEle-WS160	ftp://ftp.wormbase.org/pub/wormbase/
*Homo sapiens*	3080.4	40.9%	animal/vertebrata	build 36.2	ftp://ftp.ncbi.nih.gov/genomes/
*Mus musculus*	2644.1	41.8%	animal/vertebrata	build 36.1	ftp://ftp.ncbi.nih.gov/genomes/
*Gallus gallus*	1031.9	41.3%	animal/vertebrata	build 2.1	ftp://ftp.ncbi.nih.gov/genomes/
*Arabidobsis thaliana*	119.2	36.0%	plant/magnoliophytha	build 6.0	ftp://ftp.ncbi.nih.gov/genomes/
*Thalassiosira pseudonana*	31.3	46.9%	stramopiles/bacillariophytha	v3.031306	http://genome.jgi-psf.org/Thaps3/
*Ostreococcus lucimarinus*	13.2	60.4%	viridiplantae/chlorophyta	v2.0	http://genome.jgi-psf.org/Ost9901_3/
*Neurospora crassa*	39.2	49.3%	fungi/ascomycota	release 7	http://www.broadinstitute.org/annotation/genome/neurospora/MultiHome.html
*Saccharomyces cerevisiae*	12.1	38.3%	fungi/ascomycota	build 2.1	ftp://ftp.ncbi.nih.gov/genomes/

For *A. mellifera *the TR analysis of the complete genome was performed with assembly version 4.0, whereas analysis of the genomic regions was performed with assembly version 2.0 since the annotated features have not yet been officially mapped to the newer assembly.

## Methods

### Genome sequence data

The twelve sequenced genomes analysed in the present study are listed in Table 1. This list also contains the size, the CG-content, the assembly versions, and the download reference of the studied genomes. The size refers to the number of base pairs in the haploid genome. It reflects the current state of the genome build and includes known nucleotides as well as unknown nucleotides (Ns). CG-content, and genome size were determined with a self-written program. For *D. melanogaster*, the analysis of TRs in the complete genome includes the Het (heterochromatic), U and Uextra sequence files. Similarly, for *A. mellifera*, we included scaffolds in the file GroupUn_20060310.fa.

### Gene locations and features

For the *D. pulex *genome we obtained the most recent 'frozen gene catalogue' of the v1.1 draft genome sequence assembly from January 29th 2008 in the generic GFF (General Feature Format) from Andrea Aerts (DOE Joint Genome Institute), which in similar form is available from http://genome.jgi-psf.org/Dappu1/Dappu1.home.html. This catalogue contains the predicted and to some extent still putative gene locations. For each gene model, it provides the predicted locations of exons, and for most genes also the locations of coding regions, start and stop codons. Since the catalogue often contains multiple or alternative gene models at the same locus as well as duplicate or overlapping features of the same type within the same gene model, a C++ program was written by CM to remove multiple gene models in order to avoid an overrepresentation of these loci in the analysis. To be more precise, if two predicted gene models overlapped and if both genes were found in the same reading direction, the longer of the two gene models was removed. Similarly, if two exons or two coding (CDS) features of the same gene overlapped, the longer of the two features was removed. Introns and intergenic regions were identified by the locations of exons that are associated to the same gene model. If available, the start and stop codon positions within exons of a gene were used to infer the locations of 5' and 3'UTR. This information on the positions of different genomic regions was finally used to split the genome sequences into six sequence files, each containing the sequence fragments associated to exons, introns, 5'UTRs, 3'UTRs, CDS, or intergenic regions. Since the TR characteristics of exons are just a combination of the TR characteristics of CDS and UTR regions, they have not been included in the present analysis.

For *A. mellifera *we used the same procedure as for *D. pulex*. A GFF file with annotation information was obtained from http://genomes.arc.georgetown.edu/Amel_abinitio_on_assembly2.gff. Unfortunately, the annotated features have so far not been officially mapped on assembly version 4.0, so the TR analysis of genomic regions had to be performed with assembly version 2.0.

For the *D. melanogaster *genome, separate sequence files for the six different features of interest can readily be downloaded from ftp://ftp.flybase.net/genomes. Since also these files contain multiply or alternatively annotated features, again a C++ program written by CM was used to consistently remove the longer of two overlapping features if both were of the same feature type and annotated in the same reading direction. The separate sequence files for different genomic regions do not include the sequence fragments found in the Het (heterochromatic), U and Uext sequence files of the current assembly, since these regions have not yet been annotated [53].

For the 5'UTRs, 3'UTRs, introns, and CDS regions of the three genomes we extracted and analysed always the sense strand of the corresponding gene. This provides the opportunity to identify differences in the repeat characteristics of the sense and anti-sense strands, i.e. to search for a so-called strandedness.

### Terms and Conventions

For a given TR unit, the associated *repeat type *is defined as follows: All TRs with units that differ from the given repeat unit only by circular permutations and/or the reverse complement are associated to the same repeat type. Clearly, there are always several repeat units, which belong to the same repeat type. We follow the convention to represent a repeat type by that unit which comes first in an alphabetical ordering of all units that are associated to it [54]. This convention allows us to count and identify repeat units without reference to the repeat unit phase or strand. To give an example, the repeat type represented by the unit AAG incorporates all TRs with units AAG, AGA, GAA, TTC, TCT, and CTT. Furthermore, the term *repeat motif *is used instead of the term *repeat type *when we aim at distinguishing between sense and anti-sense strand repeat characteristics, but not the repeat phase. Hence, on the level of repeat motifs, AAG, AGA, GAA are all represented by AAG, but are distinguished from the repeat motif CTT, which also represents TTC and TCT. Finally, the terms *repeat type *and *repeat motif *are distinguished from the term *repeat class *which we use to denote the collection of all repeats with the same repeat unit size (e.g. mono-, di-, trinucleotide repeats).

An important property of one or a set of TR types is their *density *within a nucleotide sequence. It is defined as the fraction of base pairs that are found within repeats of a given set of repeat types over the total number of base pairs in the sequence. Repeat type densities are measured in base pairs per megabase pairs (bp/Mbp). It can be envisaged as the coverage of the sequence with the specified repeat types. Since in several genomes, including *D. pulex*, the number of (Ns) contributes significantly to the total size, all TR densities computed in this work were corrected for the number of Ns. It is important to distinguish repeat densities from densities based on number counts of repeats (measured in counts/Mbp) that are sometimes used in publications, e.g. [44,47,51].

### TR detection and analysis

The characteristics of perfect and imperfect TRs strongly depend on the properties individual satellites have to fulfil to be included in the analysis. For perfect TRs this is the minimum repeat length or its associated alignment score, which in TR search programs is often defined as a function of the unit size. Changing the minimum unit size has an effect not only on the total density of different TR types, but also on relative densities since the length distribution of different repeat types usually differ strongly. For imperfect TRs it is additionally necessary to restrict or penalize their imperfection, e.g. with a mismatch and gap penalty. Furthermore, an optimality criterion has to be specified that determines which of two alternative alignments of a putative TR locus with its perfect counterparts is to be preferred.

In the present work, TRs were detected using Phobos, version 3.2.6 and 3.3.0 [55]. Phobos is a highly accurate TR search tool that is able to identify perfect and imperfect TRs in a unit size range from 1 bp to >5000 bp without using a pre-specified motif library. The optimality criterion Phobos uses is the alignment score of the repeat region with a perfect repeat counterpart. This means that each putative TR is extended in both directions as far as possible, by including gaps and mismatches, if this leads to a higher alignment score (see the Phobos manual for details [55]). For the present analyses, the alignment scores for match, mismatch, gap and N positions were 1, -5, -5, 0 respectively. In every TR the first repeat unit was not scored. Only a maximum number of four successive Ns were allowed. For a TR to be considered in the analysis it was required to have a minimum repeat alignment score of 12 if its unit size was less or equal to 12 bp or a score of at least the unit size for unit sizes above 12 bp. As a consequence, mono-, di-, and trinucleotide repeats were required to have a minimum length of at least 13, 14, and 15 bp to achieve the minimum score. For repeat units above 12 bp a perfect repeat had to be at least two units long, an imperfect repeat even longer, to achieve the minimum score. For this study, imperfect TRs were analysed in two size ranges: 1-50 bp and 1-4000 bp. For both size ranges a recursion depth of five was used. For the size range 1-50 bp the *maximum score reduction *was unlimited, for the size range 1-4000 bp the *maximum score reduction *was set to 30 to accelerate the computation while preserving a good accuracy. For details concerning the search strategy of Phobos and its scoring scheme the reader is referred to the Phobos manual [55].

Phobos has been used for this analysis since it is more accurate in the unit size range 1-50 bp than other TR search tools. Besides searching for imperfect repeats, Phobos is also able to identify whether alternative alignments exist for a TR. For example, the (ACACAT)_N _repeat can be viewed as an imperfect dinucleotide or a perfect hexanucleotide repeat. In this discipline, the Tandem Repeats Finder (TRF) [52] is the only alternative. While it is the state of the art in the detection of imperfect repeats with long unit sizes, it is based on a probabilistic search algorithm. In particular, it is less accurate when detecting TRs with a short unit size and a small number of copies. In contrast, Phobos uses an exact (non-probabilistic) search algorithm necessary for a meaningful statistical analysis of TR characteristics. The search parameters used in this analysis are being compared to the default search parameters used in the TRF program in the Additional file 1. TR characteristics such as the density and mean length of repeat types were computed using the program *Sat-Stat*, version 1.3.1 developed by CM.

In principle, results can be compared to TR databases available [56-60]. However, due to the differences in search parameters and problems related to probabilistic searches such a comparison makes sense in few cases only and has therefore not been performed in this study.

## Results

### Characteristics of STRs in all 12 genomes

#### Genomic density

**Figure 1:**
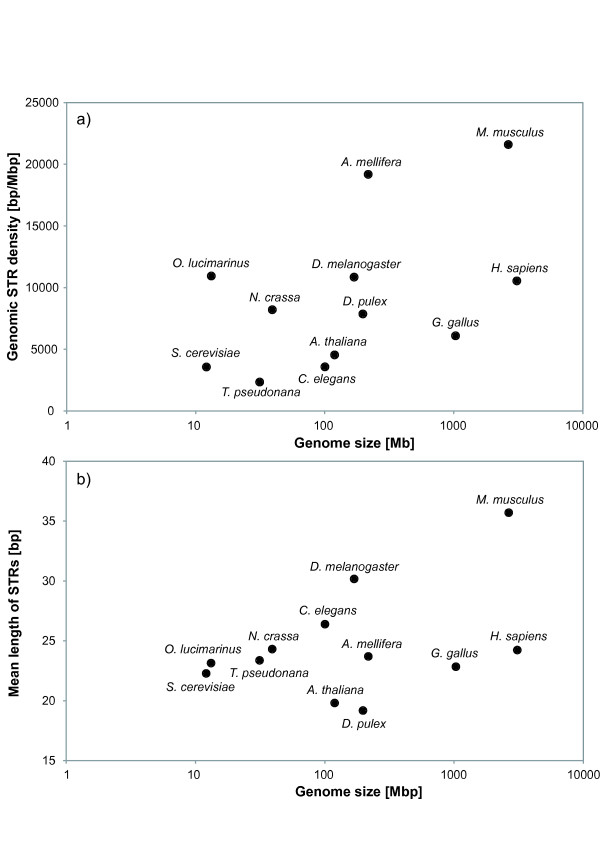
**a) Genome size (on logarithmic scale) versus genomic TR density and b) mean repeat lengths of perfect and imperfect short tandem repeats (1-6 bp) in *Daphnia pulex *and 11 other eukaryotic genomes**. In the Additional file 2 we provide four related Figures where the information found in Figure 1 is shown separately for perfect and purely imperfect tandem repeats.

For a first comparison the genomic density of imperfect STRs has been plotted against the genome size of the twelve species analysed in this study (Figure 1a). The genome sizes as well as the genomic densities of STRs vary considerably among the 12 taxa. The three arthropods in this analysis, *D. pulex, D. melanogaster*, and *A. mellifera*, show only slight differences in genome size, but large differences in the density of STRs (Figure 1a, Table 2). Among the three arthropods, *D. pulex *has by far the lowest STR density with a value of almost one third of *A. mellifera*. Compared to all other 11 genomes the STR density in *D. pulex *is about average. No significant correlation was found between the genome size and the density of STRs (Pearson correlation coefficient: R = 0.483, P = 0.111). See also Additional file 2, where the data of Figure 1 are presented for perfect and for truly imperfect TRs in two separate graphs. Most notable, *D. pulex*, but also *A. mellifera *have much higher densities of perfect than imperfect STRs.

**Table 2:** Main characteristics of STRs in the genome of *Daphnia pulex *and 11 other taxa.

	Mono	Di	Tri	Tetra	Penta	Hexa	total
*D. pulex*							
total counts	17161	16104	21458	4630	3476	2382	65211
density [bp/Mbp]	1747.1	1884.8	2662.0	632.9	534.8	418.0	7866.0
density [%]	22.2	23.9	33.8	8.0	6.8	5.3	100.0
mean length	16.2	18.6	19.7	21.7	24.5	27.8	19.2
standard deviation	4.1	5.5	6.4	11.2	20.2	22.9	

*D. melanogaster*							
total counts	15701	14511	9622	4770	5984	8220	58808
density [bp/Mbp]	1639.0	1961.6	1543.7	939.2	3253.7	1538.1	10876.0
density [%]	15.1	18.0	14.2	8.6	29.9	14.1	100.0
Mean length	17.0	22.0	26.1	32.1	89.3	30.6	30.2
standard deviation	10.1	13.3	31.0	65.3	189.5	52.8	

*A. mellifera*							
total counts	54104	69036	28005	16755	11528	8673	188101.0
density [bp/Mbp]	4166.4	7860.8	3181.9	1742.1	1313.4	1008.6	19184.1
density [%]	21.6	40.8	16.5	9.0	6.8	5.2	100.0
mean length	17.8	26.3	26.3	24.1	26.5	27.0	23.7
standard deviation	6.0	17.1	24.1	18.0	29.6	55.0	

*C. elegans*							
total counts	4104	3004	2570	927	582	2430	13617
density [bp/Mbp]	653.4	727.3	480.6	224.9	120.2	1373.0	3577.7
density [%]	18.3	20.3	13.4	6.3	3.4	38.4	100.0
mean length	16.0	24.3	18.8	24.3	20.7	56.8	26.4
standard deviation	3.8	23.5	5.4	32.1	6.9	72.3	

*H. sapiens*							
total counts	591617	224459	77621	200797	105107	55324	1254925
density [bp/Mbp]	3889.6	2267.4	658.7	2249.0	1032.1	511.5	10547.3
density [%]	36.7	21.4	6.2	21.2	9.7	4.8	100.0
Mean length	18.8	28.9	24.3	32.2	28.3	26.5	24.2
standard deviation	5.7	20.7	28.9	53.4	84.7	24.7	

*M. musculus*							
total counts	286206	563483	127365	351575	140683	93653	1562965
density [bp/Mbp]	2107.5	8913.8	1877.2	5360.1	1994.9	1517.1	21601.6
density [%]	9.7	40.9	8.6	24.6	9.2	7.0	100.0
Mean length	18.8	40.4	37.7	39.2	36.6	41.6	35.7
standard deviation	7.4	31.7	45.3	40.4	36.4	49.3	

*G. gallus*							
total counts	142803	27674	21667	39190	24240	9382	264956
density [bp/Mbp]	2617.8	625.8	450.1	1138.0	964.8	330.2	6094.5
density [%]	42.7	10.2	7.3	18.6	15.7	5.4	100.0
Mean length	18.1	22.3	20.5	28.8	39.5	35.0	22.8
standard deviation	6.0	11.2	7.8	34.1	46.3	41.6	

*A. thaliana*							
total counts	10492	6471	6017	982	1540	1840	27342
density [bp/Mbp]	1468.7	1264.9	1052.8	158.9	253.7	350.0	4544.2
density [%]	32.3	27.8	23.1	3.5	5.6	7.7	100.0
Mean length	16.7	23.3	20.8	19.3	19.6	22.7	19.8
standard deviation	4.2	13.9	14.1	9.1	3.7	7.8	

*T. pseudonana*							
total counts	29	344	2222	237	101	202	3135
density [bp/Mbp]	13.0	235.8	1457.0	188.0	66.4	385.3	2344.3
density [%]	0.6	10.1	62.1	8.0	2.8	16.4	100.0
mean length	14.0	21.4	20.5	24.7	20.5	59.5	23.4
standard deviation	1.5	9.3	16.5	12.1	3.3	123.2	

*O. lucimarinus*							
total counts	5	2051	2208	285	610	1124	6283
density [bp/Mbp]	5.0	3075.5	3980.7	553.7	1160.2	2223.8	10941.6
density [%]	0.0	28.0	36.2	5.0	10.5	20.2	100.0
Mean length	13.2	19.8	23.8	25.7	25.2	26.2	23.1
standard deviation	0.5	8.2	11.0	9.6	9.3	9.9	

*N. crassa*							
total counts	3216	915	4484	1788	1000	1889	13292
density [bp/Mbp]	1971.0	535.5	2752.7	1095.2	619.3	1250.6	8207.7
density [%]	24.0	6.5	33.5	13.3	7.5	15.2	100.0
Mean length	24.0	23.0	24.1	24.0	24.4	26.1	24.3
standard deviation	11.7	14.4	17.6	14.4	10.7	14.7	

*S. cerevisiae*							
total counts	810	335	414	67	71	256	1953
density [bp/Mbp]	1157.7	646.7	900.4	118.6	133.4	639.1	3563.5
density [%]	32.2	18.0	25.0	3.3	3.7	17.8	100.0
mean length	17.3	23.3	26.5	21.4	22.7	30.2	22.3
standard deviation	5.5	10.8	15.4	8.0	5.4	27.7	

#### Mean length

A comparison of genome sizes and mean lengths of imperfect STRs of all 12 genomes is shown in Figure 1b. Even though the mean repeat length depends crucially on the search parameters for TRs, general trends can be seen in this comparison: STRs are shortest in *D. pulex *(average length 19.48 bp) and longest in *M. musculus *(average length 38.3 bp), see Figure 1b and Table 2. No significant correlation between genome size and mean length of STRs was found (Pearson correlation coefficient: R = 0.489, P = 0.107).

Whereas for the three vertebrate species a high TR density is correlated with a high value of the mean repeat length, no similar correlation can be observed for the three arthropods. While *A. mellifera *has a STR density of almost twice the value of *D. melanogaster*, the STRs are on average 20% longer in *D. melanogaster *than in *A. mellifera*. In the Additional file 2, we present separate analyses of perfect and truly imperfect TRs. Most notable is that *C. elegans*, despite of its low density of truly imperfect repeats has on average very long imperfect TRs.

#### Genomic densities of mono- to hexanucleotide repeat classes

**Figure 2:**
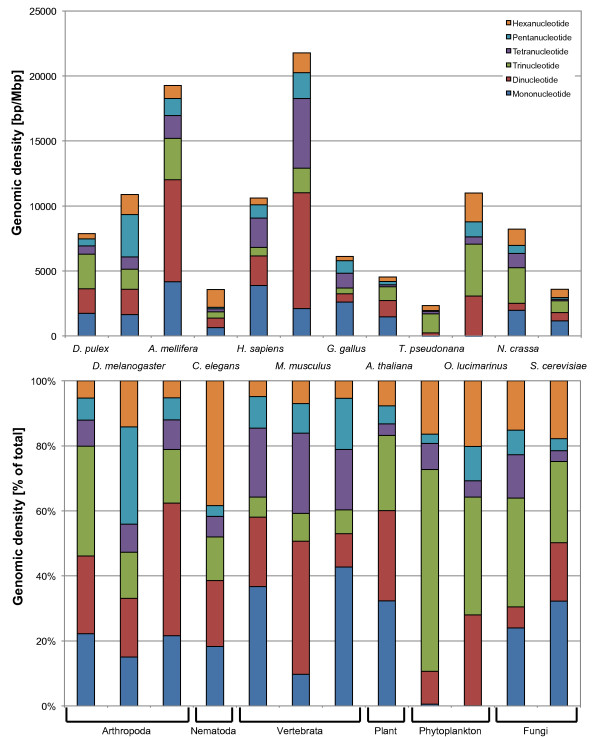
**Absolute genomic densities (*upper panel*) and relative genomic densities (*lower panel*) of short tandem repeats (mono- to hexanucleotide repeats) in *Daphnia pulex *and 11 other genomes**.

A more detailed comparison of the genomic densities of mono- to hexanucleotide repeat classes of all 12 taxa is presented in Figure 2. Whereas the upper panel shows the absolute repeat class densities, the lower panel shows their relative contribution to the STR density. Even better than from Figure 1a it becomes obvious that the absolute STR densities are highly variable even among taxonomically more closely related taxa such as the three arthropod species, the vertebrates, or the fungi species. Comparing the relative densities of STR classes, some taxon-specific trends are detectable (Figure 2, lower panel): *C. elegans *has a high relative density of hexanucleotide repeats, whereas pentanucleotide repeats are rare. All vertebrate species exhibit a particularly high proportion of tetranucleotide repeats while trinucleotide repeats are relatively rare. The two phytoplankton species have almost no mononucleotide repeats longer than 12 bp (minimum score 12, see Methods), whereas trinucleotide repeats are highly overrepresented. A high proportion of trinucleotide repeats is also found in the two fungi.

Comparing the relative densities of STR classes among the three arthropod species, we find that trinucleotide repeats are strongly overrepresented in *D. pulex*, contributing 30% to all STRs (Figure 2). The proportions of mono-, tetra-, penta-, and hexanucleotide repeats are almost identical in *D. pulex *and *A. mellifera*. With the exception of similar tetranucleotide densities there are no common features among *D. pulex *and the other two arthropod species.

#### Genomic densities of mono- to trinucleotide repeat types

Repeat type usage of mono-, di-, and trinucleotide repeats in the 12 genomes is very different (Table 3). Only the density of ACT repeats is consistently low in all species. Even among more closely related species, only few common features can be observed. Poly-A repeat densities are generally high except for *T. pseudonana *and *O. lucimarinus*, where they are even lower than poly-C repeats. In *D. pulex*, poly-C repeats have the highest genomic density compared to the other genomes. In vertebrates, AAT repeat densities are similarly high, CCG repeat densities are low, and ACG repeats are virtually absent. Among the three arthropods, only the relatively low densities of the ATC repeats are of similar magnitude. The repeat types AC, ACG, and CCG with low densities for most taxa have particularly high densities in *O. lucimarinus*. The AGG repeat type has high densities only in *A. mellifera *and *M. musculus*.

**Table 3:** Tandem repeat types of mono- to trinucleotide repeats for the genome of *D. pulex *and eleven other taxa.

Repeat type	*D. pulex*	*D. melanogaster*	*A. mellifera*	*C. elegans*	*H. sapiens*	*M. musculus*	*G. gallus*	*A. thaliana*	*T. pseudonana*	*O. lucimarinus*	*N. crassa*	*S. Cerevisiae*	min	max
A	1199.3	1473.4	4090.9	410.4	3877.7	1934.1	2474.2	1446.3	2.3	1.0	1632.4	1152.5	1.0	4090.9
C	547.8	165.6	75.5	243.0	11.9	173.4	143.6	22.4	10.7	4.0	338.6	5.2	4.0	547.8

AC	804.9	1008.4	359.9	208.7	1288.1	5353.6	204.4	82.7	48.6	11.8	229.7	79.0	11.8	5353.6
AG	955.1	309.1	3168.7	284.2	405.2	2662.5	105.3	426.8	181.7	7.7	217.3	23.4	7.7	3168.7
AT	119.1	644.2	4289.6	232.7	575.4	897.4	316.2	755.5	5.6	0.0	87.7	544.7	0.0	4289.6
CG	5.8	0.1	44.8	1.8	3.1	17.7	0.3	0.1	0.0	3056.1	1.2	0.0	0.0	3056.1

AAC	422.1	211.3	93.7	26.5	166.6	324.3	148.0	128.6	584.2	10.0	544.2	182.5	10.0	584.2
AAG	916.3	27.3	714.8	149.0	73.9	525.1	28.4	515.0	90.0	87.6	332.6	171.7	27.3	916.3
AAT	212.7	426.7	1214.4	90.6	229.8	241.7	169.7	81.1	1.0	0.0	178.6	251.4	0.0	1214.4
ACC	138.8	61.1	94.2	47.8	36.0	134.5	13.4	39.5	121.1	6.9	390.6	9.8	6.9	390.6
ACG	150.5	29.0	166.8	20.0	0.3	3.0	0.5	9.5	127.9	3030.8	158.7	24.2	0.3	3030.8
ACT	77.1	25.7	103.6	14.8	6.8	32.2	4.3	15.1	36.6	1.3	55.4	17.9	1.3	103.6
AGC	495.0	536.9	47.7	36.7	21.8	69.8	28.1	19.8	193.8	12.1	385.3	101.8	12.1	536.9
AGG	73.5	62.7	599.6	19.0	63.8	486.5	33.9	60.7	136.4	58.6	367.0	16.7	16.7	599.6
ATC	89.2	116.5	100.6	65.1	41.8	53.7	7.8	179.2	153.6	8.4	200.8	126.8	7.8	200.8
CCG	88.3	47.8	50.3	11.2	18.7	13.4	16.2	4.8	14.8	767.1	143.7	0.0	0.0	767.1

### Characteristics of TRs with unit sizes 1-50 bp in all 12 genomes

**Figure 3:**
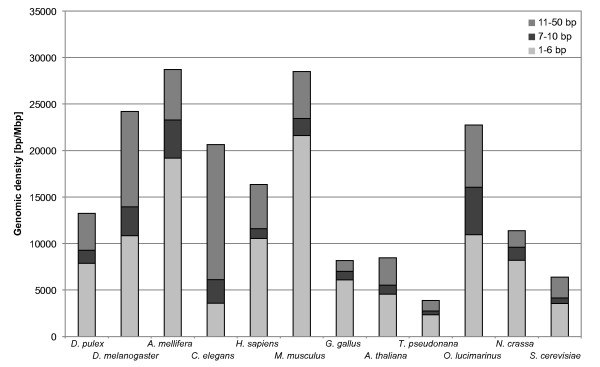
**Genomic density of tandem repeats in the three different unit size ranges 1-6 bp, 7-10 bp and 11-50 bp for *Daphnia pulex *and 11 other genomes**.

In contrast to most studies that only analysed STRs with a unit size of 1-6 bp, we compared the TR content of the 12 genomes in three unit size ranges: 1-6 bp, 1-10 bp, and 1-50 bp (Figure 3). The results show that in all 12 genomes the density of TRs with a unit size in the range 7-50 bp contributes significantly to the density of TRs in the unit size range 1-50 bp. The contribution ranges between 26.1% in *M. musculus *and 83.5% in *C. elegans *with a mean value of 42.8%. The contribution of 40.9% in *D. pulex *is slightly below average. In three genomes, i.e., *D. melanogaster*, *C. elegans*, and *O. lucimarinus*, the density of TRs with a unit size above 6 bp exceeds the density of STRs (Figure 3).

Among the 12 genomes, strong differences are found for the density of TRs in the three unit size ranges and in individual repeat classes (Additional file 3). No systematic pattern can be observed for the arthropod, vertebrate, or fungi genomes. Compared to the other 11 genomes, the TR density in *D. pulex *is slightly below average in all three unit size ranges. Among the three arthropods, *D. pulex *has not only the lowest density of STRs as mentioned before, but also a density of TRs in the unit size range 1-50 bp which is about half the value found for *D. melanogaster *and *A. mellifera *(Figure 3, Table 4). For the three arthropod species in this study a more detailed analysis of the genomic density and length characteristics of TR classes in the range 1-50 bp is given in the following two sections.

**Table 4:** Repeat characteristics of TR classes with a unit size of 1 to 50 bp for *Daphnia pulex, Drosophila melanogaster*, and *Apis mellifera*.

	*Daphnia pulex*					*Drosophila melanogaster*					*Apis mellifera*				
	
repeat class [bp]	total counts	mean TR length [bp]	sd	longest TR [bp]	density [bp/Mbp]	total counts	mean TR length [bp]	sd	longest TR [bp]	density [bp/Mbp]	total counts	mean TR length [bp]	sd	longest TR [bp]	density [bp/Mbp]
1	17161	16.2	4.1	100	1747.1	15701	17.0	10.1	974	1639.0	54104	17.8	6.0	335	4166.4
2	16104	18.6	5.5	117	1884.8	14511	22.0	13.3	820	1961.6	69036	26.3	17.1	630	7860.8
3	21458	19.7	6.4	174	2662.0	9622	26.1	31.0	710	1543.7	28005	26.3	24.1	932	3181.9
4	4630	21.7	11.2	336	632.9	4770	32.1	65.3	863	939.2	16755	24.1	18.0	943	1742.1
5	3476	24.5	20.2	379	534.8	5984	89.3	189.5	9308	3253.7	11528	26.5	29.6	1348	1313.4
6	2382	27.8	22.9	536	418.0	8220	30.6	52.8	1969	1538.1	8673	27.0	55.0	4290	1008.6
7	1664	23.5	8.9	239	246.4	5133	41.3	73.0	973	1291.0	7810	27.1	16.7	593	914.7
8	1591	24.5	17.0	582	245.3	3204	43.4	89.7	1540	846.5	8585	27.0	15.4	774	1003.3
9	2213	27.4	10.0	191	380.1	2386	39.6	93.9	3013	577.7	8557	28.5	16.8	496	1054.9
10	2041	45.2	89.2	1459	572.3	1535	50.2	86.6	929	473.8	9572	32.7	62.9	2548	1351.8
11	1336	27.8	8.2	161	234.0	2776	369.6	953.0	33316	6269.0	7884	29.3	14.0	592	997.5
12	1814	35.7	41.9	906	404.9	2310	161.4	628.0	23884	2272.4	8313	31.2	20.1	1023	1120.6
13	786	35.3	20.3	234	174.7	530	34.6	34.7	567	113.0	4001	34.4	22.2	595	596.0
14	459	37.9	19.2	247	109.7	419	57.1	59.9	589	147.3	2383	37.4	19.7	385	385.0
15	780	50.9	35.3	491	249.8	415	63.5	70.7	607	161.1	1421	48.8	69.0	1084	299.4
16	177	68.9	96.9	915	76.9	179	56.5	51.6	538	62.2	852	49.2	47.3	785	181.2
17	389	270.2	386.6	3259	650.4	92	60.1	78.2	632	34.1	558	50.6	50.2	850	121.9
18	366	85.9	158.0	2317	196.1	212	68.0	100.7	1311	88.5	509	67.1	89.4	1124	147.1
19	284	59.4	45.9	605	106.1	110	63.5	76.3	538	43.0	309	58.1	43.3	673	77.7
20	155	85.2	81.6	663	83.2	89	98.7	127.4	684	54.1	279	90.7	204.1	2150	109.5
21	363	70.9	33.4	401	162.3	89	204.7	577.3	5404	111.7	221	78.7	71.8	587	75.2
22	91	72.3	47.9	298	40.7	45	443.2	1638.9	11089	122.8	150	72.8	69.5	576	47.3
23	77	97.7	136.1	1192	47.3	175	553.9	838.9	6227	553.7	118	108.7	155.0	1134	55.5
24	350	152.9	139.0	931	337.0	130	110.7	117.4	699	88.4	169	127.7	254.0	2621	93.4
25	43	83.4	74.0	437	22.6	23	248.4	219.9	747	31.1	84	131.0	275.9	2531	47.6
26	62	95.7	74.7	499	37.4	45	125.0	164.5	842	34.7	214	226.1	212.0	1673	200.9
27	128	107.9	69.1	475	87.0	85	93.9	59.1	416	46.7	98	138.6	134.3	700	58.8
28	42	84.6	32.1	231	22.4	46	134.1	129.5	551	38.0	106	167.0	191.0	1261	75.7
29	29	85.1	37.1	201	15.6	14	163.7	183.3	680	14.1	64	144.4	146.0	1052	40.0
30	119	152.5	161.9	1141	110.5	89	146.9	134.2	1085	80.5	136	150.7	194.4	1995	87.5
31	95	94.6	36.0	261	56.6	55	581.4	503.9	2320	181.7	44	167.0	154.4	879	31.8
32	70	93.1	51.7	330	39.9	56	196.7	174.1	744	67.9	56	148.3	133.6	769	35.9
33	85	152.2	159.2	1349	80.9	112	457.9	2864.8	30381	315.8	69	146.4	93.7	487	43.6
34	61	210.2	256.8	1552	80.8	51	444.4	607.3	2944	139.6	77	161.8	125.8	862	53.9
35	94	140.0	106.1	675	82.9	25	849.3	1816.1	9031	130.8	81	164.5	121.1	760	57.2
36	81	155.4	94.6	447	79.3	61	231.1	129.7	569	85.3	360	183.8	94.3	805	285.9
37	25	156.7	163.8	880	24.7	21	150.3	70.2	347	19.4	60	173.1	112.1	557	45.0
38	55	112.7	29.1	260	39.1	20	189.8	162.6	638	23.4	47	137.5	74.6	428	28.0
39	39	153.7	115.3	674	37.8	69	361.5	1153.3	9681	153.5	54	162.5	133.9	816	38.0
40	14	105.4	38.1	234	9.3	10	322.4	277.9	972	19.9	47	231.0	306.6	1984	47.0
41	31	105.8	21.6	158	20.7	3	247.7	230.3	513	4.6	38	160.3	108.1	683	26.3
42	70	139.9	54.9	428	61.6	26	189.9	205.4	980	30.4	32	186.1	158.6	862	25.8
43	38	130.9	35.7	203	31.4	10	364.4	339.9	1045	22.5	17	236.7	218.7	969	17.4
44	10	183.0	95.8	344	11.5	4	915.3	1379.3	2979	22.6	34	200.8	205.5	1288	29.6
45	56	208.9	241.5	1442	73.8	32	409.6	630.3	3489	80.7	41	243.3	288.7	1875	43.2
46	11	363.9	701.5	2475	25.2	12	2057.9	3322.3	11248	152.1	40	180.0	96.8	524	31.2
47	12	313.5	339.9	1263	23.7	12	619.6	611.0	1987	36.0	20	233.1	152.4	561	20.2
48	39	335.3	225.0	857	82.4	12	1126.3	2226.0	7791	83.3	19	253.2	148.5	615	20.8
49	15	218.5	155.7	620	20.7	19	174.5	151.6	700	20.4	32	227.0	204.5	926	30.6
50	37	172.1	60.3	350	40.1	10	245.5	129.0	469	15.1	40	252.0	219.2	1200	43.6

total	81508	26.2		3259	13254	79559	53.6		33316	24199	251702	27.0		4290	28698

#### Densities of the 1-50 bp repeat classes in the three arthropod species

**Figure 4:**
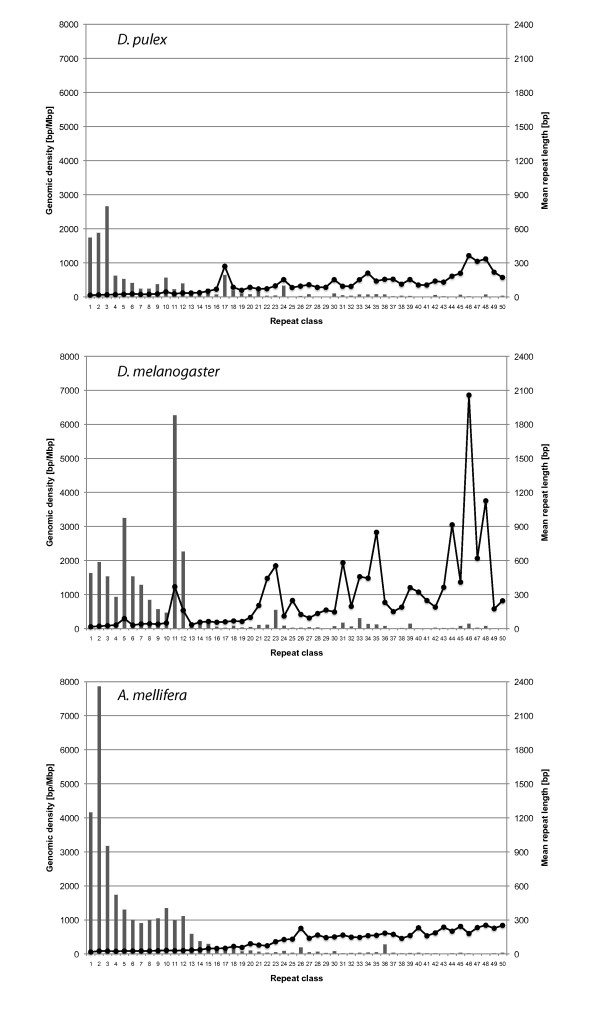
**Genomic density of tandem repeats with a unit size of 1-50 bp (dark columns) and their respective length characteristics (grey lines with boxes) for the three arthropod species investigated in this study**.

Densities of the TR classes in the range 1-50 bp show strong differences among the three arthropod species (Figure 4, Table 4). In *D. pulex*, trinucleotide repeats represent the dominant repeat class followed by di- and mononucleotide repeats. Together, these three repeat classes contribute 47.16% to the total density of all repeat classes from 1-50 bp. Other repeat classes with a local maximum in the repeat class density are the 10, 12, 17, and 24 bp repeats (Table 4, Additional file 4). *D. melanogaster*, in contrast to the other two arthropods, shows a strong heterogeneity in repeat class densities. Genomic density is highest for TRs with a unit size of 11 bp followed by peaks at 5 and 12 bp (Table 4, Figure 4). Relatively high density peaks are also found for the repeat classes 21-24 bp, 30-36 bp, 39, 43, 45, and 46 bp. Especially for the longer repeat classes, there are usually only very few repeat types which contribute to the density of their repeat classes. For instance, the individual repeat types ACCAGTACGGG, ACCGAGTACGGG, and ACCAGTACGGGACCGAGTACGGG contribute 95.2% (5967.1 bp/Mbp), 76.4% (1736.4 bp/Mbp), and 71.0% (393.3 bp/Mbp) to the density of the (dominating) repeat classes 11 bp, 12 bp, and 23 bp, respectively. All three repeat types are highly similar, which shows that ACCAGTACGGG is the dominating repeat type in this genome. In *A. mellifera*, as in *D. pulex*, STR classes contribute most to the overall TR density. Mono- to tetranucleotide repeat densities are higher than in the two other arthropods. The highest density is contributed by the dinucleotide repeats, which have a genomic density more than three times as high as in the other two arthropod species. The small local density maxima at 10 and 12 bp are similar to *D. pulex*. TRs with longer repeat units have very low densities with a small local maximum only for 26 bp and 36 bp repeats.

#### Mean lengths of the 1-50 bp repeat classes in the three arthropod species

Similar to the repeat densities, strong differences between the mean lengths of TRs with respect to the unit size are observed for the three arthropod species (Figure 4, Table 4). Since the minimum length of TRs is twice the unit size, it is expected to see a trend toward longer repeats for an increasing unit size. Roughly, this trend can be confirmed for *D. pulex *and *A. mellifera*, whereas for *D. melanogaster *a trend can only be seen when not taking into account some of the repeat classes with extraordinarily long repeats. In *D. pulex *and *A. mellifera*, all mean repeat lengths are shorter than 254 bp in the unit size range 1-50 bp. *D. pulex *shows a notable peak for the mean repeat lengths of 17 bp repeats, a repeat class that is discussed in detail below. Among the smaller peaks in the mean repeat length spectrum of *D. pulex *there is a trend towards peaks that correspond to repeat classes that are multiples of three base pairs (Figure 4, Additional file 4).

In contrast, *D. melanogaster *has mean repeat length peaks above 500 bp for several repeat classes. This explains why the genomic density of TRs found in *D. melanogaster *is twice as high as in *D. pulex *even though the total number of TRs is lower (Table 4). A maximum mean repeat length of 2057 bp is found for the 46 bp repeat class which consists of 12 repeats ranging in length from 355 bp to 11248. It should be mentioned at this point that the high densities of longer repeat classes in *D. melanogaster *are concentrated in the heterochromatic regions of this genome. The sequencing and assembly of these regions was so difficult that this was done in a separate *Heterochromatin Genome Project *[61,62]. See also the discussion below.

### Characteristics of TRs with unit sizes 1-50 bp in different genomic regions

Patterns of TR densities and length characteristics were analysed in detail for the different genomic regions of *D. pulex*, its reference genome *D. melanogaster*, and *A. mellifera *(Figures 5, 6, 7, Additional file 5). The number of sequences in the genomic regions, their base content and length characteristics are given in Table 5. Both median and mean sizes of the different genomic regions are listed for a more comprehensive picture. The same information, but for the repeat sequences is given in Table 6. Comparing the TR densities among corresponding genomic regions in the unit size ranges 1-6 bp, 1-10 bp and 1-50 bp (Figure 5), the TR densities were generally highest in *A. mellifera*, lower in *D. melanogaster *and lowest *D. pulex*, with the only exception of a higher TR density in introns of *D. pulex *than in *D. melanogaster*. In all three genomes, the density contribution of the 7-50 bp repeat classes to all repeats in the size range 1-50 bp is much higher in CDS and intergenic regions than in introns and UTRs (see also Additional file 5). In CDS regions the contribution of 7-50 bp repeats is highest, with 72.8% in *D. pulex*, followed by 52.1% and 44.0% in *D. melanogaster *and *A. mellifera*, respectively. For all three species and in all size ranges, the densities are lowest in CDS regions. TR densities in *D. pulex *and *A. mellifera *are highest in introns in all unit size ranges, followed by intergenic regions, with a much higher difference in *D. pulex*. In *D. melanogaster*, STRs are most abundant in 3'UTRs closely followed by introns, 5'UTRs, and intergenic regions (Additional file 5). In the unit size range 1-50 bp, repeats are more dense in intergenic regions due to the high density of TRs with longer units in the vicinity of heterochromatic regions. It should be noted that a major proportion of heterochromatic regions is not included in the intergenic regions data set (see Methods for the origin of these files), since in these regions genes are not reliably annotated. However, since there are no clear boundaries between heterochromatic and euchromatic regions, some of the typical repeats found in heterochromatic regions are also found in the intergenic regions.

**Table 5:** Characteristics of the CDS, introns, and intergenic regions of *D. pulex*, *D. melanogaster*, and *A. mellifera*.

	*Daphnia pulex*			*Drosophila melanogaster*			*Apis mellifera*		
	**CDS**	**introns**	**Intergenic**	**CDS**	**introns**	**intergenic**	**CDS**	**introns**	**intergenic**

#sequences	120611	114156	36090	14817	47897	12371	31734	18117	23205
Min length	1	1	0	78	11	1	1	49	0
Max length	12685	48487	168617	69048	166135	345402	7242	34351	773536
Median length	147.0	76.0	1294.0	1152.0	75.0	1006.0	54.0	578.0	2986.0
Mean length	212.8	284.1	3659.1	1537.0	1080.0	5371.1	80.7	909.4	9007.8
SD lengths	279.0	1050.9	7082.0	1704.2	4197.7	13770.7	120.0	1245.9	22243.2
# positions	25665454	32436000	132056549	22774339	51727142	66445483	2561048	16475821	209026309
A	0.284	0.290	0.301	0.257	0.295	0.296	0.334	0.340	0.336
C	0.240	0.190	0.199	0.268	0.202	0.203	0.175	0.152	0.165
G	0.232	0.190	0.198	0.266	0.197	0.203	0.207	0.158	0.165
T	0.245	0.331	0.302	0.210	0.306	0.298	0.285	0.350	0.335
CG	0.472	0.380	0.397	0.534	0.399	0.406	0.381	0.310	0.329
corrected CpG	0.976	1.016	1.003	0.880	0.916	0.939	1.172	1.694	1.662
corrected CpNpG	1.088	1.082	1.063	1.086	1.068	1.099	1.005	0.962	0.959
A/T	1.157	0.875	0.999	1.225	0.967	0.995	1.174	0.970	1.000
C/G	1.034	0.997	1.002	1.005	1.023	1.001	0.844	0.963	1.000

**Table 6:** Characteristics of the TRs found in the CDS regions, introns, and intergenic regions of *D. pulex*, *D. melanogaster*, and *A. mellifera*.

	*Daphnia pulex*			*Drosophila melanogaster*			*Apis mellifera*		
	**CDS**	**introns**	**intergenic**	**CDS**	**introns**	**intergenic**	**CDS**	**introns**	**intergenic**

#sequences	4175	22763	53058	4340	30418	32625	863	18130	222929
Min length	13	13	13	13	13	13	13	13	13
Max length	2460	682	3303	9420	3049	33848	1151	943	3005
Median length	23.0	18.0	19.0	24.0	20.0	21.0	27.0	22.0	22.0
Mean length	45.9	20.8	27.3	38.5	24.5	44.4	57.5	26.5	26.3
SD lengths	86.5	14.7	54.7	161.7	44.4	400.1	92.8	25.0	30.2
# positions	191505	472915	1446267	167031	746049	1447496	49605	480452	5863495
A	0.300	0.271	0.306	0.333	0.335	0.282	0.363	0.396	0.403
C	0.295	0.188	0.195	0.306	0.172	0.217	0.164	0.083	0.098
G	0.254	0.182	0.193	0.263	0.159	0.219	0.230	0.084	0.098
T	0.151	0.359	0.307	0.099	0.334	0.281	0.243	0.438	0.401
CG	0.549	0.370	0.387	0.568	0.331	0.437	0.394	0.166	0.196
corrected CpG	0.762	0.434	0.612	0.409	0.622	1.137	0.820	1.113	1.191
corrected CpNpG	1.187	0.860	0.942	1.695	1.381	1.709	0.856	0.551	0.545
A/T	1.991	0.754	0.996	3.383	1.005	1.002	1.496	0.905	1.004
C/G	1.160	1.035	1.012	1.162	1.081	0.991	0.713	0.983	0.996

**Figure 5:**
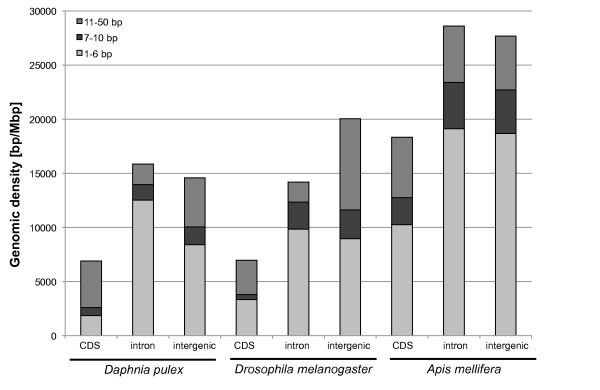
**Tandem repeat densities in different genomic regions of *Daphnia pulex, Apis mellifera, and *the euchromatic genome of *Drosophila melanogaster *in the unit size ranges 1-6 bp, 7-10, and 11-50 bp**.

#### TR classes

*Genomic densities *of TR classes show high dissimilarities among the different genomic regions of *D. pulex*, *D. melanogaster, and A. mellifera*. In CDS regions of all three genomes, repeat densities are dominated by repeat classes with unit sizes that are multiples of 3 bp, consistent with the reading frame (Additional file 5, Figure 6), see also [63]. Notable exceptions are 10 and 20 bp repeat classes in *D. pulex *and 10 bp, 11 bp, and 16 bp repeat classes in *A. mellifera*, which have not only relatively high densities in CDS regions, but also relatively long repeat regions. The proportion of repeats (based on number counts) in the unit size range 1-50 bp not consistent with the reading frame is 11.4% in *D. pulex*, 3.1% in *D. melanogaster*, and 22.7% in *A. mellifera*.

**Figure 6:**
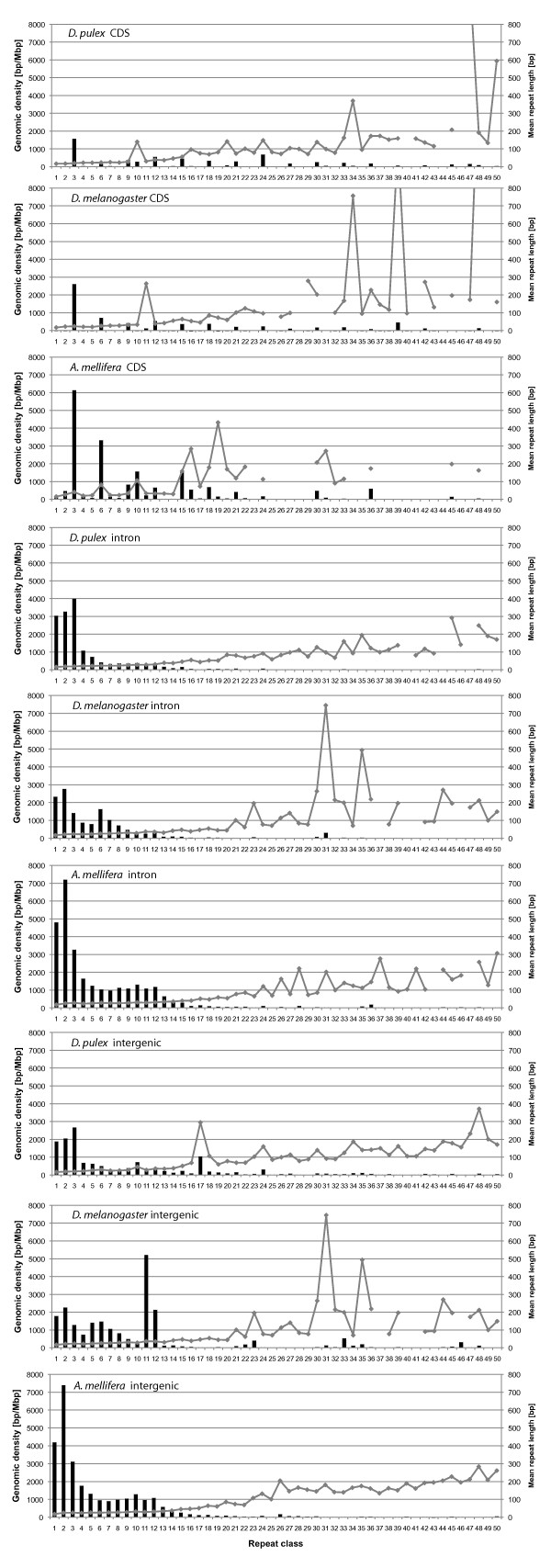
**Genomic density of tandem repeats with a unit size of 1-50 bp in different genomic regions in *Daphnia pulex*, the euchromatic genome of *Drosophila melanogaster, and Apis mellifera *(columns) and their respective average lengths (grey lines, secondary y-axis)**.

Several repeat classes are more dense in CDS regions than in other regions, e.g. the densities of the 24 bp repeat class in *D. pulex*, the 39 bp repeat class of *D. melanogaster*, and the 6, 10, 15, 16, 18, 21, 30, 36 bp repeat classes of *A. mellifera *are significantly higher in CDS regions than in all other regions. In a separate analysis conducted only for *D. pulex*, we searched for TRs in the size range 1-4000 bp in CDS regions. The results show repeat densities above 100 bp/Mbp also for the 51, 52, 60, 75, 108, and the 276 bp repeat classes. A list of all TRs found in CDS regions of *D. pulex *is given in Additional file 6.

In introns of *D. pulex *and *D. melanogaster *the proportion of STRs is higher than in the other genomic regions, whereas in *A. mellifera*, with a general trend to shorter repeat units, this cannot be observed. In *D. pulex*, the repeat classes with a unit size of 1-5 bp and 7-8 bp show by far the highest densities in introns as compared to other genomic regions (Additional file 5). Most dominant are trinucleotide repeats, which are more dense in introns of *D. pulex *than in introns of *D. melanogaster *and *A. mellifera*. A notable feature in introns of *D. melanogaster *is the relatively high density of the 31 bp repeat class. The intergenic regions of *D. pulex *and *D. melanogaster *show high densities for several longer repeat classes which are rare or absent in other regions (Figure 6, Additional file 5). In *D. pulex*, e.g., the 17 bp repeat class shows a high repeat density only in intergenic regions, whereas in the other two arthropods it is relatively rare in all genomic regions. Repeat classes with a particularly high density in intergenic regions can be found in Additional file 5. Concerning the UTRs in *D. pulex*, the TR statistics has to be treated with caution for repeat classes longer than 3 bp, since only a small proportion of genes has well annotated UTRs so that the total number of TRs found in 5' and 3'UTRs (135 and 653) is low. For example, the inflated density of the 24 bp repeat class in 5'UTRs of *D. pulex *is based on just a single 272 bp long repeat. As a general result, TRs with short units dominate in UTRs.

*Mean lengths *of the TR classes in the different genomic regions are more heterogeneous in *D. melanogaster *than in *D. pulex *and *A. mellifera*. This is not just the case for intergenic regions including the heterochromatin, but also in introns (e.g. the 31 bp repeat class) and CDS regions (e.g. 39 bp and 48 bp repeat classes), see Figure 6.

#### TR motifs and strandedness

**Figure 7:**
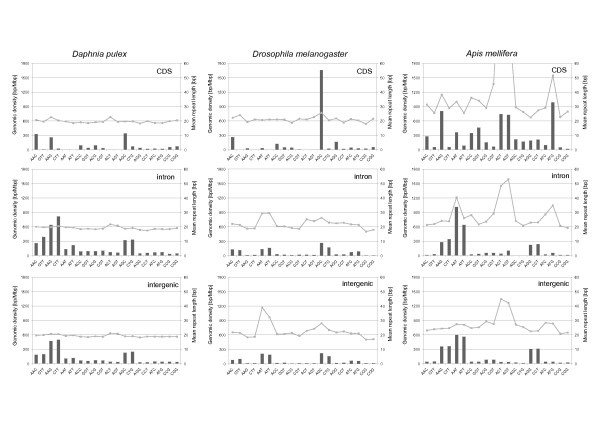
**Genomic density of trinucleotide repeat motif pairs (normal and reverse complement) in different genomic regions of *Daphnia pulex, Drosophila melanogaster, and Apis mellifera***. Whereas in intergenic regions both types are always of similar density, in introns and CDS regions there are often strong differences in densities supporting a strand-specific repeat motif usage (strandedness). Lines with boxes show the respective mean repeat length (secondary y-axis).

For genomic regions with annotated sense and anti-sense strands, we analysed whether the characteristics of TRs with certain repeat units differ on the two strands. In order to investigate this question we (i) always analysed the sense strand of annotated gene features and (ii) reported the repeat unit in a form normalized only with respect to the repeat phase (cyclic permutations), here called the repeat motif, instead of the repeat type, normalized with respect to phase and strand (cyclic permutations and the reverse complement, see Methods for details). Results, which include the information on the repeat motif strandedness are presented in Figure 7 and in the Additional file 7.

For *D. pulex, D. melanogaster*, and *A. mellifera *repeat motif usage shows only few common features among the genomes and different genomic regions. Common features of all three genomes are a relatively high density of poly-A/T repeats in introns and intergenic regions, low densities of CG repeats in all regions, and higher densities of AAC and AGC repeats in CDS regions than in introns and intergenic regions. Repeat motifs that are more dense in introns than in CDS and intergenic repeats of all three genomes are poly-T, AT and GT (Additional file 7). Several repeat motifs show a strong strandedness in the CDS regions of all three genomes. Most notable are the repeat motifs AAC and AAG, which have much higher densities than their reverse complements GTT and CTT. A smaller but still existing trend is observed for AAT versus ATT repeats. Strandedness also occurs in introns of *D. pulex*, where poly-T repeats have much have higher densities than poly-A repeats. Other motif pairs with considerably different densities on the sense strand in introns are ATT versus AAT, CT versus AG, GT versus AC, and ATTT versus AAAT. In all these examples T-rich motifs are preferred on the sense strand.

Restricting the search for common features to *D. pulex *and *D. melanogaster *one finds that CCG/CGG repeats are predominantly found in CDS regions, whereas AT repeats show their highest densities in 3'UTRs (data not available for *A. mellifera*), see Additional file 7. The absolute densities of the AT repeat type in 3'UTRs, however, differ significantly with values of 220.5 and 2663.6 bp/Mbp in *D. pulex *and *D. melanogaster*, respectively. In both genomes, the dominant repeat motif in CDS regions is AGC, with a particularly high density of 1658.9 bp/Mbp in CDS regions of *D. melanogaster*.

Curiously, for both genomes (*D. pulex *and *D. melanogaster*), the repeat motif AGC shows much higher densities on the sense strand of CDS regions than its reverse complement, the repeat motif CTG (340.7 bp/Mbp versus 74.7 bp/Mbp and 1658.9 bp/Mbp versus 26.9 bp/Mbp, see Additional file 7). In introns of *D. pulex*, a strandedness for this motif is not present, whereas in introns of *D. melanogaster *it is much less pronounced. In contrast to *D. pulex *and *D. melanogaster*, the repeat motif AGC has only a moderate density in all regions of *A. mellifera*. Conversely, the dominant repeat motif in CDS regions of *A. mellifera*, ATG, is very rare in the other two genomes. Also this repeat motif shows a considerable strandedness in CDS regions of *A. mellifera*. Other repeat motifs with a high density in CDS regions of *A. mellifera*, but with low densities in the other genomes are ACT and AGT. Also notable is the high density of the dinucleotide (and thus reading frame incompatible) repeat motif CT (435.8 bp/Mbp) in CDS regions of *A. mellifera *and the strong discrepancy to the low density of its reverse complement AG (20.3 bp/Mbp). As mentioned before, short units are dominant in introns of all three genomes. Dominant repeat motifs in introns of *D. pulex *are poly-T followed by CT and CTT. Among tetranucleotide repeats, the motifs CTTT and ATTT show the highest densities. All these motifs have higher densities than their reverse complements. In introns of *D. melanogaster*, dominant repeat motifs are poly-A followed by poly-T and AT, with only a small strandedness of poly-A versus poly-T repeats. Densities in introns of *A. mellifera *are high for several repeat motifs. Most notable are the motifs AT followed by poly-A, poly-T, CT, AG, and AAT. The density of AT repeats in introns of *A. mellifera *(4069.0 bp/Mbp) constitutes the highest repeat motif density among the three genomes and their genomic regions. A notable strandedness is observed for the poly-A versus poly-T and for AAT versus ATT repeat motifs. In CDS regions of *A. mellifera *a high strandedness is also found for the AAGCAG motif (1480 bp/Mbp) versus CTGCTT (0.00 bp/Mbp). In introns, the two motifs still have the respective densities of 46.3 bp/Mbp versus 0.00 bp/Mbp.

Concerning the mean perfection of TR motifs in different genomic regions (see table in Additional file 7, page 10 for details) we could not find many general trends. In different genomic regions of *D. pulex*, the mean imperfection in the size range 1-50 bp was 98.36% in CDS regions, 99.09% in intergenic regions, and 99.31% in introns (the mean values are not shown in above mentioned table). For *A. mellifera *we found on average lower repeat perfections of 97.35% in CDS regions, 98.57% in intergenic regions, and 98.52% in introns. For *D. melanogaster*, mean repeat perfections are 97.35% in CDS regions, 98.55% in intergenic regions and 98.68% in introns. So in all three genomes, the mean repeat perfection is lowest in CDS regions. Differences in repeat perfection among introns and intergenic regions are small.

Strong differences among the three genomes are found for several repeat motifs: poly-C and poly-G densities are particularly low in *A. mellifera*, AT repeat densities are 20 and 30 times higher in intergenic regions and introns of *A. mellifera *as compared to *D. pulex *and A_n_G (n = 1 to 5) and ACG densities are much higher in *D. pulex *and *A. mellifera *than in *D. melanogaster*. For instance AAG repeat densities are about 40 times higher in introns and intergenic regions of *D. pulex *than in the same regions of *D. melanogaster*. Potentially interesting are TRs in CDS regions where the unit size is not directly compatible with the reading frame. As mentioned above, 10-mer repeats (and multiples of 10) have significant densities in CDS regions of *D. pulex*. Most notable are the repeat types AACCTTGGCG (Dappu-343799, Dappu-344050, Dappu-343482, Dappu-279322, Dappu-280555), ACGCCAGAGC (Dappu-264024, Dappu-264706, Dappu-275708), and ACGCCAGTGC (Dappu-267284, Dappu-267285, Dappu-275706, Dappu-275708, Dappu-277192). These three repeat types are completely absent in *D. melanogaster *and *A. mellifera*. Repeat motif usage in UTRs was only compared if the number of satellites in these regions was sufficiently high. All TR characteristics including the number counts are listed in Additional file 7. As a general result, repeat type usage is very heterogeneous on a genomic level as well as among different genomic regions. Within a given TR class there are usually only a few TR motifs which contribute to the density of the repeat class (Figure 7, Additional file 7).

*Mean lengths *of mono- to trinucleotide repeat types in different genomic regions of *D. pulex *show a relatively homogeneous length distribution, in contrast to the heterogeneous densities (Figure 7, Additional file 5). Peaks in average repeat length in the UTRs (see Additional file 5 and 7) must be regarded with caution due to small samples sizes (see above). In *D. melanogaster *and *A. mellifera*, TRs are generally longer than in *D. pulex*.

### TRs with a unit size of 17 bp in *D. pulex*

The repeat class in *D. pulex *with the highest repeat density and a unit size longer than three base pairs is the 17 bp repeat class (Table 4). There are several notable aspects of these repeats: first of all, the true genomic density of 17 nucleotide repeats is likely to be underestimated in the current assembly since several scaffolds start or end with a 17-nucleotide repeat. For instance, the longest imperfect repeat found in *D. pulex *with a total length of 3259 bp is a 17 nucleotide repeat located at the end of scaffold 66. Three very similar repeat types, (AAAAGTTCAACTTTATG with 273.0 bp/Mbp, mean length 318.5 bp, AAAAGTAGAACTTTTCT with 209.8 bp/Mbp, mean length 739.62 bp, AAAAGTTCTACTTTGAC with 88.9 bp/Mbp, mean length 705.3 bp) contribute 88% to the total repeat density of 17 bp repeats. (Further repeat types were found that are similar to these three.) A striking characteristic of these repeat types is their high similarity to their reverse complement. The two repeat types with the highest density have only 5 non-matching positions when aligned to their reverse complement. This might hint at a functional role or structural importance of these repeats - see discussion. The mean length of all imperfect 17-mer nucleotide repeats is 270 bp, which is the highest value for repeats with a unit shorter than 46 bp in *D. pulex*. Repeats of the 17 bp repeat class are mostly found in intergenic regions with a density of 1039.4 bp/Mbp and mean length of 295.0 bp.

### TRs with unit sizes above 50 bp in *D. pulex*

**Figure 8:**
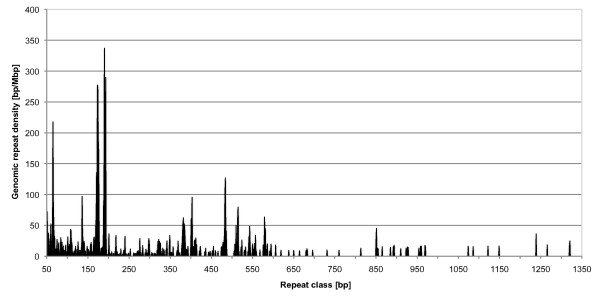
**Genomic densities of tandem repeat classes in the unit size range 50 - 4000 bp in the genome of *D. pulex***. The TR with the longest unit found in this genome has a unit size of 1121 bp. An accumulation of repeat densities is observed for specific repeat unit sizes, e.g. around 160 bp and 190 bp.

The results of the search for imperfect TRs in *D. pulex *with a motif size of 1-4000 bp are shown in Figure 8, in which the size range 1-50 bp has been removed since they are shown in Figure 4 and Additional file 4. The density spectrum shows an irregular pattern of density hotspots in certain size ranges. The TR with the longest unit size (1121 bp) has a total length of 2589 bp, which corresponds to 2.31 repeat units. TRs with a unit size of 171 bp are very abundant. They have the same size as the well-known alpha-satellites. Alpha-satellites are a family of long TRs near the centromers in vertebrate chromosomes and have frequently been reported [64]. Homology searches (Dotplots, BLAST) could not identify any similarity between the *D. pulex *satellites and the known alpha satellites of *M. musculus *and *H. sapiens*. Among the 10 non-mammalian genomes only *D. pulex *has a particularly high density of satellites in the unit size range 165-175 bp.

## Discussion

Tandem repeats, together with interspersed repeats, are key features of eukaryotic genomes and important for the understanding of genome evolution. For the newly sequenced crustacean *D. pulex *we have analysed the characteristics of TRs and compared them to the TR characteristics of 11 other genomes from very different evolutionary lineages. A particular focus was on comparing the genomes of *A. mellifera *and the model insect *D. melanogaster *because of their shared ancestry with *Daphnia *within the Pancrustacea, and despite their large evolutionary divergence, they best served to help annotate the *D. pulex *genome.

A general problem of TR analyses is that the detection criteria, the allowed degree of imperfection, the optimality criterion as well as the accuracy of the search algorithm can significantly influence the characteristics of TRs found in a search [65,66]. Therefore, a direct comparison of TR characteristics of different genomes is only possible if analyses were carried out by the same search tool using the same search parameters. Despite differences in the detection criteria, a comparison of TR type densities for *Homo sapiens *analysed in this study and by Subramanian et al. [12] agree well in terms of absolute and relative densities (see Table 3 in this paper and Figures 3, 4 and 5 in [12]) supporting that general trends can well be independent of the search criteria. While Subramanian et al. [12] also used TR densities as the main characteristics, many studies rely on number counts. This type of data is difficult to compare to analyses using TR densities. Hence, in this paper we have compared our results mainly with those in Tóth et al. [9], since their detection criteria (perfect STRs, minimum length 13 bp), main characteristics (TR densities) and the compared taxa still come closest to those used in the present analysis. All comparisons drawn here have been confirmed (in a separate analysis) to hold true also when using the same search parameters as in [9].

### Comparisons of TRs in the 12 genomes

Our analyses show that TRs contribute considerably to all genomes analysed in this study, which is consistent with earlier results ([5,9,11,12,51,67] and many others). No TR characteristics were found that are common to all of the 12 genomes, except for a relatively low density of ACT repeats, which has already been reported in Tóth et al. [9]. The dominance of taxon rather than group specific characteristics has also been reported in [44,51] when comparing number counts of satellites. As a general trend, Tóth and collaborators [9] also observed an underrepresentation of ACG repeats in most taxa. Our data support this trend with the striking exception of *O. lucimarinus*, where ACG repeats constitute the highest individual trinucleotide repeat type density in this study (Table 3). Curiously, the high absolute and relative di- and trinucleotide repeat densities found in *O. lucimarinus *are exclusively based on the high densities of the CG, ACG, and CCG repeat types that are uncommon in all other taxa in this study (see discussion below). The high CG-content of these three dominant repeat types is consistent with the high CG-content (60%) of the genome of *O. lucimarinus*.

Even within evolutionary lineages, common features of TR characteristics are rare. Notable are the clear dominance of poly-A over poly-C repeat types in all genomes except for the diatom and the green algae, the almost complete absence of mononucleotide repeats in the diatom and the green algae, and the almost complete absence of ACG repeats in vertebrates (Figure 2 and Table 3). Our data also supports the result of Tóth et al. [9] that the relative high proportion of tetranucleotide over trinucleotide repeat densities in vertebrates could not be found in any other taxonomic group. To establish these features as lineage specific, still more taxa need to be analysed. Besides these few cases of group specific similarities, this study reveals a high level of dissimilarity in genomic repeat class and repeat type densities among all taxonomic groups. Among the fungi, for example, the genomes of *N. crassa *and *S. cerevisiae *show no lineage specific similarities. In contrast to Tóth et al. [9], where AT and AAT repeats were the dominant di- and trinucleotide repeat types in genomes of fungi, *N. crassa *has a more than 2.6 times higher density of AC than AT repeats and a more than 3 times higher density of AAC than AAT repeats in this study. Also the three arthropod species, *D. pulex*, *D. melanogaster*, and *A. mellifera *show no remarkable similarities among mono- to hexanucleotide repeat class (Figure 2) or mono- to trinucleotide repeat type densities (Additional file 7). Several common features of arthropods that have been found in [9] cannot be confirmed in the present analysis: whereas these authors found dinucleotide TRs to constitute the dominant repeat class in arthropods, this cannot be confirmed in the present study for *D. pulex *where the density of trinucleotide repeats exceeds the density of dinucleotide repeats by 40%. Furthermore, in [9] AC was the dominant dinucleotide and AAC and AGC the dominant trinucleotide repeat types in arthropods, which is not the case for the genomes of *A. mellifera *and *D. pulex*. Most striking, the AC, AAC, and AGC repeat type densities are particularly low in *A. mellifera*, a genome for which an untypical repeat type usage, as compared to other arthropods, has already been mentioned in [68]. *A. mellifera *also stands out as the taxon with the highest density of mononucleotide repeats in this study, whereas in [9] this repeat class was found to be densest in primates. In contrast to [9], where penta- and hexanucleotide repeats were "invariably more frequent than tetranucleotide repeats in all non-vertebrate taxa", this cannot be confirmed in the present study.

Going beyond the scope of previous TR analyses ([9,11,43,44] and others), we compared characteristics of TRs with unit sizes in the range 1-50 bp. Our results reveal that imperfect TRs with unit sizes larger than 6 bp contribute significantly to the TR content of all genomes analysed. The model nematode *C. elegans*, e.g., was commonly thought to have a very low density of genomic TRs [9], which is true for the unit size range 1-5 bp, but not for the size range 6-50 bp (Additional file 2, see also Figure 3). This finding leads to a completely new picture for the TR content of this organism.

Concerning the mean lengths of STR, this study showed that the genome of *D. pulex *is characterized by shorter STRs than the other genomes. Furthermore, among the STRs, perfect repeats have a higher density than imperfect repeats. Neglecting the still unknown contribution of unequal crossing-over to length altering mutations of STRs, their equilibrium lengths are the result of slippage events extending STRs and point mutations breaking perfect TRs into shorter repeats [41,46,69,70]. The dominance of relatively short STRs in the genome of *D. pulex *indicates that the 'life cycle' of a typical TR is comparatively short, i.e. the frequency of interrupting point mutations is relatively high compared to extending slippage mutations. Furthermore, it has been discussed in the literature whether the typical length of TRs is inversely correlated to the effective population size (see e.g. [19]). Since large population sizes are a feature of *D. pulex*, our results are not in conflict to this conjecture.

Another interesting point is the typical perfection of TRs. Perfect TRs are believed to be subject to more length altering mutations than imperfect repeats, since a higher similarity of sequence segments increases the chance of slippage and homologous crossing-over events. Since the STRs found in *D. pulex *but also those in *A. mellifera *are predominantly perfect, we expect an increased number of length altering mutations in these two genomes. The mutability of STRs in *D. pulex *has been studied in detail by another group of the Daphnia Genomics Consortium, which compares the rate and spectrum of microsatellite mutations in *D. pulex *and *C. elegans *[71]. In view of this remark it is interesting that TRs in the size range 1-50 bp are on average more imperfect in CDS regions of all three arthropod genomes as compared to introns and intergenic regions.

A direct comparison of TRs with a unit size of 1-50 bp among the three arthropods shows remarkable differences. The dominant repeat classes (highest to lower densities) are the 2, 1, 3, 4, 5, and 10 bp repeat classes of *A. mellifera*, the 3, 2, 1, 17, 4, and 10 bp repeat classes in *D. pulex *and the 11, 5, 12, 2, 1, and 3 bp repeat classes in *D. melanogaster*. This highlights the trend towards shorter motifs in *A. mellifera *in contrast to the trend towards longer motifs in *D. melanogaster*. The relative dominance of 3 bp repeats in *D. pulex *likely reflects the great number of genes (>30000; Daphnia Genomics Consortium unpublished data) in this comparatively small genome. This same paper also states that *D. pulex *is one of the organisms most tightly packed with genes. Similar to the repeat densities, the mean lengths of TRs show remarkable differences among the three arthropods. An elevated mean length of TRs in a repeat class can hint at telomeric and centromeric repeats. In *D. pulex*, candidates for telomeric and centromeric repeats are found in the 17, 24, and 10 bp repeat classes. Since the long 17 bp repeats are usually located at the beginning or end of scaffolds, their true density is likely to be underestimated. Interestingly, just three very similar repeat types contribute 87% of the density to this repeat class. It is worth noting that the two repeat types with the highest density have only 5 non-matching positions when aligned to their reverse complement, which could lead to the formation of alternative secondary structures, see e.g. [33,72].

As mentioned above, the CG, ACG and CCG repeat types are rare in all taxa except for *O. lucimarinus*, where the densities of these repeats are particularly high. Usually, the low densities of these motifs are explained by the high mutability of methylated CpG dinucleotides (as well as CpNpG trinucleotides in plants, where N can be any nucleotide), which efficiently disrupts CpG rich domains on short timescales. Since CCG repeat densities are also low in several organisms that do not methylate (*C. elegans*, *Drosophila *and yeast), Tóth et al. [9] argue in favour of other mechanisms, which lead to low CCG repeat densities, particularly in introns. According to our data, CpG and CpNpG mutations must certainly be suppressed in TR regions of *O. lucimarinus*. Furthermore, mechanisms which act against CpG-rich repeats in other species are not in effect in this genome. The particularly high densities of CG, ACG, and CCG as compared to all other mono- to trinucleotide repeat types in *O. lucimarinus *even raises the question whether CpG-rich repeats are simply favoured for unknown reasons, or whether they are prone to particularly high growth rates if their occurrence is not suppressed.

Interesting in this respect is a direct comparison of the densities of the ACG and AGC repeat types, which have identical nucleotide content on the same strand, but which differ in the occurrence of the CpG dinucleotide. The density ratio of AGC to ACG repeats ranges from high values in the vertebrates with a value of 63.4 in *H. sapiens *to 0.0040 in *O. lucimarinus *(Table 3). Even among the three arthropod species, this density ratio differs considerably: *D. pulex *(3.3), *A. mellifera *(0.28), *and D. melanogaster *(18.5). Interestingly, *A. mellifera *and *O. lucimarinus *are the only two species for which the density of ACG repeats is higher than the density of AGC repeats. Among the three arthropods, *A. mellifera *has the highest content of CpG containing TRs despite its lowest value for the genomic CG-content (34.9%) in this study. Consistent with this observation, a CpG content higher than in other arthropods and higher than expected from mononucleotide frequencies has been found previously, even though *A. mellifera *methylates CpG dinucleotides [73].

In *D. pulex*, the densities of A_n_× (n = 1 to 10) repeat types are significantly overrepresented, a feature that has also been observed for other, distantly related species (*H. sapiens *[12], *A. thaliana *[44]). Lawson and Zhang [44] have argued that these repeats could have evolved from mutations in poly-A repeats.

### TRs in genomic regions and their potential function

Several recent studies have shown that TRs are not just "junk DNA" but play an important role in genome organization, gene regulation and alternating gene function. They have gained particular interest due to their potential for rapid adaptations and several authors regard them as hotspots for evolutionary success of species [28,34,36-39].

In *D. pulex*, STRs are predominantly found in introns with a clear preference for a small number of repeat types (AC, AG, AAG, AGC). Interestingly, all mono- to trinucleotide repeat types are densest in introns, with the exception of AT and CCG repeat types. A predominance of STRs in introns has not been reported for many genomes before, except e.g. for fungi in [9]. In *D. melanogaster*, STRs have highest densities in 3'UTR with a preference for AG, AT, AAC, and AGC repeats. Common to the *D. pulex *and *D. melanogaster *genome is the dominance of AC repeats in introns, AT repeats in 3'UTR, and CCG repeats in coding regions. Relatively high densities of CCG repeats in CDS regions and low densities in introns had also been reported for vertebrates and arthropods [9]. All these features are in contradiction to a model of neutral evolution of different TR types, see also [9,34]. They suggest differential selection to prevail in different genomes and genomic regions, which in turn hints at an evolutionary or functional importance of TRs.

Concerning the density of different repeat classes in different genomic regions of *D. pulex*, the following observations are of particular interest: (i) The densities of the repeat classes 1-5, 7-8 bp are higher in introns than in CDS and intergenic regions. (ii) The densities of TRs with a unit size above 8 bp are much lower in introns than in the other regions. (iii) The densities of almost all repeat classes with a unit size longer than 10 bp that are a multiple of three are higher in CDS regions than in introns and even intergenic regions. (iv) The high density of trinucleotide repeats in introns raises the question how well introns have been annotated. Furthermore it would be interesting to determine DNA transfer rates between CDS regions and introns caused by mutations. This process could also be the reason for higher trinucleotide densities in introns. Observation (i) could be explained by a preference for TRs in introns that are more variable or that have higher repeat copy numbers, which both could be important for regulatory elements. Observation (ii) could indicate that TRs with longer motifs are not beneficial in introns. Alternatively, the restricted size of introns could be the limiting factor for TRs with longer motifs. Observation (iii), however, shows that the size of genomic features does not provide a good indication for the expected motif sizes of TRs. While introns and CDS regions have about the same size in *D. pulex*, (see Table 5) observations (i) to (iii) show opposite preferences for the motif size of TRs in these two regions. The tendency toward longer repeat motifs in coding regions is presumably caused by tandemly repeated amino acid sequences, in particular for the motif PPR (proline - proline - glycin) and suggests strong protein domain level selection. Most interestingly, the absolute density of TRs with a unit size of 7-50 bp in CDS regions of *D. pulex *is higher than in CDS regions of *D. melanogaster*, despite of the strong tendency towards longer repeat units in all other regions of *D. melanogaster*.

An interesting observation of our analysis is the strandedness found for some repeat motifs in CDS regions and introns. The fact that some motifs are favoured on a particular strand hints at a selective advantage that remains to be studied in more detail.

The overall strong differences in TR characteristics in genomes and genomic regions raises many questions. For the extreme outlier in respect to repeat type usage, *O. lucimarinus*, we found that the most dominant repeats have a high CG content, which correlates with the high CG content of the complete genome. It would certainly be interesting to study this putative correlation in a separate study. An observation of Riley et al. [33,72] should be noted at this point. They have found that for repeats with putative regulatory function, the existence of the repeat and its overall structure is more important than the detailed base composition. This would allow organisms to have different repeat motifs with their preferred base composition at regulatory important segments of the genome.

### Finding annotation problems with TRs

The question arises whether TRs can be used to detect problems or inconsistencies in the current annotation of genomes. For this reason we had a closer look at selected TRs occurring in coding regions of *D. pulex *(from Additional file 6). Only a small proportion of these annotated genes show a clearly low support, but the support deceased for annotated gene, which host multiple TRs, such as e.g. Dappu-243907 and Dappu-318831. Furthermore, we had a look at gene models that host TRs with a motif size that is not a multiple of three, e.g. the relatively dense 10 and 20 bp repeat classes. Among these gene models, several were found for which the TR has almost the same size as the CDS element. Interesting examples with almost identical repeat units are found in the following annotated genes (braces contain the length of the CDS element, the length of the TR as well as the repeat unit): Dappu-264024 (1075 bp, 1033, ACGCCAGAGC), Dappu-264706 (165 bp, 113 bp, ACGCCAGAGC), Dappu-267284 (414 bp, 395 bp, ACGCCAGTGC), Dappu-267285 (460, 459, ACGCCAGTGC), and Dappu-265168 (738 bp, 473 bp, AATGC***ACGCCAGTGC***ACGCC). The numbers show that these CDS elements consist almost exclusively of the repeat pattern. The unit ACGCCA is indeed found in several other TRs in CDS regions of *D. pulex*. We found that the mean perfection of these 10-mer repeats (97.4%) is only marginally lower than that of 9-mer repeats (98.8%) or that of trinucleotide repeats (99.1%), indicating that their imperfection should not be an indication for a potential invariability of these 10-mer repeats in CDS regions. Another problematic finding is the high repeat content in exons of *D. melanogaster *of the two very similar repeat types with the unit AAACCAACTGAGGGAACGAGTGCCAAGCCTACAACTTTG (195.4 bp/Mbp) and AAACCAACTGAGGGAACTACGGCGAAGCCTACAACTTTG (109.1 bp/Mbp) with no contribution of these repeat types neither to CDS or UTRs, hinting at a problem in the annotation where these repeats occur.

### Error margins

For the characteristics of TRs analysed in the present work we have not given any error margins, not because we do believe that our results are exact, but since an estimate of error margins is hardly feasible. While a minor source of uncertainty might be introduced by the TR search algorithm, the main source of error is the incomplete nature of most genome assemblies (see Table 1). The genomic sequences of the current assembly of *D. pulex*, *A. mellifera*, *D. melanogaster*, and *H. sapiens *for instance contain 19.6%, 15.6%, 3.8%, and 7.2% unknown nucleotides (Ns), respectively (Table 1). But even the apparently low number of Ns in the latter two organism might be too optimistic, which is phrased in [62] as follows: "... a telomere-to-telomere DNA sequence is not yet available for complex metazoans, including humans. The missing genomic "dark matter" is the heterochromatin, which is generally defined as repeat-rich regions concentrated in the centric and telomeric regions of chromosomes. Centric heterochromatin makes up at least 20% of human and 30% of fly genomes, respectively; thus, even for well-studied organisms such as *D. melanogaster*, fundamental questions about gene number and global genome structure remain unanswered."

For obvious reasons, most genome projects focus on sequencing easily accessible coding regions and leave aside highly repetitive regions which are difficult to sequence and assemble. As a consequence, TRs densities will be lower in sequenced than in unsequenced genomic regions, and error margins for TR densities cannot be assessed statistically, but depend on mostly unknown systematic errors of the current assembly. The implications for the present work are, that TR densities are likely to be underestimated for all genomes analysed. Among the three arthropods, *D. melanogaster *is the best-studied organism and the only one with an exclusive *Heterochromatin Genome Project *[61,62]. For *D. pulex *and *A. mellifera*, heterochromatic regions have not yet been sequenced with the same effort. However, the contribution of heterochromatin in *A. mellifera *is estimated to be about 3% [73,74], whereas in *D. melanogaster *the contribution is about 30%, without clear boundaries between euchromatin and heterochromatin [75]. These differences in sequencing status and different sizes of heterochromatic regions could lead to a bias of yet unknown direction.

Altogether, it is expected that this bias will not affect the generally robust trends we found in our analyses for the following reasons: in *D. melanogaster*, the trend towards longer repeats units appeared already in the first assemblies, while this has not been observed in *A. mellifera*. In this context it is interesting to note that the total density of STRs is still higher in *A. mellifera *than in *D. melanogaster*. In *D. pulex*, no reliable estimate of the contribution of heterochromatin is known. Our study indicates a trend to slightly higher contributions than in *A. mellifera*, but considerably lower contributions than in *D. melanogaster*.

## Conclusions

The newly sequenced genome of *Daphnia pulex *shows several interesting characteristics of TRs which distinguish it from the other model arthropods *D. melanogaster *and *A. mellifera*. The density of TRs is much lower than in the two other arthropods. The mean length of STRs was shortest among all genomes in this study. From a functional perspective it is interesting that STRs are by far densest in introns and that the contribution of TRs with units longer than 6 bp in CDS regions of *D. pulex *is even higher than in *D. melanogaster*. The finding of a strong strand bias in repeat motif usage (strandedness) underpins the functional relevance of several repeats. A notable feature of *D. pulex *is the high density of 17 bp repeats presumably associated to heterochromatin regions.

Comparing the 12 genomes, our results reveal an astonishing level of differences in TR characteristics among different genomes and different genomic regions, which even exceeds the level of differences found in previous studies. Extreme "outliers" concerning densities and repeat type usage (*O. lucimarinus*), even lead us to the conjecture that nature has not imposed general limitations concerning repeat type usage and densities of TRs in genomes. In view of several general and lineage specific TR characteristics that have been refuted in this analysis and in view of the still small number of taxa that have been compared, the existence of common TR characteristics in major lineages becomes doubtful.

Altogether, this study demonstrates the need to analyse not only short TRs but also TR with longer units, which contribute significantly to all genomes analysed in this study. Restricting an analysis to STRs leaves a great amount of genomic TRs go unnoticed that may play an important evolutionary (functional or structural) role.

## Abbreviations

CDS: coding sequence; Ns: unknown nucleotides; STR: short tandem repeat; TR: tandem repeat; UTR: untranslated region

## Authors' contributions

CM developed the software Phobos and Sat-Stat as well as the programs to extract sequence segments with annotated genomic features. CM and FL conducted the computational experiments and analysed the data. CM, FL, and RT drafted the manuscript. All authors read and approved the final manuscript.

## Supplementary Material

Additional file 1 Comparison of Phobos search parameters used in this analysis and default parameters of the Tandem Repeats Finder (TRF) program. Click here for file

Additional file 2 Density and mean length of STRs versus genome size, divided into perfect and imperfect repeats. Click here for file

Additional file 3 Genomic densities, mean lengths, number of satellites and mean perfection for tandem repeat classes in all twelve genomes. Click here for file

Additional file 4 **Genomic density (grey columns) and mean length (black line) of TRs with a unit size of 1- 50 bp in *Daphnia pulex*.** The mean length of short tandem repeats is comparatively small. Repeat units with a unit size of 17 bp and 34 bp have much longer mean lengths than other repeats of similar unit size. Click here for file

Additional file 5 Genomic density, mean lengths, number of satellites, and mean perfection of TR classes in different genomic regions of *Daphnia pulex*, the euchromatic genome of *Drosophila melanogaster*, and *Apis mellifera*. Click here for file

Additional file 6 Table of tandem repeats found in coding regions of *Daphnia pulex*. Click here for file

Additional file 7 **Genomic density, mean lengths, number of satellites and mean perfection of individual TR motifs in different genomic regions of *Daphnia pulex*, the euchromatic genome of *Drosophila melanogaster *and *Apis mellifera*. **Only repeats in the unit size range 1-50 bp are shown that have a minimum density of 50 bp/Mbp in one genomic region of the three genomes. The second column in the table assists detecting motif pairs that differ only by the reverse complement. An A stands for the normal form and B for the reverse complement of motif A. "-" indicates palindromic motifs for which the unit is identical to its reverse complement (e.g. AT). If only one of the two motifs is present, the letters a and b are used. Thus "a" indicates that only the normal form is present, whereas "b" indicates that only the reverse complement of the normal form was found. Click here for file
