# Supplementary material for: Genome-wide analysis of tandem repeats in Daphnia pulex - a comparative approach
Source: BMC Genomics. 2010 Apr 30;11:277. doi: 10.1186/1471-2164-11-277 (PMC3152781; doi:10.1186/1471-2164-11-277)
Supplement: Additional file 1 — Comparison of Phobos search parameters used in this analysis and default parameters of the Tandem Repeats Finder (TRF) program. [file 1471-2164-11-277-S1.PDF]

Additional File 1: Comparison of PHOBOS search parameters used in this analysis and default parameters of the Tandem Repeats Finder (TRF) program.

Both the TRF and the PHOBOS program use the alignment score as the optimality criterion for a tandem repeat. The optimality criterion is used to decide which of two versions of the same repeat is preferred. One notable difference is that PHOBOS does not score the first unit with the desired effect of shorter units being slightly favoured in the case where two alignments with alternative units are being compared.
Furthermore, the TRF uses a pre-selection of tandem repeats specified in the form of a match and an indel probability. These two values specify the required identity and maximum proportion of indels allowed in adjacent units of the repeat. This pre-selection uses a probabilistic approach designed such that the TRF finds 95% of the repeats with these characteristics. TRF then reports those repeats that have a specified minimum score.
In contrast to TRF, PHOBOS uses an exact (i.e. non-probabilistic) approach with the intention to find all repeats that meet the specified tandem repeat characteristics. These characteristics are specified in form of a minimum percentage perfection, as well as a minimum score and/or length. Both, the minimum score and length can be specified as a linear (affine) function of the repeat unit size.

As mentioned in the Methods section, the PHOBOS parameters used in the present analysis were 1, -5, -5 for a match, mismatch or gap position, respectively. TRs have been considered in the analysis if they had a minimum repeat alignment score of 12 in the case the unit size was less or equal to 12 bp or a score of at least the unit size for unit lengths above 12 bp. As a consequence (and since the first unit is not scored), perfect mono-, di-, and trinucleotide repeats were required to have a minimum length of at least 13, 14, and 15 bp to achieve the minimum score. For units longer than 12 bp a perfect repeat needed to be at least 2 units long. Imperfect repeats needed to be even longer.

In contrast, the default values in the web interface of the TRF program are: 2, -7, -7, for match, mismatch, and gap positions. A minimum alignment score can be chosen in the range from 30-50. Since with these parameters the mismatch and gap score are smaller in comparison to the match score, a search with TRF using these default parameters will find more highly imperfect repeats than we find in our analysis. Due to a higher value of the minimum score, repeats with shorter units need to be longer when using these parameters than in our analysis. For a minimum score of 30, the minimum length of perfect repeats must be 15 bp for all unit sizes. However, repeats with longer units need to be 1.8 units long.

Even though, PHOBOS can also detect repeats with longer units, the unit size range for most comparisons conducted in our analysis was 1-50 bp. In contrast, the default unit size range of the TRF program is 1-500 bp.

Advantages of PHOBOS over TRF:
When developing PHOBOS, we have put particular emphasis in accurately detecting repeats with a relatively short unit and also a low copy number. In this regime, the probabilistic algorithm of TRF is most likely to fail. Using the TRF with a default minimum score of 30 to 50, repeats are expected to be sufficiently long so that this effect can be considered to be small. When searching for imperfect repeats with the typical unit size range of 1-10 bp, PHOBOS is much faster than TRF.

Advantages of TRF over PHOBOS:
For very long repeat units, TRF is more efficient than PHOBOS. For long units, the accuracy has not been compared yet.
