# Supplementary material for: Genome-wide analysis of tandem repeats in Daphnia pulex - a comparative approach
Source: BMC Genomics. 2010 Apr 30;11:277. doi: 10.1186/1471-2164-11-277 (PMC3152781; doi:10.1186/1471-2164-11-277)
Supplement: Additional file 2 — Density and mean length of STRs versus genome size, divided into perfect and imperfect repeats. [file 1471-2164-11-277-S2.PDF]

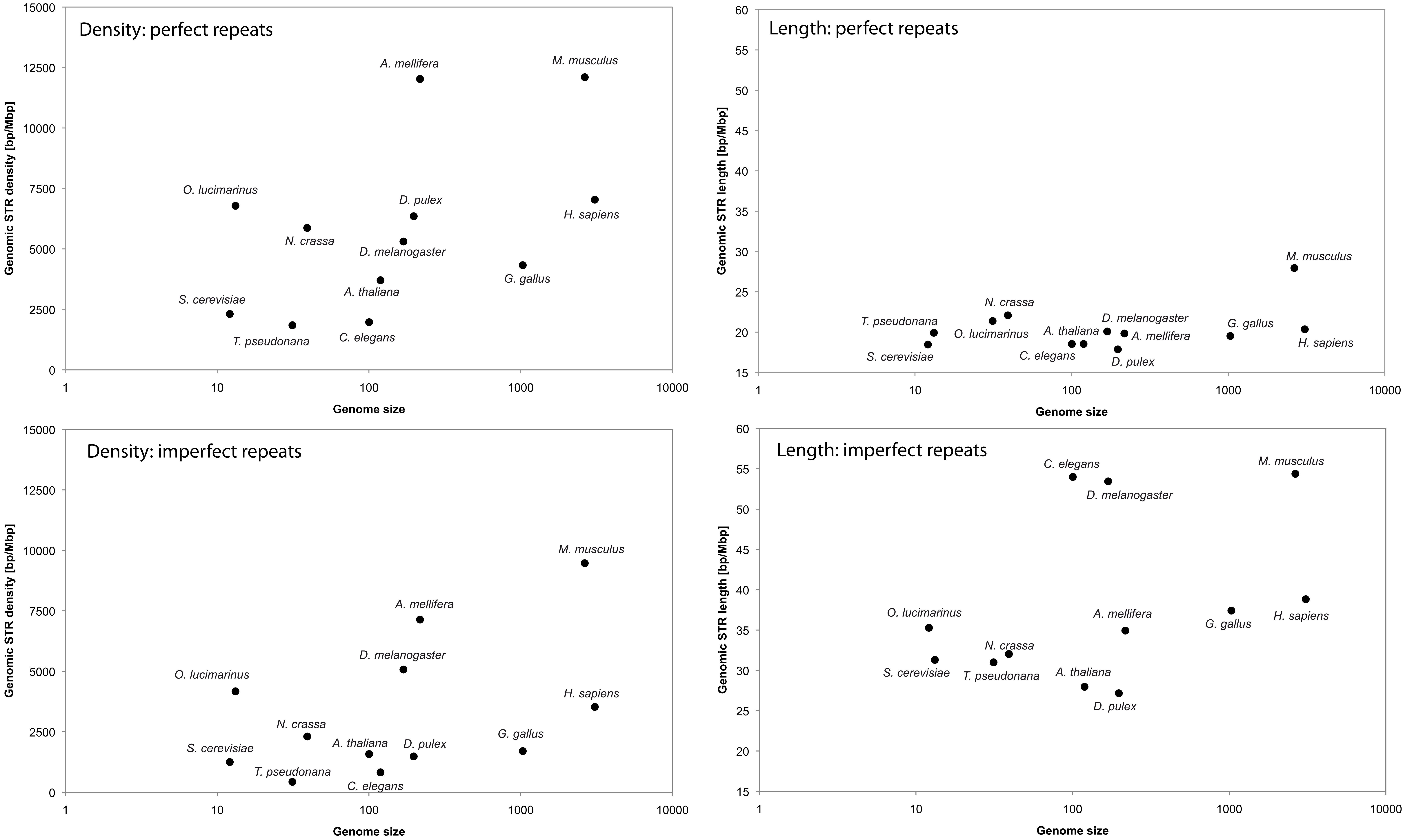

Additional File 2: Density (left) and mean length (right) of STRs versus genome size, divided into perfect (upper charts) and imperfect (lower charts) repeats.
