# Supplementary material for: Genome-wide analysis of tandem repeats in Daphnia pulex - a comparative approach
Source: BMC Genomics. 2010 Apr 30;11:277. doi: 10.1186/1471-2164-11-277 (PMC3152781; doi:10.1186/1471-2164-11-277)
Supplement: Additional file 3 — Genomic densities, mean lengths, number of satellites and mean perfection for tandem repeat classes in all twelve genomes. [file 1471-2164-11-277-S3.PDF]

Additional file 3: (a) Densities of TRs in repeat classes of all twelve genomes

unit-size	Dappu v1.1 bp/Mbp	DroMel-5.5 bp/Mbp	ApiMel-4.0 bp/Mbp	CaeEle-WS160 bp/Mbp	HomSap-36.2 bp/Mbp	MusMus-36.1 bp/Mbp	GalGal-2.1 bp/Mbp	AraTha-6.0 bp/Mbp	ThaPse-3.0 bp/Mbp	OstLuc-2.0 bp/Mbp	NeuCra-7.0 bp/Mbp	SacCer-2.1 bp/Mbp	min bp/Mbp	max bp/Mbp
**1**	1747.05	1638.96	4166.41	653.35	3889.59	2107.50	2617.82	1468.67	13.04	5.00	1971.02	1157.74	5.00	4166.41
**2**	1884.78	1961.64	7860.77	727.27	2267.41	8913.75	625.82	1264.85	235.75	3075.53	535.49	646.68	235.75	8913.75
**3**	2661.97	1543.72	3181.89	480.56	658.69	1877.24	450.07	1052.81	1457.03	3980.72	2752.73	900.43	450.07	3980.72
**4**	632.86	939.20	1742.14	224.94	2248.99	5360.09	1137.97	158.94	187.96	553.66	1095.23	118.55	118.55	5360.09
**5**	534.78	3253.74	1313.43	120.24	1032.11	1994.93	964.77	253.66	66.35	1160.18	619.32	133.38	66.35	3253.74
**6**	418.03	1538.05	1008.61	1372.97	511.48	1517.09	330.23	350.00	385.34	2223.80	1250.61	639.14	330.23	2223.80
**7**	246.38	1291.02	914.68	516.49	336.14	525.14	183.56	628.74	68.04	1602.74	277.98	81.10	68.04	1602.74
**8**	245.32	846.53	1003.27	646.63	304.20	823.71	428.91	118.92	243.15	898.30	306.26	84.75	84.75	1003.27
**9**	380.14	577.69	1054.88	801.62	236.09	336.77	154.76	112.27	74.69	1966.24	629.19	322.84	74.69	1966.24
**10**	572.33	473.75	1351.75	614.65	269.68	516.09	197.02	117.73	40.06	832.80	216.08	95.02	40.06	1351.75
**11**	234.00	6268.95	997.53	1172.56	176.97	220.16	171.08	109.34	36.00	818.79	202.36	125.43	36.00	6268.95
**12**	404.86	2272.43	1120.55	437.84	292.88	528.97	213.17	177.88	147.32	2077.34	536.20	371.89	147.32	2272.43
**13**	174.72	112.97	595.97	171.77	156.95	191.59	106.89	83.41	29.46	518.75	188.34	75.55	29.46	595.97
**14**	109.67	147.28	384.99	123.30	173.36	284.12	85.97	58.15	130.68	377.13	73.56	36.70	36.70	384.99
**15**	249.76	161.12	299.43	721.62	157.36	275.05	77.65	85.31	130.87	653.92	258.56	170.74	77.65	721.62
**16**	76.86	62.24	181.20	323.04	182.25	262.95	75.83	67.89	25.38	209.77	64.30	5.80	5.80	323.04
**17**	650.40	34.06	121.91	271.35	143.61	188.04	52.76	74.10	46.99	119.73	46.58	16.15	16.15	650.40
**18**	196.10	88.47	147.11	312.89	177.03	223.17	50.23	100.43	77.48	377.81	142.29	117.22	50.23	377.81
**19**	106.09	43.01	77.65	575.95	117.56	154.57	33.42	47.71	14.68	79.89	25.62	16.82	14.68	575.95
**20**	83.21	54.09	109.49	1048.63	167.71	231.89	44.99	66.69	56.70	88.45	29.55	36.04	29.55	1048.63
**21**	162.30	111.73	75.21	654.99	119.19	156.64	32.60	120.39	64.10	232.94	55.61	58.98	32.60	654.99
**22**	40.72	122.84	47.25	182.24	115.55	176.17	22.85	56.70	6.63	39.46	12.72	0.00	0.00	182.24
**23**	47.33	553.69	55.51	89.15	109.82	115.84	16.81	46.80	34.74	21.43	5.18	17.40	5.18	553.69
**24**	336.99	88.41	93.40	50.91	179.23	177.82	16.00	111.08	55.10	247.26	31.13	84.83	16.00	336.99
**25**	22.59	31.14	47.62	623.15	102.89	107.47	13.86	42.47	10.44	27.49	5.79	17.56	5.79	623.15
**26**	37.39	34.65	200.86	639.95	137.71	127.14	9.21	138.36	31.83	57.48	6.58	0.00	0.00	639.95
**27**	86.97	46.73	58.80	342.08	114.22	134.80	11.65	263.77	30.10	98.90	12.75	30.74	11.65	342.08
**28**	22.39	38.00	75.73	53.78	151.47	127.21	20.88	308.45	9.20	25.98	11.96	0.00	0.00	308.45
**29**	15.56	14.11	40.00	130.12	116.61	127.68	10.27	70.85	1.89	6.66	27.61	0.00	0.00	130.12
**30**	110.51	80.52	87.47	230.31	146.99	161.34	13.59	124.61	34.71	96.25	24.65	40.68	13.59	230.31
**31**	56.62	181.65	31.80	391.56	115.02	158.31	9.19	96.56	24.87	22.72	0.00	0.00	0.00	391.56
**32**	39.92	67.85	35.94	746.33	143.44	136.35	9.71	157.45	2.05	5.45	3.72	0.00	0.00	746.33
**33**	80.85	315.82	43.61	278.12	103.98	101.06	6.96	65.59	15.26	129.88	12.62	15.57	6.96	315.82
**34**	80.81	139.60	53.91	807.92	137.61	104.60	4.82	20.56	2.57	0.00	0.00	12.34	0.00	807.92
**35**	82.93	130.77	57.16	1913.16	108.22	79.51	8.74	49.66	2.92	24.38	8.21	0.00	0.00	1913.16
**36**	79.28	85.31	285.87	267.25	127.17	106.47	7.52	66.76	27.60	101.93	30.34	728.69	7.52	728.69
**37**	24.70	19.44	44.96	149.58	158.69	89.06	4.10	29.67	8.98	21.43	0.00	0.00	0.00	158.69
**38**	39.08	23.37	27.96	155.51	125.10	112.93	6.90	38.54	3.72	35.21	2.19	0.00	0.00	155.51
**39**	37.79	153.51	37.98	282.65	124.77	103.95	9.83	37.01	10.25	82.32	5.79	25.68	5.79	282.65
**40**	9.30	19.86	47.00	847.74	176.08	86.76	12.72	17.46	10.87	74.29	8.41	0.00	0.00	847.74
**41**	20.68	4.57	26.31	346.74	92.43	66.53	13.69	12.92	16.02	6.36	0.00	118.30	0.00	346.74
**42**	61.60	30.42	25.78	218.70	87.56	106.76	9.71	44.67	32.15	200.61	0.00	118.22	0.00	218.70
**43**	31.36	22.45	17.42	366.26	62.58	52.03	13.78	34.21	0.00	15.68	2.80	0.00	0.00	366.26
**44**	11.54	22.55	29.55	98.18	68.17	61.98	8.58	22.33	0.00	0.00	0.00	0.00	0.00	98.18
**45**	73.75	80.73	43.16	228.68	57.89	50.01	5.61	49.20	6.28	28.63	8.92	0.00	0.00	228.68
**46**	25.23	152.10	31.15	24.00	60.84	64.81	1.53	15.35	3.01	50.89	0.00	0.00	0.00	152.10
**47**	23.71	36.03	20.18	44.01	87.18	43.57	2.81	10.57	3.72	0.00	0.00	30.07	0.00	87.18
**48**	82.42	83.25	20.82	220.51	144.03	51.66	6.01	60.49	10.35	15.07	3.26	29.33	3.26	220.51
**49**	20.65	20.42	30.60	26.28	148.67	38.60	2.03	25.66	0.00	10.53	4.82	0.00	0.00	148.67
**50**	40.13	15.12	43.63	83.08	85.79	51.12	3.38	34.67	4.04	0.00	0.00	0.00	0.00	85.79

Additional file 3: (b) Mean lengths of TRs in repeat classes of all twelve genomes

unit-size	Dappu v1.1 [bp]	DroMel-5.5 [bp]	ApiMel-4.0 [bp]	CaeEle-WS160 [bp]	HomSap-36.2 [bp]	MusMus-36.1 [bp]	GalGal-2.1 [bp]	AraTha-6.0 [bp]	ThaPse-3.0 [bp]	OstLuc-2.0 [bp]	NeuCra-7.0 [bp]	SacCer-2.1 [bp]	min [bp]	max [bp]
**1**	16.15	16.95	17.79	15.96	18.79	18.78	18.05	16.66	14.03	13.20	24.04	17.25	13.20	24.04
**2**	18.57	21.95	26.31	24.28	28.93	40.42	22.29	23.26	21.40	19.80	22.97	23.31	18.57	40.42
**3**	19.69	26.08	26.29	18.75	24.29	37.74	20.47	20.83	20.49	23.82	24.11	26.47	18.75	37.74
**4**	21.71	32.06	24.09	24.34	32.24	39.21	28.75	19.26	24.74	25.67	24.04	21.36	19.26	39.21
**5**	24.45	89.30	26.45	20.72	28.25	36.60	39.54	19.60	20.50	25.17	24.35	22.69	19.60	89.30
**6**	27.84	30.55	26.96	56.81	26.49	41.64	35.04	22.73	59.51	26.21	26.13	30.19	22.73	59.51
**7**	23.49	41.27	27.10	33.07	27.48	34.66	28.37	47.64	24.98	40.18	25.06	23.88	23.49	47.64
**8**	24.47	43.37	27.03	34.26	30.82	41.18	28.42	24.44	101.01	27.11	30.38	24.36	24.36	101.01
**9**	27.39	39.58	28.53	32.53	37.63	40.66	29.58	25.21	26.48	28.85	28.33	31.50	25.21	40.66
**10**	45.20	50.17	32.68	34.35	35.06	40.46	30.75	28.07	28.41	29.35	30.76	26.67	26.67	50.17
**11**	27.78	369.57	29.26	47.85	35.05	36.81	29.66	28.66	28.07	30.98	31.55	32.91	27.78	369.57
**12**	35.74	161.36	31.18	35.54	37.92	42.21	31.02	31.25	31.92	32.43	33.61	37.41	31.02	161.36
**13**	35.26	34.61	34.42	35.66	44.13	46.74	33.25	35.45	35.35	34.08	44.31	43.43	33.25	46.74
**14**	37.90	57.12	37.35	44.86	46.67	55.46	37.63	37.77	69.10	36.89	37.96	55.38	36.89	69.10
**15**	50.92	63.52	48.80	125.09	58.58	60.81	43.91	42.40	54.30	40.49	50.54	49.19	40.49	125.09
**16**	68.93	56.45	49.17	149.96	65.12	70.29	52.69	50.81	41.68	40.14	51.71	35.00	35.00	149.96
**17**	270.15	60.12	50.62	89.48	71.98	84.94	51.29	46.16	61.08	40.54	50.75	48.75	40.54	270.15
**18**	85.90	67.99	67.14	92.36	80.08	87.92	55.39	54.63	71.09	48.91	64.45	64.77	48.91	92.36
**19**	59.36	63.48	58.06	195.15	83.52	109.38	54.35	52.56	45.80	45.87	50.25	50.75	45.80	195.15
**20**	85.16	98.66	90.70	334.67	99.10	102.55	69.68	59.01	61.00	61.47	55.19	108.75	55.19	334.67
**21**	70.93	204.72	78.65	227.28	100.28	124.66	72.55	62.60	58.82	59.15	64.15	72.60	58.82	227.28
**22**	72.34	443.20	72.78	112.31	97.58	139.71	70.18	61.90	51.75	52.10	62.38			443.20
**23**	97.68	553.91	108.69	144.18	112.99	140.26	69.56	60.53	120.44	56.60	50.75	70.00	50.75	553.91
**24**	152.93	110.67	127.69	82.34	114.76	132.86	76.87	80.28	71.62	85.92	67.83	128.00	67.83	152.93
**25**	83.35	248.39	130.96	833.13	132.53	145.47	82.22	78.97	54.33	60.50	56.75	70.67	54.33	833.13
**26**	95.66	125.00	226.09	588.72	154.88	154.17	93.47	144.06	141.86	75.90	64.50			588.72
**27**	107.90	93.86	138.61	357.31	175.01	173.82	98.09	180.35	67.07	72.56	83.33	74.20	67.07	357.31
**28**	84.60	134.13	166.98	158.62	182.47	168.43	139.47	239.90	57.40	68.60	93.80			239.90
**29**	85.07	163.71	144.42	383.74	201.32	194.41	121.87	136.15	59.00	88.00	216.60			383.74
**30**	152.46	146.89	150.72	231.93	201.45	204.20	125.76	116.59	72.20	84.73	107.44	98.20	72.20	231.93
**31**	94.55	581.38	166.95	265.81	212.87	290.37	113.11	117.26	86.22	75.00				581.38
**32**	93.14	196.71	148.27	409.41	220.27	207.84	127.91	127.46	64.00	72.00	73.00			409.41
**33**	152.21	457.87	146.36	224.93	234.99	205.23	114.20	111.50	79.33	131.92	123.75	94.00	79.33	457.87
**34**	210.16	444.41	161.77	248.70	279.41	218.59	113.02	84.48	80.00			149.00		444.41
**35**	139.95	849.28	164.52	307.70	273.98	194.97	141.13	109.43	91.00	80.50	107.33			849.28
**36**	155.41	231.07	183.84	282.08	253.09	224.59	145.24	120.53	107.62	149.56	170.00	233.95	107.62	282.08
**37**	156.68	150.29	173.13	306.08	307.11	245.91	118.62	130.74	93.33	94.33				307.11
**38**	112.73	189.75	137.45	577.52	321.32	242.69	161.81	131.03	116.00	232.50	86.00			577.52
**39**	153.69	361.48	162.48	480.64	313.20	216.46	153.75	137.62	106.67	155.29	227.00	155.00	106.67	480.64
**40**	105.36	322.40	231.02	438.28	239.64	233.48	181.59	138.53	113.00	327.00	165.00			438.28
**41**	105.84	247.67	160.32	643.85	251.65	224.82	312.79	109.79	100.00	84.00		1428.00		1428.00
**42**	139.90	189.92	186.12	203.05	294.32	196.07	180.47	136.31	250.75	132.45		475.67		475.67
**43**	130.92	364.40	236.71	270.19	319.97	229.13	226.90	145.43		207.00	110.00			364.40
**44**	183.00	915.25	200.76	298.33	294.39	236.93	176.02	115.52						915.25
**45**	208.91	409.59	243.29	546.19	334.30	245.28	221.33	130.09	98.00	189.00	175.00			546.19
**46**	363.91	2057.92	179.95	240.60	325.94	314.81	137.09	130.50	94.00	336.00				2057.92
**47**	313.50	619.58	233.10	1103.25	455.45	248.09	162.47	157.12	116.00			363.00		1103.25
**48**	335.33	1126.33	253.21	1228.39	464.36	260.71	197.30	206.17	161.50	99.50	128.00	354.00	99.50	1228.39
**49**	218.47	174.53	227.00	376.71	261.44	257.04	222.11	190.81		139.00	189.00			376.71
**50**	172.05	245.50	252.00	417.20	305.28	266.11	261.31	187.50	126.00					417.20

Additional file 3: (c) Numbers of TRs in repeat classes of all twelve genomes

unit-size	Dappu v1.1 #sat	DroMel-5.5 #sat	ApiMel-4.0 #sat	CaeEle-WS160 #sat	HomSap-36.2 #sat	MusMus-36.1 #sat	GalGal-2.1 #sat	AraTha-6.0 #sat	ThaPse-3.0 #sat	OstLuc-2.0 #sat	NeuCra-7.0 #sat	SacCer-2.1 #sat	min #sat	max #sat
**1**	17161	15701	54104	4104	591617	286206	142803	10492	29	5	3216	810	5	591617
**2**	16104	14511	69036	3004	224459	563483	27674	6471	344	2051	915	335	335	563483
**3**	21458	9622	28005	2570	77621	127365	21667	6017	2222	2208	4484	414	414	127365
**4**	4630	4770	16755	927	200797	351575	39190	982	237	285	1788	67	67	351575
**5**	3476	5984	11528	582	105107	140683	24240	1540	101	610	1000	71	71	140683
**6**	2382	8220	8673	2430	55324	93653	9382	1840	202	1124	1889	256	202	93653
**7**	1664	5133	7810	1567	35008	38803	6385	1577	85	527	435	41	41	38803
**8**	1591	3204	8585	1895	28276	51278	14888	579	76	438	417	42	42	51278
**9**	2213	2386	8557	2476	18011	21212	5159	530	88	902	876	127	88	21212
**10**	2041	1535	9572	1807	22085	32701	6358	499	44	375	276	43	43	32701
**11**	1336	2776	7884	2472	14492	15284	5685	454	40	350	252	46	40	15284
**12**	1814	2310	8313	1240	22202	32112	6778	678	144	846	629	120	120	32112
**13**	786	530	4001	483	10204	10500	3175	280	26	201	168	21	21	10500
**14**	459	419	2383	278	10654	13130	2253	185	59	135	76	8	8	13130
**15**	780	415	1421	581	7756	11604	1747	241	80	215	201	42	42	11604
**16**	177	179	852	219	8085	9568	1426	159	19	69	49	2	2	9568
**17**	389	92	558	313	5767	5700	1017	191	24	39	36	4	4	5767
**18**	366	212	509	340	6398	6519	895	219	34	102	87	22	22	6519
**19**	284	110	309	296	4115	3631	606	108	10	23	20	4	4	4115
**20**	155	89	279	338	4894	5825	645	136	29	19	21	4	4	5825
**21**	363	89	221	298	3453	3239	444	230	34	52	34	10	10	3453
**22**	91	45	150	164	3428	3342	323	109	4	10	8	0	0	3428
**23**	77	175	118	62	2815	2116	241	92	9	5	4	3	3	2815
**24**	350	130	169	62	4497	3428	205	165	24	38	18	8	8	4497
**25**	43	23	84	75	2254	1893	166	64	6	6	4	3	3	2254
**26**	62	45	214	109	2579	2116	99	117	7	10	4	0	0	2579
**27**	128	85	98	96	1898	2021	117	182	14	18	6	5	5	2021
**28**	42	46	106	34	2399	1942	149	153	5	5	5	0	0	2399
**29**	29	14	64	34	1674	1706	83	66	1	1	5	0	0	1706
**30**	119	89	136	100	2122	2032	109	128	15	15	9	5	5	2122
**31**	95	55	44	153	1554	1428	80	98	9	4	0	0	0	1554
**32**	70	56	56	187	1904	1690	76	147	1	1	2	0	0	1904
**33**	85	112	69	124	1271	1261	60	70	6	13	4	2	2	1271
**34**	61	51	77	330	1416	1221	42	29	1	0	0	1	0	1416
**35**	94	25	81	632	1138	1041	61	54	1	4	3	0	0	1138
**36**	81	61	360	95	1454	1217	51	66	8	9	7	41	7	1454
**37**	25	21	60	49	1506	927	34	27	3	3	0	0	0	1506
**38**	55	20	47	27	1117	1188	42	35	1	2	1	0	0	1188
**39**	39	69	54	59	1150	1237	63	32	3	7	1	2	1	1237
**40**	14	10	47	195	2106	951	69	15	3	3	2	0	0	2106
**41**	31	3	38	54	1051	757	48	14	5	1	0	1	0	1051
**42**	70	26	32	108	867	1389	53	39	4	20	0	3	0	1389
**43**	38	10	17	140	559	579	62	28	0	1	1	0	0	579
**44**	10	4	34	33	662	669	48	23	0	0	0	0	0	669
**45**	56	32	41	42	509	520	27	45	2	2	2	0	0	520
**46**	11	12	40	10	534	525	11	14	1	2	0	0	0	534
**47**	12	12	20	4	560	448	17	8	1	0	0	1	0	560
**48**	39	12	19	18	908	507	30	35	2	2	1	1	1	908
**49**	15	19	32	7	1629	383	9	16	0	1	1	0	0	1629
**50**	37	10	40	20	809	490	13	22	1	0	0	0	0	809

Additional file 3: (d) Mean percentage perfection of TRs in repeat classes of all twelve genomes

unit-size	Dappu v1.1 [%]	DroMel-5.5 [%]	ApiMel-4.0 [%]	CaeEle-WS160 [%]	HomSap-36.2 [%]	MusMus-36.1 [%]	GalGal-2.1 [%]	AraTha-6.0 [%]	ThaPse-3.0 [%]	OstLuc-2.0 [%]	NeuCra-7.0 [%]	SacCer-2.1 [%]	min [%]	max [%]
**1**	99.5	99.4	99.1	99.4	99.6	99.5	99.4	99.6	99.8	100.0	99.2	99.4	99.1	100.0
**2**	99.4	98.6	98.7	99.4	98.4	98.6	98.8	99.4	99.3	97.7	99.1	98.8	97.7	99.4
**3**	99.4	98.1	98.5	99.3	98.9	98.4	99.2	99.1	99.4	98.7	99.0	98.3	98.1	99.4
**4**	99.1	98.6	98.7	99.0	98.1	98.3	98.8	99.5	98.7	98.9	99.2	99.1	98.1	99.5
**5**	98.9	98.2	98.2	99.3	98.3	98.4	98.6	99.5	99.8	98.4	99.0	98.6	98.2	99.8
**6**	98.6	98.3	98.1	95.7	98.2	97.6	97.7	99.0	99.3	98.8	98.7	98.2	95.7	99.3
**7**	98.7	98.2	97.8	98.3	97.8	97.1	97.7	96.1	99.0	97.4	98.8	98.7	96.1	99.0
**8**	99.1	98.3	98.1	98.7	97.5	96.7	98.4	99.1	97.6	98.6	98.9	99.4	96.7	99.4
**9**	98.5	98.0	98.2	98.5	97.4	96.9	98.2	98.9	99.2	98.6	98.6	98.5	96.9	99.2
**10**	98.2	98.1	98.3	97.9	97.5	96.9	98.5	98.5	98.8	98.4	98.5	99.6	96.9	99.6
**11**	99.0	95.6	98.6	97.1	97.9	97.7	98.7	98.7	99.6	98.2	98.7	98.4	95.6	99.6
**12**	98.6	96.6	98.5	98.4	97.7	97.4	98.8	98.8	99.1	98.8	98.5	98.2	96.6	99.1
**13**	98.6	98.5	98.4	98.8	97.7	97.6	98.8	98.2	99.0	98.4	96.9	98.3	96.9	99.0
**14**	98.3	97.9	98.1	98.0	97.6	96.9	98.6	97.9	98.5	98.0	98.2	97.5	96.9	98.6
**15**	97.3	97.0	97.8	96.6	97.2	97.2	98.4	98.0	98.8	98.7	97.5	98.3	96.6	98.8
**16**	97.7	96.8	97.6	97.0	97.1	96.8	98.3	97.8	99.7	97.8	98.3	100.0	96.8	100.0
**17**	95.0	97.3	97.7	96.1	97.5	97.0	98.4	97.6	97.5	99.2	97.8	98.9	95.0	99.2
**18**	97.1	97.6	96.8	96.2	97.0	96.6	98.2	97.8	97.7	98.8	97.3	96.7	96.2	98.8
**19**	97.9	98.4	97.0	95.5	97.4	96.8	98.3	98.2	98.9	98.8	98.5	99.0	95.5	99.0
**20**	96.4	97.6	97.0	94.9	96.8	96.4	97.7	97.8	97.5	97.6	97.0	95.7	94.9	97.8
**21**	97.0	95.1	96.8	95.7	97.0	96.5	97.9	97.4	98.2	98.5	97.7	97.6	95.1	98.5
**22**	97.8	93.6	97.2	95.9	97.1	96.5	98.3	98.3	98.3	98.5	98.4			98.5
**23**	97.0	92.4	96.6	97.2	96.7	96.8	98.4	98.0	96.1	98.8	99.6	94.0	92.4	99.6
**24**	97.4	96.0	95.8	97.2	96.5	96.6	98.1	97.7	97.7	98.2	98.3	94.9	94.9	98.3
**25**	97.9	92.9	96.5	97.0	96.7	96.8	98.2	98.2	99.1	98.7	98.7	98.5	92.9	99.1
**26**	97.0	95.7	95.2	96.9	96.1	96.6	98.1	95.6	97.9	97.6	100.0			100.0
**27**	97.0	96.8	95.8	96.7	96.5	96.7	97.7	94.4	99.2	98.5	97.1	98.4	94.4	99.2
**28**	97.9	96.2	97.0	95.4	96.4	96.6	96.7	94.8	100.0	98.4	97.7			100.0
**29**	97.8	95.9	95.7	93.9	96.4	96.7	97.5	96.7	100.0	95.4	96.5			100.0
**30**	95.8	95.4	96.4	95.3	96.3	96.3	97.5	96.5	98.7	98.5	96.7	97.5	95.3	98.7
**31**	97.0	89.8	96.4	94.4	96.2	96.5	97.7	95.5	98.8	97.9				98.8
**32**	97.6	96.3	96.2	94.1	96.3	96.4	97.5	95.4	100.0	100.0	98.8			100.0
**33**	96.0	97.2	96.6	94.5	96.1	96.7	97.6	96.8	98.7	98.5	98.2	96.3	94.5	98.7
**34**	94.7	94.7	96.4	93.3	96.0	96.5	97.6	98.5	97.5			93.5		98.5
**35**	95.2	91.1	95.1	93.8	96.0	97.0	96.8	97.0	96.7	99.3	96.4			99.3
**36**	97.2	94.8	95.1	93.7	95.9	96.5	96.6	96.5	98.9	98.8	96.1	94.8	93.7	98.9
**37**	97.5	94.6	95.4	95.3	93.9	96.8	97.3	96.2	99.0	100.0				100.0
**38**	97.6	95.3	97.0	94.6	95.6	96.1	96.7	97.3	96.6	96.9	100.0			100.0
**39**	96.4	96.4	96.6	93.8	95.0	97.4	97.0	97.5	97.2	98.8	99.1	97.8	93.8	99.1
**40**	97.8	93.6	95.2	94.8	95.1	96.9	96.3	97.8	95.5	97.4	93.8			97.8
**41**	98.3	93.7	97.4	95.1	95.8	96.9	95.0	97.5	97.6	100.0		87.1		100.0
**42**	97.3	97.7	96.3	94.6	95.7	97.4	96.1	97.3	97.1	98.4		96.3		98.4
**43**	97.6	94.5	97.1	94.0	95.7	97.2	95.7	97.9		95.7	96.4			97.9
**44**	95.4	92.6	96.0	93.9	96.1	96.5	96.6	97.8						97.8
**45**	96.7	95.5	95.8	94.3	95.9	97.0	96.6	97.6	100.0	99.1	94.2			100.0
**46**	95.6	88.9	96.7	95.2	95.8	96.6	98.4	98.5	100.0	92.3				100.0
**47**	93.1	90.8	95.9	92.3	95.3	97.2	96.5	96.6	97.4			90.8		97.4
**48**	94.8	93.9	94.6	95.6	95.1	96.9	96.4	96.4	98.7	100.0	96.1	95.2	93.9	100.0
**49**	94.9	96.7	96.5	95.8	95.8	97.4	94.4	96.1		95.8	93.8			97.4
**50**	96.8	94.2	95.8	95.3	95.6	96.4	94.6	96.2	96.8					96.8
