# Supplementary material for: Genome-wide analysis of tandem repeats in Daphnia pulex - a comparative approach
Source: BMC Genomics. 2010 Apr 30;11:277. doi: 10.1186/1471-2164-11-277 (PMC3152781; doi:10.1186/1471-2164-11-277)
Supplement: Additional file 4 — Genomic density (grey columns) and mean length (black line) of TRs with a unit size of 1- 50 bp in Daphnia pulex. The mean length of short tandem repeats is comparatively small. Repeat units with a unit size of 17 bp and 34 bp have much longer mean lengths than other repeats of similar unit size. [file 1471-2164-11-277-S4.PDF]

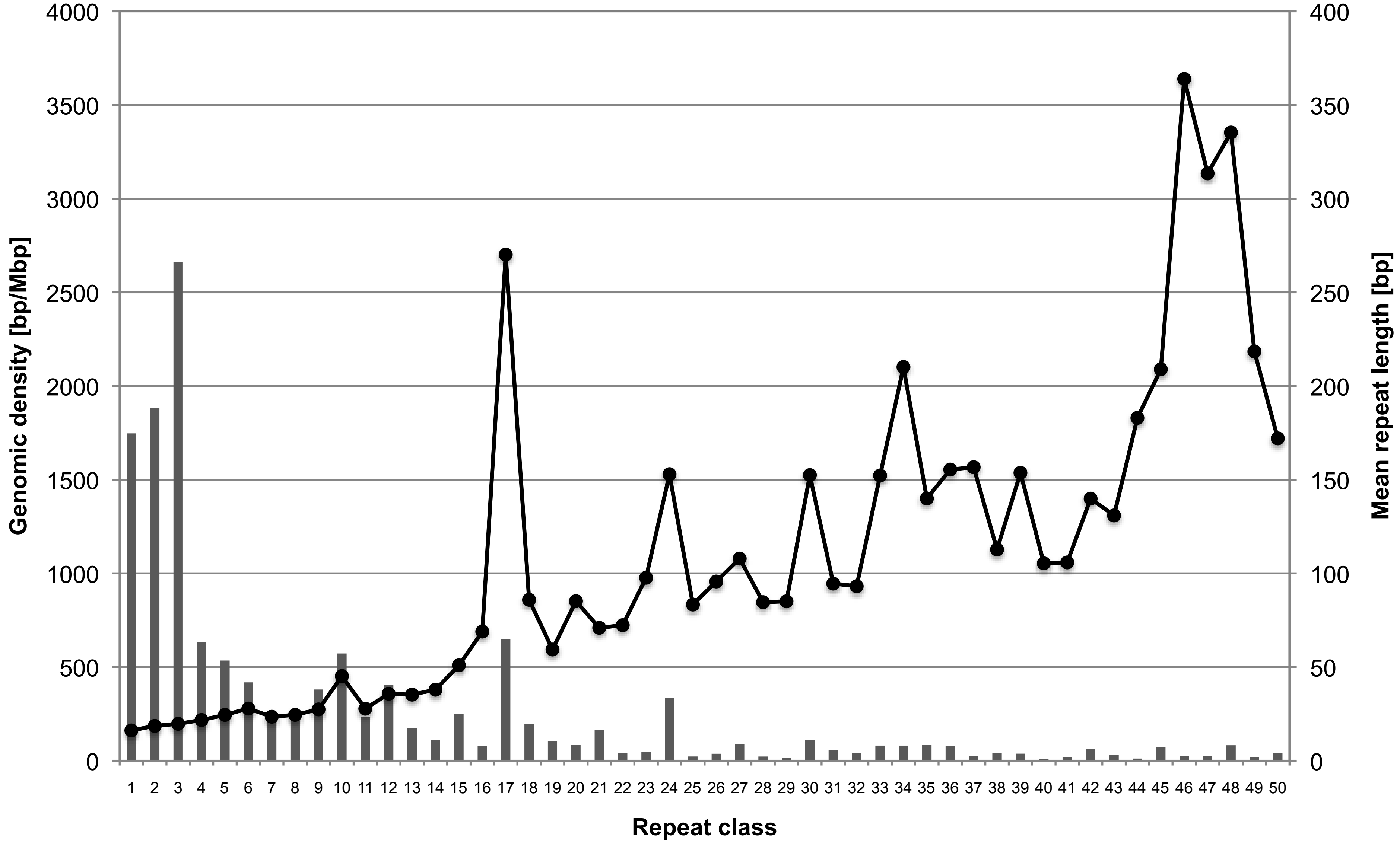

Additional File 4: Genomic density (grey columns) and mean length (black line) of TRs with a unit size of 1- 50 bp in *Daphnia pulex*. The mean length of short tandem repeats is comparatively small. Repeat units with a unit size of 17 bp and 34 bp have much longer mean lengths than other repeats of similar unit size.
