# Supplementary material for: Genome-wide analysis of tandem repeats in Daphnia pulex - a comparative approach
Source: BMC Genomics. 2010 Apr 30;11:277. doi: 10.1186/1471-2164-11-277 (PMC3152781; doi:10.1186/1471-2164-11-277)
Supplement: Additional file 5 — Genomic density, mean lengths, number of satellites, and mean perfection of TR classes in different genomic regions of Daphnia pulex, the euchromatic genome of Drosophila melanogaster, and Apis mellifera. [file 1471-2164-11-277-S5.PDF]

Additional file 5: (a) Genomic density of TRs in repeat classes in different genomic regions of *Daphnia pulex*, the euchromatic genome of *Drosophila melanogaster* and *Apis mellifera*.

unit size	Dappu v1.1 3'UTR [bp/Mbp]	Dappu v1.1 5'UTR [bp/Mbp]	Dappu v1.1 CDS [bp/Mbp]	Dappu v1.1 introns [bp/Mbp]	Dappu v1.1 intergenic [bp/Mbp]	DroMel-5.5 3'UTR [bp/Mbp]	DroMel-5.5 5'UTR [bp/Mbp]	DroMel-5.5 CDS [bp/Mbp]	DroMel-5.5 introns [bp/Mbp]	DroMel-5.5 intergenic [bp/Mbp]	ApiMel-2.0 CDS [bp/Mbp]	ApiMel-2.0 introns [bp/Mbp]	ApiMel-2.0 intergenic [bp/Mbp]	min [bp/Mbp]	max [bp/Mbp]
**1**	2566.71	649.89	27.09	3047.26	1878.28	1647.79	1159.25	8.83	2332.7	1790.14	135.1	4813.99	4204.54	8.83	4813.99
**2**	1832.65	1179.56	25.61	3280.74	2045.98	4145.34	2747.03	15.68	2763.69	2262.88	470.12	7188.96	7383.92	15.68	7383.92
**3**	1151.01	940.72	1568.72	3980.86	2659.33	1658.58	2301.5	2594.15	1419.65	1281.19	6130.3	3271	3119.31	940.72	6130.3
**4**	325.34	115.36	46.8	1080.71	690.49	917.7	724.22	5.49	882.8	742.53	151.89	1645.48	1772.09	5.49	1772.09
**5**	273.54	383.44	27.01	737.11	636.18	911.81	892.15	9.75	803.62	1418.64	89.42	1254.89	1318.3	9.75	1418.64
**6**	160.45	29.25	158.3	422.69	503.69	1788.07	1530.23	721.43	1639.88	1476.94	3333.4	1045.95	953.98	29.25	3333.4
**7**	125.72	201.46	38.27	336.21	288.58	662.16	774.52	28.72	1030.3	1073.82	147.99	976.98	910.13	28.72	1073.82
**8**	140.25	121.86	29.43	366.33	277.38	478.71	486.79	21.78	726.69	821.28	83.95	1129.84	980.45	21.78	1129.84
**9**	82.12	224.21	380.79	361.04	392.69	383.07	399.57	415.64	480.27	497.02	835.99	1089.58	1042.96	82.12	1089.58
**10**	164.25	0	294.7	381.32	725.34	245.24	144.77	4.3	310.98	318.76	1577.87	1318.4	1289.86	0	1577.87
**11**	262.17	183.59	31.06	261.25	288.27	142.98	170.11	127.34	266.18	5209.88	229.59	1100.93	977.12	31.06	5209.88
**12**	275.44	133.23	556.97	300.21	394.31	368.85	327.18	536.31	410.4	2133.43	668.48	1179.78	1087.53	133.23	2133.43
**13**	33.48	0	30.05	172.48	220.35	88.04	22.8	9.18	91.78	123.39	38.27	658.92	583.65	0	658.92
**14**	74.54	0	45.36	97.42	131.43	39.48	95.91	7.29	108.35	137.21	34.75	364.38	374.13	0	374.13
**15**	65.07	56.87	453.35	161.26	227.49	123.85	10.86	364.31	93.42	93.04	1547.02	289.88	267.13	10.86	1547.02
**16**	0	0	41.43	60.12	89.53	46.6	17.01	2.33	41.96	60.94	554.85	115.91	175.46	0	554.85
**17**	22.11	0	29.47	42.81	1039.42	48.56	12.31	4	27.22	36.64	57.01	152.24	117.1	0	1039.42
**18**	0	63.36	346.76	57.11	192.02	24.52	48.5	377.88	49.63	38.86	701.67	102.54	135.82	0	701.67
**19**	49.28	0	15.9	61.71	145.63	40.22	0	3.16	37.3	42.74	168.68	75.97	79.45	0	168.68
**20**	0	0	88.62	54.2	93.11	43.9	17.73	7.82	12.95	20.91	66.38	70.01	94.1	0	94.1
**21**	0	0	299.37	76.55	154.15	37.77	33.3	218.4	31.3	106.41	419.75	71.91	67.54	0	419.75
**22**	63.81	0	31.57	32.21	45.74	20.11	0	5.49	12.04	185.45	71.46	79.04	44.27	0	185.45
**23**	0	0	18.47	20.67	61.84	0	0	18.79	64.64	414.76	0	44.24	50.28	0	414.76
**24**	42.96	451.68	693.52	78.07	306.5	32.13	0	240.71	31.5	39.49	177.66	118.49	85.39	0	693.52
**25**	0	0	3.19	9.95	30.98	0	20.63	0	6.85	8.33	0	17.06	36.32	0	36.32
**26**	0	0	16.75	14.1	53.56	22.56	0	6.81	22.12	38.06	0	69.77	177.69	0	177.69
**27**	43.59	0	186.17	39.73	74.14	39.97	0	103.76	17.27	25.77	0	9.57	68.11	0	186.17
**28**	0	0	11.65	18.96	29.22	0	0	0	9.76	20.92	0	112.29	69.29	0	112.29
**29**	0	0	5.53	10.13	20.05	0	0	12.25	13.57	5.28	0	22.39	48.81	0	48.81
**30**	0	0	263.21	38.68	85.22	21.83	0	168.57	76.9	51.76	485.74	52.59	62.66	0	485.74
**31**	0	0	54.05	13.21	72.03	0	0	0	312.29	138.34	106.21	37.12	37.95	0	312.29
**32**	0	0	12.2	6.91	56.4	20.85	0	8.83	12.51	43.57	35.53	24.61	30.24	0	56.4
**33**	0	0	228.29	43.29	50.26	0	0	190.96	46.37	536	44.9	25.83	37.95	0	536
**34**	0	0	57.75	19.07	110.36	0	0	33.24	4.12	127.06	0	22.89	37.86	0	127.06
**35**	0	0	3.74	26.36	123.08	0	0	4.17	46.86	204.81	0	89.28	45.48	0	204.81
**36**	132.67	0	181.33	28.89	67.53	0	0	90.36	16.97	42.96	609.52	196.73	52.47	0	609.52
**37**	0	0	20.22	10.06	29.47	18.15	0	6.41	0	15.9	0	33.93	27.52	0	33.93
**38**	0	0	17.81	11.55	52.26	0	0	5.14	1.52	14.94	0	14.17	32.13	0	52.26
**39**	0	0	74.47	18.7	31.78	0	0	463.59	22.91	51.15	0	22.77	30.74	0	463.59
**40**	0	0	0	0	15.31	0	0	4.26	0	4.54	0	25.59	42.43	0	42.43
**41**	0	0	6.16	2.78	31.57	0	0	0	0	1.55	0	39.58	23.48	0	39.58
**42**	0	0	79.58	31.94	66.43	30.41	0	119.7	1.77	5.46	0	6.38	29.01	0	119.7
**43**	0	0	13.48	9.38	44.34	22.56	0	5.75	3.65	4.22	0	0	14.23	0	44.34
**44**	0	0	0	0	11.69	0	0	0	5.26	48.2	0	52.71	29.89	0	52.71
**45**	0	0	137.06	29.67	70.26	0	0	25.91	7.59	78.38	155.01	39.21	40.99	0	155.01
**46**	0	0	0	9.58	12.92	0	0	0	0	319.41	0	44.92	30.44	0	319.41
**47**	0	0	161.41	0	16.82	0	0	7.6	3.35	34.2	0	0	18.61	0	161.41
**48**	0	0	89.56	50.36	77.03	0	0	131.07	4.12	132.23	63.65	47.38	24.84	0	132.23
**49**	0	0	10.44	6.44	22.9	0	0	0	3.89	15.76	0	7.86	29.61	0	29.61
**50**	0	0	46.3	17.3	47.75	0	0	7.03	11.57	0	0	37.62	54.64	0	54.64

Additional file 5: (b) Mean lengths of TRs in repeat classes in different genomic regions of *Daphnia pulex*, the euchromatic genome of *Drosophila melanogaster* and *Apis mellifera*.

unit size	Dappu v1.1 3'UTR [bp]	Dappu v1.1 5'UTR [bp]	Dappu v1.1 CDS [bp]	Dappu v1.1 introns [bp]	Dappu v1.1 intergenic [bp]	DroMel-5.5 3'UTR [bp]	DroMel-5.5 5'UTR [bp]	DroMel-5.5 CDS [bp]	DroMel-5.5 introns [bp]	DroMel-5.5 intergenic [bp]	ApiMel-2.0 CDS [bp]	ApiMel-2.0 introns [bp]	ApiMel-2.0 intergenic [bp]	min [bp]	max [bp]
**1**	15.81	15.38	16.95	16.03	16.21	15.55	15.70	16.75	16.56	16.66	15.73	17.96	17.77	15.38	17.96
**2**	18.02	19.11	17.29	18.56	18.58	22.94	19.87	22.31	21.73	21.80	26.76	25.54	25.08	17.29	26.76
**3**	18.42	18.68	20.17	19.54	19.70	22.80	21.23	23.77	23.72	25.70	41.34	28.72	25.49	18.42	41.34
**4**	19.07	17.75	22.24	21.93	21.66	21.04	21.52	20.83	22.00	22.66	20.47	23.98	24.10	17.75	24.10
**5**	19.68	19.67	22.35	21.49	25.79	22.05	23.24	20.18	23.79	45.68	22.90	25.69	26.28	19.67	45.68
**6**	21.17	18.00	23.65	23.29	29.94	24.89	23.89	25.57	25.92	25.34	82.54	28.59	25.41	18.00	82.54
**7**	22.11	20.67	25.18	23.08	23.71	25.25	25.24	27.38	26.44	28.23	23.69	26.71	26.96	20.67	28.23
**8**	24.67	25.00	23.59	23.41	25.01	25.35	26.47	27.56	28.24	34.15	23.89	26.54	26.81	23.41	34.15
**9**	21.67	27.60	28.06	26.33	27.50	27.40	27.60	31.58	31.34	32.79	33.98	27.96	28.56	21.67	33.98
**10**	26.00		139.93	28.44	45.38	27.78	25.00	32.67	28.55	30.44	106.39	31.00	31.73	25.00	139.93
**11**	27.67	28.25	30.65	28.15	27.55	27.76	29.38	263.64	36.85	556.19	34.05	28.93	29.16	27.55	556.19
**12**	33.54	41.00	39.08	30.69	35.61	32.00	33.48	38.24	35.87	175.23	32.92	31.67	30.91	30.69	175.23
**13**	26.50		36.71	38.87	34.52	35.90	31.50	41.80	31.10	32.49	32.67	34.31	33.98	26.50	41.80
**14**	29.50		46.56	37.35	37.13	32.20	37.86	55.33	41.95	47.36	29.67	36.21	36.95	29.50	55.33
**15**	34.33	35.00	55.13	45.78	50.50	50.50	30.00	64.05	47.20	44.57	158.48	40.03	45.29	30.00	158.48
**16**			96.64	55.47	67.99	47.50	47.00	53.00	38.59	46.23	284.20	41.07	47.17	38.59	284.20
**17**	35.00		75.60	43.59	294.96	66.00	34.00	45.50	46.73	64.83	73.00	51.69	50.63	34.00	294.96
**18**		39.00	69.72	52.91	106.69	50.00	44.67	85.27	54.38	51.58	179.70	47.74	64.60	39.00	179.70
**19**	39.00		81.60	52.06	59.07	54.67		72.00	44.67	57.94	432.00	58.95	60.67	39.00	432.00
**20**			142.12	84.21	77.33	59.67	49.00	59.33	44.47	45.93	170.00	54.33	85.57	44.47	170.00
**21**			73.87	80.71	68.13	51.33	92.00	101.08	100.75	484.29	119.44	78.13	73.81	51.33	484.29
**22**	101.00		101.25	67.93	68.62	82.00		125.00	62.00	908.92	183.00	85.87	68.88	62.00	908.92
**23**			79.00	76.25	102.95			107.00	195.82	729.73		65.55	107.57	65.55	729.73
**24**	68.00	278.00	148.38	92.20	159.72	65.50		96.18	77.24	90.54	113.75	120.69	131.86	65.50	278.00
**25**			82.00	58.80	85.26		57.00		70.60	88.50		69.50	101.04	57.00	101.04
**26**			71.67	83.20	99.25	92.00		77.50	113.90	151.56		162.43	204.43	71.67	204.43
**27**	69.00		104.15	97.75	113.38	81.50		98.92	140.89	82.10		78.00	145.84	69.00	145.84
**28**			99.67	112.00	78.22				83.83	83.31		221.22	166.45	78.22	221.22
**29**			71.00	74.75	87.82			279.00	77.67	112.00		73.00	154.26	71.00	279.00
**30**			138.27	126.89	139.17	89.00		202.05	264.00	131.92	207.33	85.70	144.64	85.70	264.00
**31**			99.07	97.50	91.32				744.89	629.57	272.00	201.67	181.26	91.32	744.89
**32**			78.25	68.00	89.08	85.00		100.50	214.67	138.80	91.00	100.25	141.14	68.00	214.67
**33**			161.97	159.75	124.18			167.27	199.00	1138.60	115.00	140.33	139.32	115.00	1138.60
**34**			370.50	93.83	186.54			757.00	70.67	809.60		124.33	165.45	70.67	809.60
**35**			96.00	194.50	139.52			95.00	493.60	3262.25		111.92	175.24	95.00	3262.25
**36**	210.00		172.33	121.86	141.67			226.70	218.50	182.47	173.44	145.73	160.85	121.86	226.70
**37**			173.00	99.00	149.42	74.00		146.00		144.71		276.50	134.57	74.00	276.50
**38**			152.33	113.67	111.89			117.00	78.00	119.00		115.50	162.68	78.00	162.68
**39**			159.25	138.00	161.21			1055.80	196.67	148.14		92.75	150.67	92.75	1055.80
**40**					105.36			97.00		144.50		104.25	189.43	97.00	189.43
**41**			158.00	82.00	104.86					99.00		219.67	160.73	82.00	219.67
**42**			136.13	117.88	145.95	124.00		272.60	91.00	87.00		104.00	192.23	87.00	272.60
**43**			115.33	92.33	137.81	92.00		131.00	94.00	89.67			194.87	89.67	194.87
**44**					187.83				271.00	1535.50		214.75	204.67	187.83	1535.50
**45**			207.53	292.00	178.13			196.67	195.50	713.43	198.50	159.75	227.54	159.75	713.43
**46**				141.50	155.62					5087.75		183.00	195.38	141.50	5087.75
**47**			1056.60		231.43			173.00	173.00	1254.67			212.44	173.00	1254.67
**48**			191.50	249.00	371.05			1492.50	212.00	1685.00	163.00	257.33	283.50	163.00	1685.00
**49**			134.00	190.00	200.64				100.00	167.33		128.00	209.13	100.00	209.13
**50**			594.00	170.33	170.41			160.00	149.00			306.50	261.21	149.00	594.00

Additional file 5: (c) Number of satellites in TR classes in different genomic regions of *Daphnia pulex*, the euchromatic genome of *Drosophila melanogaster* and *Apis mellifera*.

unit size	Dappu v1.1 3'UTR #sat	Dappu v1.1 5'UTR #sat	Dappu v1.1 CDS #sat	Dappu v1.1 introns #sat	Dappu v1.1 intergenic #sat	DroMel-5.5 3'UTR #sat	DroMel-5.5 5'UTR #sat	DroMel-5.5 CDS #sat	DroMel-5.5 introns #sat	DroMel-5.5 intergenic #sat	ApiMel-2.0 CDS #sat	ApiMel-2.0 introns #sat	ApiMel-2.0 intergenic #sat	min #sat	max #sat
**1**	257	26	41	5614	11161	432	204	12	7254	6847	22	4367	48591	12	48591
**2**	161	38	38	5218	10613	737	382	16	6550	6615	45	4588	60487	16	60487
**3**	99	31	1998	6018	13015	297	300	2489	3084	3179	381	1858	25168	31	25168
**4**	27	4	54	1456	3077	178	93	6	2068	2091	19	1122	15143	4	15143
**5**	22	12	31	1013	2382	169	107	11	1760	1987	10	800	10354	10	10354
**6**	12	1	172	536	1621	294	177	644	3266	3732	104	598	7725	1	7725
**7**	9	6	39	430	1173	107	85	24	2010	2429	16	596	6947	6	6947
**8**	9	3	32	462	1069	77	51	18	1329	1563	9	695	7519	3	7519
**9**	6	5	350	407	1384	57	40	303	794	967	63	635	7510	5	7510
**10**	10	0	57	397	1572	36	16	3	561	670	38	694	8359	0	8359
**11**	15	4	26	274	1008	21	16	11	372	599	19	624	6889	4	6889
**12**	13	2	367	289	1076	47	27	323	593	787	52	607	7240	2	7240
**13**	2	0	21	131	615	10	2	5	152	242	3	313	3528	0	3528
**14**	4	0	25	77	341	5	7	3	133	185	3	164	2081	0	2081
**15**	3	1	212	104	434	10	1	132	105	133	25	118	1218	1	1218
**16**	0	0	11	32	127	4	1	1	56	84	5	46	764	0	764
**17**	1	0	10	29	346	3	1	2	30	36	2	48	475	0	475
**18**	0	1	130	32	175	2	3	101	47	48	10	35	433	0	433
**19**	2	0	5	35	238	3	0	1	43	47	1	21	269	0	269
**20**	0	0	16	19	116	3	1	3	15	29	1	21	226	0	226
**21**	0	0	104	28	218	3	1	50	16	14	9	15	188	0	218
**22**	1	0	8	14	66	1	0	1	10	13	1	15	132	0	132
**23**	0	0	6	8	58	0	0	4	17	44	0	11	96	0	96
**24**	1	1	120	25	185	2	0	57	21	28	4	16	133	0	185
**25**	0	0	1	5	35	0	1	0	5	6	0	4	74	0	74
**26**	0	0	6	5	52	1	0	2	10	16	0	7	183	0	183
**27**	1	0	46	12	63	2	0	24	9	20	0	2	96	0	96
**28**	0	0	3	5	36	0	0	0	6	16	0	9	91	0	91
**29**	0	0	2	4	22	0	0	1	9	3	0	5	65	0	65
**30**	0	0	49	9	59	1	0	19	15	25	6	10	91	0	91
**31**	0	0	14	4	76	0	0	0	27	14	1	3	43	0	76
**32**	0	0	4	3	61	1	0	2	3	20	1	4	44	0	61
**33**	0	0	37	8	39	0	0	26	12	30	1	3	56	0	56
**34**	0	0	4	6	57	0	0	1	3	10	0	3	47	0	57
**35**	0	0	1	4	85	0	0	1	5	4	0	13	54	0	85
**36**	1	0	27	7	46	0	0	10	4	15	9	22	67	0	67
**37**	0	0	3	3	19	1	0	1	0	7	0	2	42	0	42
**38**	0	0	3	3	45	0	0	1	1	8	0	2	44	0	45
**39**	0	0	12	4	19	0	0	10	6	22	0	4	42	0	42
**40**	0	0	0	0	14	0	0	1	0	2	0	4	46	0	46
**41**	0	0	1	1	29	0	0	0	0	1	0	3	30	0	30
**42**	0	0	15	8	44	1	0	10	1	4	0	1	31	0	44
**43**	0	0	3	3	31	1	0	1	2	3	0	0	15	0	31
**44**	0	0	0	0	6	0	0	0	1	2	0	4	30	0	30
**45**	0	0	17	3	38	0	0	3	2	7	2	4	37	0	38
**46**	0	0	0	2	8	0	0	0	0	4	0	4	32	0	32
**47**	0	0	5	0	7	0	0	1	1	3	0	0	18	0	18
**48**	0	0	12	6	20	0	0	2	1	5	1	3	18	0	20
**49**	0	0	2	1	11	0	0	0	2	6	0	1	30	0	30
**50**	0	0	2	3	27	0	0	1	4	0	0	2	43	0	43

Additional file 5: (d) Mean perfection of TRs in repeat classes in different genomic regions of *Daphnia pulex*, the euchromatic genome of *Drosophila melanogaster* and *Apis mellifera*.

unit size	Dappu v1.1 3'UTR [%]	Dappu v1.1 5'UTR [%]	Dappu v1.1 CDS [%]	Dappu v1.1 introns [%]	Dappu v1.1 intergenic [%]	DroMel-5.5 3'UTR [%]	DroMel-5.5 5'UTR [%]	DroMel-5.5 CDS [%]	DroMel-5.5 introns [%]	DroMel-5.5 intergenic [%]	ApiMel-2.0 CDS [%]	ApiMel-2.0 introns [%]	ApiMel-2.0 intergenic [%]	min [%]	max [%]
**1**	99.4	99.8	99.1	99.5	99.5	99.5	99.4	99.2	99.4	99.4	98.9	99.0	99.1	98.9	99.8
**2**	99.5	99.0	99.5	99.5	99.4	98.2	98.7	98.9	98.6	98.6	98.0	98.6	98.6	98.0	99.5
**3**	99.5	99.2	99.1	99.4	99.4	98.2	98.4	97.9	98.4	98.1	97.3	98.5	98.6	97.3	99.5
**4**	99.1	98.9	99.2	99.1	99.1	98.7	98.6	100.0	98.8	98.6	98.9	98.6	98.7	98.6	100.0
**5**	99.1	98.9	99.5	99.1	98.8	98.7	98.1	99.0	98.5	98.4	98.1	98.1	98.2	98.1	99.5
**6**	99.0	100.0	98.7	98.3	98.7	98.2	98.2	97.9	98.4	98.4	96.7	97.8	98.1	96.7	100.0
**7**	98.9	100.0	98.6	98.7	98.7	98.3	98.3	97.7	98.3	98.3	98.6	97.8	97.9	97.7	100.0
**8**	98.0	97.5	98.8	99.2	99.1	98.8	98.1	98.2	98.4	98.2	98.4	98.2	98.1	97.5	99.2
**9**	100.0	98.5	98.8	98.8	98.3	98.0	98.1	97.4	98.3	98.2	97.7	98.3	98.2	97.4	100.0
**10**	98.9		97.4	98.9	98.0	98.2	99.0	96.4	98.7	98.4	96.9	98.2	98.4	96.4	99.0
**11**	98.7	100.0	97.8	99.2	99.0	99.1	97.8	95.0	98.4	96.9	97.7	98.6	98.6	95.0	100.0
**12**	98.9	95.7	98.2	98.9	98.8	98.2	98.1	97.1	98.3	97.8	99.0	98.6	98.5	95.7	99.0
**13**	100.0		98.1	98.6	98.6	99.6	98.5	95.1	98.8	98.4	97.3	98.2	98.4	95.1	100.0
**14**	100.0		96.0	98.8	98.4	100.0	98.2	95.4	98.6	98.3	100.0	98.4	98.1	95.4	100.0
**15**	100.0	100.0	97.2	97.6	97.2	97.6	100.0	96.2	97.9	98.2	95.7	98.1	97.9	95.7	100.0
**16**			96.6	97.1	97.8	99.3	95.8	92.9	99.0	97.7	97.2	97.9	97.6	92.9	99.3
**17**	100.0		96.4	98.8	94.6	95.8	100.0	96.7	97.9	97.4	94.3	96.5	97.9	94.3	100.0
**18**		100.0	97.1	97.6	97.1	96.0	98.5	97.0	98.4	98.2	95.8	97.3	97.0	95.8	100.0
**19**	100.0		94.9	98.3	97.9	98.0		92.0	99.4	98.6	86.8	97.1	97.0	86.8	100.0
**20**			96.0	96.4	96.2	98.2	100.0	95.4	99.6	99.4	96.5	97.4	97.0	95.4	100.0
**21**			96.7	96.8	97.1	97.7	92.4	95.9	97.2	97.0	95.7	97.1	97.0	92.4	97.7
**22**	96.0		95.1	97.8	98.3	92.7		93.1	97.5	97.0	91.3	96.3	97.4	91.3	98.3
**23**			94.6	97.6	97.1			91.9	97.0	96.0		96.6	96.5	91.9	97.6
**24**	100.0	93.5	97.0	98.2	97.7	97.1		96.7	96.6	96.7	91.6	94.5	96.1	91.6	100.0
**25**			94.1	98.5	98.2		98.3		96.2	98.2		97.5	96.8	94.1	98.5
**26**			96.0	95.0	97.3	98.9		94.8	96.5	95.3		98.3	95.1	94.8	98.9
**27**	100.0		96.5	97.0	97.1	95.7		96.9	95.6	97.6		96.5	95.8	95.6	100.0
**28**			96.4	96.8	98.3				97.7	97.3		92.7	97.1	92.7	98.3
**29**			98.6	98.8	97.6			90.0	98.5	93.6		97.6	95.4	90.0	98.8
**30**			95.7	98.5	95.9	95.5		95.2	90.3	94.9	95.1	98.4	95.7	90.3	98.5
**31**			94.5	95.0	97.8				88.8	90.5	89.3	94.1	96.3	88.8	97.8
**32**			97.5	99.0	97.7	100.0		96.3	93.6	97.2	96.7	96.2	96.7	93.6	100.0
**33**			95.4	95.9	96.8			96.1	96.8	97.7	94.8	96.0	96.5	94.8	97.7
**34**			95.6	97.5	94.5			85.9	100.0	95.4		96.5	95.9	85.9	100.0
**35**			95.8	92.4	95.3			96.8	94.6	91.6		96.4	95.3	91.6	96.8
**36**	98.6		97.3	98.3	97.2			93.9	93.7	95.5	95.4	94.6	96.1	93.7	98.6
**37**			94.7	99.1	98.0	100.0		93.3		94.6		90.5	96.6	90.5	100.0
**38**			92.5	98.8	97.5			97.4	100.0	97.0		97.7	96.7	92.5	100.0
**39**			97.0	93.7	96.6			96.0	95.8	97.5		98.8	97.3	93.7	98.8
**40**					97.8			98.0		95.9		99.3	95.2	95.2	99.3
**41**			92.2	100.0	98.4					98.0		96.9	97.4	92.2	100.0
**42**			97.3	96.5	97.6	94.5		97.1	98.9	100.0		97.1	96.5	94.5	100.0
**43**			96.3	99.7	97.4	100.0		95.5	100.0	100.0			97.3	95.5	100.0
**44**					94.6				92.0	93.5		93.0	96.2	92.0	96.2
**45**			95.1	94.6	97.8			96.0	95.3	97.3	98.1	96.1	96.1	94.6	98.1
**46**				97.9	96.3					89.4		95.1	96.3	89.4	97.9
**47**			90.8		93.4			92.4	92.4	90.9			96.2	90.8	96.2
**48**			97.2	94.7	94.1			97.1	96.2	94.9	97.0	93.2	95.2	93.2	97.2
**49**			96.5	96.8	95.2				100.0	95.1		98.4	96.5	95.1	100.0
**50**			89.7	98.6	97.0			94.1	95.6			98.4	95.3	89.7	98.6
