# Supplementary material for: Genome-wide analysis of tandem repeats in Daphnia pulex - a comparative approach
Source: BMC Genomics. 2010 Apr 30;11:277. doi: 10.1186/1471-2164-11-277 (PMC3152781; doi:10.1186/1471-2164-11-277)
Supplement: Additional file 7 — Genomic density, mean lengths, number of satellites and mean perfection of individual TR motifs in different genomic regions of Daphnia pulex, the euchromatic genome of Drosophila melanogaster and Apis mellifera. Only repeats in the unit size range 1-50 bp are shown that have a minimum density of 50 bp/Mbp in one genomic region of the three genomes. The second column in the table assists detecting motif pairs that differ only by the reverse complement. An A stands for the normal form and B for the reverse complement of motif A. "-" indicates palindromic motifs for which the unit is identical to its reverse complement (e.g. AT). If only one of the two motifs is present, the letters a and b are used. Thus "a" indicates that only the normal form is present, whereas "b" indicates that only the reverse complement of the normal form was found. [file 1471-2164-11-277-S7.PDF]

**Additional file 7:** (a) Genomic densities in different genomic regions of *Daphnia pulex* (Dappu), *Drosophila melanogaster* (DroMel) and *Apis mellifera* (ApiMel). A=normal B=reverse complement of repeat motif A; "-" denotes palindromic motifs; a and b are used of only only the normal form (a) OR its reverse complement (b) were found.

| unit size [bp] | motif form | unit | Dappu v1.1 3UTR bp/Mbp | Dappu v1.1 5UTR bp/Mbp | Dappu v1.1 CDS bp/Mbp | Dappu v1.1 introns bp/Mbp | Dappu v1.1 intergenic bp/Mbp | DroMel-5.5 3UTR bp/Mbp | DroMel-5.5 5UTR bp/Mbp | DroMel-5.5 CDS bp/Mbp | DroMel-5.5 introns bp/Mbp | DroMel-5.5 intergenic bp/Mbp | ApiMel-2.0 CDS bp/Mbp | ApiMel-2.0 introns bp/Mbp | ApiMel-2.0 intergenic bp/Mbp | min bp/Mbp | max bp/Mbp |
|---|---|---|---|---|---|---|---|---|---|---|---|---|---|---|---|---|---|
| 1 | A | A | 866.73 | 333.07 | 11.46 | 799.57 | 635.80 | 993.48 | 834.60 | 4.92 | 1119.99 | 800.70 | 106.21 | 1966.65 | 2069.87 | 4.92 | 2069.87 |
| 1 | B | T | 1163.64 | 266.46 | 7.33 | 1284.48 | 647.18 | 565.78 | 308.00 | 3.91 | 994.52 | 806.02 | 18.74 | 2792.61 | 2055.86 | 3.91 | 2792.61 |
| 1 | A | C | 392.30 | 22.75 | 4.83 | 498.42 | 295.83 | 76.52 | 16.65 | 0.00 | 110.57 | 85.22 | 5.08 | 27.13 | 38.34 | 0.00 | 498.42 |
| 1 | B | G | 144.03 | 27.62 | 3.47 | 464.79 | 299.47 | 102.42 | 0.00 | 0.00 | 107.63 | 98.21 | 5.08 | 27.61 | 40.47 | 0.00 | 464.79 |
| 2 | A | AC | 503.48 | 180.34 | 7.36 | 576.42 | 446.85 | 1076.86 | 678.25 | 0.70 | 703.07 | 612.09 | 5.47 | 160.77 | 167.79 | 0.70 | 1076.86 |
| 2 | B | GT | 425.79 | 437.05 | 1.99 | 768.48 | 440.71 | 180.74 | 775.61 | 5.05 | 767.16 | 644.75 | 0.00 | 167.83 | 158.27 | 0.00 | 775.61 |
| 2 | A | AG | 182.57 | 84.48 | 6.31 | 776.33 | 506.09 | 154.26 | 450.60 | 1.93 | 155.01 | 132.12 | 20.30 | 1353.26 | 1606.35 | 1.93 | 1606.35 |
| 2 | B | CT | 500.33 | 432.18 | 8.77 | 940.65 | 518.59 | 70.63 | 141.51 | 0.61 | 251.81 | 155.40 | 435.76 | 1401.68 | 1542.87 | 0.61 | 1542.87 |
| 2 | - | AT | 220.47 | 45.50 | 1.17 | 208.75 | 127.59 | 2663.58 | 701.05 | 7.38 | 886.65 | 718.69 | 8.59 | 4069.04 | 3866.05 | 1.17 | 4069.04 |
| 3 | A | AAC | 157.93 | 63.36 | 328.02 | 261.35 | 188.97 | 285.46 | 542.89 | 265.87 | 135.52 | 80.28 | 281.92 | 19.63 | 45.66 | 19.63 | 542.89 |
| 3 | B | GTT | 70.75 | 105.61 | 13.68 | 389.97 | 198.37 | 13.24 | 91.93 | 1.05 | 115.83 | 101.73 | 59.74 | 33.69 | 48.59 | 1.05 | 389.97 |
| 3 | A | AAG | 160.45 | 118.61 | 259.00 | 641.87 | 473.49 | 5.40 | 54.29 | 30.30 | 14.52 | 11.81 | 807.09 | 285.16 | 360.74 | 5.40 | 807.09 |
| 3 | B | CTT | 120.03 | 0.00 | 29.19 | 814.34 | 496.04 | 3.68 | 20.63 | 1.84 | 21.67 | 12.00 | 56.62 | 345.60 | 367.32 | 0.00 | 814.34 |
| 3 | A | AAT | 128.24 | 26.00 | 6.12 | 139.18 | 109.37 | 150.09 | 62.25 | 33.41 | 140.47 | 213.85 | 365.08 | 1014.35 | 596.03 | 6.12 | 1014.35 |
| 3 | B | ATT | 216.05 | 24.37 | 2.88 | 217.96 | 122.18 | 239.36 | 56.10 | 0.92 | 165.63 | 192.06 | 89.42 | 644.38 | 561.82 | 0.92 | 644.38 |
| 3 | A | ACC | 34.75 | 55.25 | 94.19 | 92.61 | 67.39 | 90.49 | 77.09 | 122.73 | 35.38 | 18.65 | 351.81 | 27.61 | 44.48 | 18.65 | 351.81 |
| 3 | B | GGT | 0.00 | 30.87 | 41.78 | 96.54 | 60.35 | 5.40 | 24.61 | 50.45 | 24.43 | 24.99 | 466.22 | 35.96 | 44.41 | 0.00 | 466.22 |
| 3 | A | ACG | 11.38 | 0.00 | 93.92 | 93.01 | 76.17 | 12.75 | 39.09 | 45.97 | 14.15 | 11.40 | 158.53 | 59.46 | 86.18 | 0.00 | 158.53 |
| 3 | B | CGT | 0.00 | 24.37 | 37.02 | 106.15 | 69.56 | 12.02 | 22.08 | 8.47 | 24.91 | 13.89 | 71.46 | 57.25 | 86.92 | 0.00 | 106.15 |
| 3 | A | ACT | 52.43 | 48.75 | 7.05 | 74.25 | 42.31 | 29.92 | 5.43 | 1.84 | 26.88 | 12.90 | 747.74 | 41.60 | 37.77 | 1.84 | 747.74 |
| 3 | B | AGT | 0.00 | 0.00 | 2.26 | 64.02 | 37.95 | 6.38 | 0.00 | 2.99 | 17.63 | 12.18 | 733.29 | 107.39 | 37.36 | 0.00 | 733.29 |
| 3 | A | AGC | 21.48 | 191.72 | 340.68 | 324.05 | 233.16 | 442.17 | 993.13 | 1658.93 | 262.27 | 217.25 | 219.05 | 7.36 | 27.05 | 7.36 | 1658.93 |
| 3 | B | CTG | 70.75 | 126.73 | 74.74 | 335.09 | 249.51 | 40.96 | 46.69 | 26.87 | 174.35 | 157.82 | 173.76 | 7.67 | 19.23 | 7.67 | 335.09 |
| 3 | A | AGG | 0.00 | 0.00 | 41.97 | 50.07 | 34.68 | 115.51 | 111.47 | 166.77 | 31.83 | 21.51 | 192.89 | 223.30 | 310.11 | 0.00 | 310.11 |
| 3 | B | CCT | 22.11 | 37.37 | 21.59 | 56.57 | 33.17 | 23.30 | 14.48 | 21.56 | 29.24 | 24.90 | 213.19 | 239.26 | 315.61 | 14.48 | 315.61 |
| 3 | A | ATC | 50.54 | 87.73 | 24.55 | 70.49 | 45.75 | 45.62 | 66.96 | 46.68 | 73.95 | 65.51 | 102.30 | 29.89 | 41.02 | 24.55 | 102.30 |
| 3 | B | ATG | 23.37 | 0.00 | 19.52 | 72.59 | 43.04 | 106.19 | 27.51 | 29.46 | 94.49 | 62.51 | 986.71 | 59.89 | 40.81 | 0.00 | 986.71 |
| 3 | A | CCG | 12.01 | 0.00 | 59.39 | 36.15 | 40.52 | 3.68 | 30.76 | 27.53 | 7.45 | 13.34 | 53.10 | 19.09 | 24.06 | 0.00 | 59.39 |
| 3 | B | CGG | 0.00 | 0.00 | 73.14 | 46.51 | 38.54 | 29.43 | 17.73 | 54.67 | 10.11 | 13.40 | 20.69 | 16.51 | 27.54 | 0.00 | 73.14 |
| 4 | A | AAAC | 40.43 | 53.62 | 1.60 | 32.62 | 32.39 | 87.80 | 96.27 | 0.00 | 34.75 | 31.12 | 0.00 | 5.28 | 6.04 | 0.00 | 96.27 |
| 4 | B | GTTT | 20.22 | 0.00 | 2.42 | 79.66 | 34.70 | 11.53 | 32.94 | 0.00 | 35.56 | 22.28 | 0.00 | 3.32 | 8.39 | 0.00 | 79.66 |
| 4 | A | AAAG | 13.90 | 0.00 | 0.62 | 108.69 | 69.75 | 33.35 | 15.20 | 0.00 | 6.49 | 4.66 | 48.03 | 222.51 | 257.91 | 0.00 | 257.91 |
| 4 | B | CTTT | 48.01 | 35.75 | 3.43 | 140.33 | 67.64 | 0.00 | 0.00 | 0.00 | 7.40 | 3.89 | 8.59 | 276.38 | 266.20 | 0.00 | 276.38 |
| 4 | A | AAAT | 95.39 | 0.00 | 0.00 | 36.91 | 31.62 | 51.75 | 83.97 | 0.00 | 45.84 | 67.47 | 0.00 | 172.49 | 194.13 | 0.00 | 194.13 |
| 4 | B | ATTT | 34.75 | 0.00 | 0.62 | 144.22 | 38.93 | 73.82 | 14.84 | 0.00 | 67.34 | 87.25 | 7.03 | 234.66 | 194.61 | 0.00 | 234.66 |
| 4 | a | AACC | 10.10 | 0.00 | 0.00 | 6.94 | 6.91 | 107.91 | 77.45 | 0.88 | 33.09 | 11.87 | 0.00 | 2.34 | 4.21 | 0.00 | 107.91 |
| 4 | A | AAGG | 0.00 | 0.00 | 4.40 | 10.29 | 12.03 | 3.92 | 0.00 | 0.00 | 3.60 | 2.15 | 19.52 | 82.90 | 84.94 | 0.00 | 84.94 |
| 4 | B | CCTT | 0.00 | 0.00 | 0.70 | 24.49 | 8.36 | 0.00 | 0.00 | 0.00 | 5.83 | 2.12 | 0.00 | 54.49 | 98.48 | 0.00 | 98.48 |
| 4 | - | AATT | 0.00 | 0.00 | 0.00 | 17.51 | 5.19 | 18.15 | 15.20 | 0.00 | 17.83 | 14.38 | 0.00 | 54.31 | 55.13 | 0.00 | 55.13 |
| 4 | A | ACAT | 0.00 | 0.00 | 0.62 | 13.01 | 44.89 | 118.70 | 87.95 | 0.00 | 85.20 | 67.48 | 0.00 | 40.56 | 31.61 | 0.00 | 118.70 |
| 4 | B | ATGT | 0.00 | 0.00 | 0.00 | 15.64 | 39.39 | 66.22 | 12.67 | 0.00 | 68.79 | 60.63 | 0.00 | 26.94 | 31.02 | 0.00 | 68.79 |
| 4 | a | AGCC | 0.00 | 26.00 | 4.17 | 22.62 | 20.87 | 33.35 | 57.91 | 0.83 | 32.02 | 20.66 | 0.00 | 16.20 | 9.38 | 0.00 | 57.91 |
| 4 | A | AGGG | 0.00 | 0.00 | 0.00 | 6.50 | 4.89 | 0.00 | 0.00 | 0.00 | 1.01 | 0.40 | 0.00 | 49.34 | 56.43 | 0.00 | 56.43 |
| 4 | B | CCCT | 0.00 | 0.00 | 0.00 | 5.22 | 3.23 | 0.00 | 0.00 | 0.00 | 1.21 | 0.81 | 6.25 | 40.87 | 55.36 | 0.00 | 55.36 |
| 4 | a | ATCC | 20.22 | 0.00 | 2.96 | 68.15 | 26.01 | 9.56 | 0.00 | 0.00 | 26.57 | 26.43 | 0.00 | 12.03 | 15.95 | 0.00 | 68.15 |
| 5 | A | AAAAC | 108.02 | 0.00 | 0.74 | 41.94 | 31.78 | 134.88 | 27.87 | 0.00 | 15.32 | 10.44 | 0.00 | 3.92 | 5.18 | 0.00 | 134.88 |
| 5 | B | GTTTT | 34.75 | 27.62 | 1.32 | 57.35 | 31.77 | 0.00 | 7.96 | 0.00 | 17.96 | 6.94 | 0.00 | 5.34 | 4.70 | 0.00 | 57.35 |
| 5 | A | AAAAG | 22.74 | 27.62 | 1.71 | 67.91 | 40.56 | 29.18 | 6.51 | 0.00 | 11.59 | 10.38 | 16.40 | 115.18 | 118.58 | 0.00 | 118.58 |
| 5 | B | CTTTT | 27.17 | 40.62 | 0.00 | 85.42 | 37.86 | 5.89 | 0.00 | 0.00 | 7.03 | 5.76 | 19.91 | 135.49 | 119.54 | 0.00 | 135.49 |
| 5 | A | AAAAT | 32.85 | 69.86 | 0.00 | 39.16 | 56.61 | 33.11 | 38.00 | 0.00 | 23.07 | 20.63 | 0.00 | 75.41 | 85.14 | 0.00 | 85.14 |
| 5 | B | ATTTT | 25.90 | 27.62 | 0.00 | 132.33 | 50.53 | 16.43 | 0.00 | 0.00 | 20.31 | 19.90 | 0.00 | 114.19 | 92.46 | 0.00 | 132.33 |
| 5 | A | AACCT | 0.00 | 0.00 | 0.00 | 10.03 | 59.76 | 0.00 | 0.00 | 0.00 | 0.00 | 0.00 | 0.00 | 15.89 | 18.00 | 0.00 | 59.76 |
| 5 | B | AGGTT | 0.00 | 0.00 | 0.00 | 12.16 | 52.52 | 0.00 | 0.00 | 0.00 | 0.00 | 0.00 | 0.00 | 35.22 | 23.91 | 0.00 | 52.52 |
| 5 | A | AAGAT | 0.00 | 0.00 | 0.00 | 4.94 | 4.78 | 0.00 | 0.00 | 0.00 | 0.35 | 178.52 | 0.00 | 1.66 | 3.59 | 0.00 | 178.52 |
| 5 | B | ATCTT | 0.00 | 0.00 | 0.00 | 4.07 | 6.04 | 0.00 | 0.00 | 0.00 | 1.87 | 59.34 | 0.00 | 2.34 | 2.72 | 0.00 | 59.34 |
| 5 | A | AATAG | 0.00 | 0.00 | 0.00 | 3.79 | 4.10 | 0.00 | 6.51 | 0.00 | 2.72 | 64.37 | 8.98 | 5.03 | 4.02 | 0.00 | 64.37 |
| 5 | B | ATTCT | 0.00 | 0.00 | 0.00 | 3.76 | 3.50 | 0.00 | 6.51 | 0.00 | 2.48 | 143.05 | 0.00 | 6.50 | 3.53 | 0.00 | 143.05 |
| 5 | A | AATAT | 0.00 | 0.00 | 0.00 | 2.50 | 3.32 | 6.38 | 0.00 | 0.00 | 17.92 | 125.16 | 0.00 | 172.19 | 177.61 | 0.00 | 177.61 |
| 5 | B | ATATT | 11.38 | 0.00 | 0.00 | 10.94 | 2.71 | 18.39 | 26.06 | 0.00 | 25.15 | 68.09 | 13.28 | 163.29 | 163.29 | 0.00 | 163.29 |
| 5 | A | AATCG | 0.00 | 0.00 | 0.00 | 1.22 | 0.00 | 24.77 | 59.72 | 0.00 | 22.56 | 15.35 | 0.00 | 11.78 | 8.57 | 0.00 | 59.72 |
| 5 | B | ATTCG | 0.00 | 0.00 | 0.00 | 0.00 | 0.18 | 0.00 | 77.09 | 0.75 | 16.43 | 14.79 | 0.00 | 9.51 | 9.44 | 0.00 | 77.09 |
| 5 | A | ATCCG | 0.00 | 0.00 | 0.66 | 0.00 | 0.62 | 10.79 | 61.53 | 0.79 | 10.47 | 13.70 | 0.00 | 2.27 | 1.35 | 0.00 | 61.53 |
| 5 | B | ATCGG | 0.00 | 0.00 | 0.00 | 0.00 | 0.19 | 10.30 | 63.34 | 0.75 | 14.69 | 9.81 | 0.00 | 0.00 | 1.49 | 0.00 | 63.34 |
| 6 | a | AAAAAC | 46.75 | 29.25 | 0.90 | 9.38 | 12.84 | 75.53 | 0.00 | 0.00 | 11.13 | 10.95 | 0.00 | 0.00 | 2.00 | 0.00 | 75.53 |
| 6 | A | AAAAAG | 27.17 | 0.00 | 4.01 | 43.16 | 37.87 | 5.40 | 0.00 | 1.58 | 4.06 | 3.08 | 20.69 | 81.61 | 89.08 | 0.00 | 89.08 |
| 6 | B | CTTTTT | 25.90 | 0.00 | 0.00 | 69.27 | 34.57 | 5.15 | 0.00 | 0.00 | 2.58 | 1.96 | 0.00 | 81.00 | 81.00 | 0.00 | 81.00 |
| 6 | b | ATTTTT | 11.38 | 0.00 | 0.00 | 60.15 | 21.19 | 5.64 | 0.00 | 0.00 | 17.11 | 11.87 | 0.00 | 49.82 | 42.94 | 0.00 | 60.15 |
| 6 | a | AAAAGC | 0.00 | 0.00 | 1.13 | 0.00 | 1.91 | 22.32 | 50.67 | 0.00 | 9.96 | 6.30 | 0.00 | 0.00 | 0.19 | 0.00 | 50.67 |
| 6 | a | AAATAC | 0.00 | 0.00 | 0.00 | 0.00 | 0.70 | 6.62 | 6.51 | 0.00 | 4.91 | 4.36 | 82.78 | 0.00 | 1.04 | 0.00 | 82.78 |
| 6 | A | AAATAT | 0.00 | 0.00 | 0.00 | 1.53 | 1.18 | 20.11 | 6.51 | 0.00 | 8.74 | 7.69 | 0.00 | 48.18 | 51.47 | 0.00 | 51.47 |
| 6 | B | ATATTT | 0.00 | 0.00 | 0.00 | 3.93 | 0.85 | 4.41 | 7.96 | 0.00 | 20.56 | 9.89 | 0.00 | 123.15 | 46.70 | 0.00 | 123.15 |
| 6 | a | AACAAT | 0.00 | 0.00 | 0.00 | 0.00 | 0.64 | 0.00 | 0.00 | 7.77 | 4.45 | 3.08 | 169.85 | 7.55 | 4.56 | 0.00 | 169.85 |
| 6 | a | AACAGC | 0.00 | 0.00 | 15.12 | 6.00 | 2.17 | 96.14 | 119.07 | 150.56 | 22.02 | 16.48 | 37.48 | 0.00 | 4.62 | 0.00 | 150.56 |
| 6 | A | AACCCC | 0.00 | 0.00 | 0.00 | 0.00 | 73.30 | 0.00 | 0.00 | 0.00 | 0.00 | 0.74 | 0.00 | 0.00 | 0.00 | 0.00 | 73.30 |
| 6 | B | GGGGTT | 0.00 | 0.00 | 0.00 | 0.60 | 72.59 | 0.00 | 0.00 | 0.00 | 0.35 | 1.12 | 0.00 | 0.00 | 0.00 | 0.00 | 72.59 |
| 6 | a | AAGAGT | 0.00 | 0.00 | 0.00 | 0.00 | 0.00 | 0.00 | 0.00 | 0.00 | 0.00 | 0.00 | 187.42 | 0.00 | 0.09 | 0.00 | 187.42 |
| 6 | a | AAGCAG | 0.00 | 0.00 | 0.74 | 0.78 | 0.66 | 15.21 | 10.86 | 1.67 | 2.08 | 1.44 | 1480.64 | 46.26 | 2.47 | 0.00 | 1480.64 |
| 6 | a | AAGGCG | 0.00 | 0.00 | 0.00 | 1.35 | 0.00 | 0.00 | 0.00 | 0.00 | 0.00 | 0.53 | 106.21 | 0.00 | 0.22 | 0.00 | 106.21 |
| 6 | A | AATAGT | 0.00 | 0.00 | 0.00 | 0.65 | 0.75 | 4.41 | 0.00 | 0.00 | 0.00 | 1.30 | 67.55 | 9.51 | 2.62 | 0.00 | 67.55 |
| 6 | B | ACTATT | 0.00 | 0.00 | 0.00 | 0.00 | 0.00 | 7.11 | 0.00 | 0.00 | 0.89 | 1.61 | 77.31 | 2.94 | 3.33 | 0.00 | 77.31 |
| 6 | a | AATCGG | 0.00 | 0.00 | 0.00 | 0.00 | 0.00 | 0.00 | 64.42 | 0.00 | 6.04 | 6.33 | 0.00 | 0.00 | 0.10 | 0.00 | 64.42 |
| 6 | a | AATGAC | 0.00 | 0.00 | 0.00 | 0.00 | 0.27 | 0.00 | 7.60 | 0.00 | 0.50 | 0.52 | 58.57 | 0.00 | 0.46 | 0.00 | 58.57 |
| 6 | a | AATGAT | 0.00 | 0.00 | 0.00 | 0.88 | 0.00 | 4.41 | 0.00 | 1.62 | 3.37 | 2.27 | 184.30 | 15.65 | 2.29 | 0.00 | 184.30 |
| 6 | A | ACAGAT | 0.00 | 0.00 | 0.00 | 0.00 | 0.00 | 33.11 | 74.92 | 2.99 | 91.90 | 63.31 | 0.00 | 1.22 | 0.09 | 0.00 | 91.90 |
| 6 | B | ATCTGT | 0.00 | 0.00 | 0.00 | 0.00 | 0.67 | 52.24 | 0.00 | 1.36 | 68.77 | 49.25 | 0.00 | 0.00 | 0.11 | 0.00 | 68.77 |
| 6 | b | CGGTCT | 0.00 | 0.00 | 0.00 | 0.00 | 0.27 | 0.00 | 0.00 | 0.00 | 0.00 | 0.59 | 121.43 | 0.00 | 0.41 | 0.00 | 121.43 |
| 6 | b | ATCGTG | 0.00 | 0.00 | 0.00 | 0.00 | 0.26 | 0.00 | 0.00 | 2.11 | 0.35 | 0.00 | 146.03 | 0.00 | 1.61 | 0.00 | 146.03 |
| 6 | a | ATCCGC | 0.00 | 0.00 | 0.00 | 0.00 | 0.00 | 11.77 | 50.67 | 15.06 | 10.00 | 11.59 | 0.00 | 0.00 | 0.00 | 0.00 | 50.67 |
| 7 | a | AAAAAAC | 12.01 | 0.00 | 0.74 | 16.49 | 11.73 | 51.01 | 16.65 | 0.00 | 10.33 | 8.82 | 0.00 | 2.14 | 2.48 | 0.00 | 51.01 |
| 7 | A | AAAAAAG | 0.00 | 0.00 | 0.00 | 30.38 | 20.85 | 0.00 | 18.10 | 0.00 | 2.85 | 0.68 | 16.01 | 73.09 | 67.00 | 0.00 | 73.09 |
| 7 | B | CTTTTTT | 0.00 | 0.00 | 0.00 | 31.56 | 15.38 | 0.00 | 0.00 | 0.00 | 2.08 | 0.00 | 10.15 | 96.90 | 78.93 | 0.00 | 96.90 |
| 7 | A | AATATAT | 0.00 | 0.00 | 0.00 | 2.54 | 0.85 | 0.00 | 0.00 | 0.00 | 14.23 | 42.61 | 0.00 | 100.14 | 97.46 | 0.00 | 100.14 |
| 7 | B | ATATATT | 0.00 | 0.00 | 0.00 | 3.49 | 1.44 | 9.32 | 0.00 | 0.00 | 10.93 | 31.24 | 0.00 | 88.67 | 97.80 | 0.00 | 97.80 |
| 8 | A | AAAAAAAG | 0.00 | 0.00 | 0.00 | 15.27 | 10.58 | 9.81 | 14.48 | 0.00 | 5.22 | 1.43 | 8.98 | 69.83 | 55.81 | 0.00 | 69.83 |
| 8 | B | CTTTTTTT | 0.00 | 0.00 | 0.00 | 17.07 | 12.54 | 0.00 | 0.00 | 0.00 | 0.00 | 1.12 | 0.00 | 70.13 | 51.98 | 0.00 | 70.13 |
| 8 | a | ACATATAT | 0.00 | 0.00 | 0.00 | 0.00 | 0.77 | 42.18 | 7.60 | 0.00 | 7.14 | 88.27 | 0.00 | 18.35 | 17.50 | 0.00 | 88.27 |
| 9 | b | CTTTTTTTT | 0.00 | 64.98 | 0.00 | 8.16 | 4.47 | 0.00 | 0.00 | 0.00 | 0.00 | 0.44 | 0.00 | 67.63 | 39.06 | 0.00 | 67.63 |
| 9 | a | AACAGCAGC | 0.00 | 0.00 | 4.40 | 1.82 | 1.43 | 16.19 | 44.88 | 86.63 | 8.02 | 8.46 | 19.52 | 0.00 | 3.67 | 0.00 | 86.63 |
| 9 | a | AATAATCAT | 0.00 | 0.00 | 0.00 | 0.00 | 0.27 | 0.00 | 0.00 | 0.00 | 59.69 | 0.43 | 0.00 | 1.54 | 0.45 | 0.00 | 59.69 |
| 9 | b | ACTACTATT | 0.00 | 0.00 | 0.00 | 0.00 | 0.23 | 0.00 | 0.00 | 0.00 | 0.00 | 0.00 | 101.13 | 0.00 | 1.50 | 0.00 | 101.13 |
| 9 | a | AATATATAT | 0.00 | 0.00 | 0.00 | 1.12 | 1.00 | 0.00 | 0.00 | 0.00 | 5.75 | 3.12 | 0.00 | 56.39 | 40.40 | 0.00 | 56.39 |
| 10 | a | AAAGCTCGAG | 0.00 | 0.00 | 0.00 | 0.00 | 0.00 | 0.00 | 0.00 | 0.00 | 0.00 | 0.00 | 158.14 | 0.00 | 0.00 | 0.00 | 158.14 |

| | | | | | | | | | | | | | | | | | |
|---|---|---|---|---|---|---|---|---|---|---|---|---|---|---|---|---|---|
| 10 | b | AAGGTTCGCC | 0.00 | 0.00 | 105.65 | 0.00 | 4.00 | 0.00 | 0.00 | 0.00 | 0.00 | 0.00 | 0.00 | 0.00 | 0.00 | 0.00 | 105.65 |
| 10 | b | ACTTGGATGG | 0.00 | 0.00 | 0.00 | 0.00 | 0.00 | 0.00 | 0.00 | 0.00 | 0.00 | 0.00 | 354.93 | 14.67 | 0.73 | 0.00 | 354.93 |
| 10 | b | ATCGTGGTCC | 0.00 | 0.00 | 0.00 | 0.00 | 0.00 | 0.00 | 0.00 | 0.00 | 0.00 | 0.00 | 303.00 | 0.00 | 0.00 | 0.00 | 303.00 |
| 10 | a | ACCATGGACT | 0.00 | 0.00 | 0.00 | 0.00 | 0.00 | 0.00 | 0.00 | 0.00 | 0.00 | 0.00 | 140.96 | 0.00 | 0.00 | 0.00 | 140.96 |
| 10 | b | ACGGTGGACT | 0.00 | 0.00 | 0.00 | 0.00 | 0.00 | 0.00 | 0.00 | 0.00 | 0.00 | 0.00 | 192.11 | 0.00 | 0.69 | 0.00 | 192.11 |
| 10 | a | ACGATGGACT | 0.00 | 0.00 | 0.00 | 0.00 | 0.00 | 0.00 | 0.00 | 0.00 | 0.00 | 0.00 | 78.09 | 37.43 | 5.18 | 0.00 | 78.09 |
| 10 | b | CGTGCTCTGG | 0.00 | 0.00 | 40.26 | 0.00 | 81.72 | 0.00 | 0.00 | 0.00 | 0.00 | 0.00 | 0.00 | 0.00 | 0.00 | 0.00 | 81.72 |
| 10 | A | ACGCCAGTGC | 0.00 | 0.00 | 74.82 | 0.00 | 14.35 | 0.00 | 0.00 | 0.00 | 0.00 | 0.00 | 0.00 | 0.00 | 0.00 | 0.00 | 74.82 |
| 10 | B | ACTGGCGTGC | 0.00 | 0.00 | 0.00 | 0.00 | 62.40 | 0.00 | 0.00 | 0.00 | 0.00 | 0.00 | 0.00 | 0.00 | 0.00 | 0.00 | 62.40 |
| 11 | a | AAACAGCACTC | 0.00 | 71.48 | 0.00 | 0.00 | 0.00 | 0.00 | 0.00 | 0.00 | 0.00 | 0.00 | 0.00 | 0.00 | 0.00 | 0.00 | 71.48 |
| 11 | A | ACCAGTACGGG | 0.00 | 0.00 | 0.00 | 0.00 | 0.00 | 0.00 | 0.00 | 0.00 | 4.43 | 2471.11 | 0.00 | 0.00 | 0.00 | 0.00 | 2471.11 |
| 11 | B | ACTGGTCCCGT | 0.00 | 0.00 | 0.00 | 0.00 | 0.00 | 0.00 | 0.00 | 114.87 | 29.55 | 2518.69 | 0.00 | 0.00 | 0.00 | 0.00 | 2518.69 |
| 12 | a | AACAGCAGCAGC | 0.00 | 0.00 | 3.58 | 3.66 | 0.58 | 8.09 | 0.00 | 61.82 | 5.59 | 3.08 | 12.49 | 0.00 | 0.68 | 0.00 | 61.82 |
| 12 | b | ATGCCTGGTTGC | 63.17 | 0.00 | 0.00 | 0.00 | 0.00 | 0.00 | 0.00 | 0.00 | 0.00 | 0.00 | 0.00 | 0.00 | 0.00 | 0.00 | 63.17 |
| 12 | a | AATGATGATGAT | 0.00 | 0.00 | 0.00 | 0.00 | 0.00 | 6.87 | 0.00 | 0.00 | 1.08 | 0.00 | 54.27 | 0.00 | 0.21 | 0.00 | 54.27 |
| 12 | A | ACCGAGTACGGG | 0.00 | 0.00 | 0.00 | 0.00 | 0.00 | 0.00 | 0.00 | 0.00 | 11.84 | 678.35 | 0.00 | 0.00 | 0.00 | 0.00 | 678.35 |
| 12 | B | ACTCGGTCCCGT | 0.00 | 0.00 | 0.00 | 0.00 | 0.00 | 0.00 | 0.00 | 0.00 | 22.06 | 1006.27 | 0.00 | 0.00 | 0.00 | 0.00 | 1006.27 |
| 12 | a | AGCAGCCTATCC | 0.00 | 94.23 | 0.00 | 0.00 | 0.00 | 0.00 | 0.00 | 0.00 | 0.00 | 0.00 | 0.00 | 0.00 | 0.00 | 0.00 | 94.23 |
| 15 | a | AAAAATAGTGGACCG | 0.00 | 0.00 | 0.00 | 0.00 | 0.00 | 0.00 | 0.00 | 0.00 | 0.00 | 0.00 | 194.84 | 0.00 | 0.00 | 0.00 | 194.84 |
| 15 | a | AAAAGTCTACTGATT | 0.00 | 56.87 | 0.00 | 0.00 | 0.00 | 0.00 | 0.00 | 0.00 | 0.00 | 0.00 | 0.00 | 0.00 | 0.00 | 0.00 | 56.87 |
| 15 | b | AACTTAGTTTATTTG | 0.00 | 0.00 | 0.00 | 0.00 | 0.00 | 0.00 | 0.00 | 0.00 | 0.00 | 0.00 | 569.69 | 7.67 | 0.00 | 0.00 | 569.69 |
| 15 | b | AACTTAGTTCATTTG | 0.00 | 0.00 | 0.00 | 0.00 | 0.00 | 0.00 | 0.00 | 0.00 | 0.00 | 0.00 | 57.01 | 0.00 | 0.00 | 0.00 | 57.01 |
| 15 | b | ACATTTGCTCCGCCG | 0.00 | 0.00 | 0.00 | 0.00 | 0.00 | 0.00 | 0.00 | 0.00 | 0.00 | 0.00 | 55.06 | 0.00 | 0.00 | 0.00 | 55.06 |
| 15 | a | AACCCAGCCTGACCC | 0.00 | 0.00 | 0.00 | 0.00 | 0.00 | 0.00 | 0.00 | 0.00 | 0.00 | 0.00 | 160.09 | 0.00 | 0.00 | 0.00 | 160.09 |
| 15 | a | AAGTCACGGAGGACG | 0.00 | 0.00 | 0.00 | 0.00 | 0.00 | 0.00 | 0.00 | 0.00 | 0.00 | 0.00 | 137.05 | 0.00 | 0.00 | 0.00 | 137.05 |
| 15 | b | ACGATGGTGTCGATG | 0.00 | 0.00 | 0.00 | 0.00 | 0.00 | 0.00 | 0.00 | 0.00 | 0.00 | 0.00 | 83.95 | 0.00 | 0.00 | 0.00 | 83.95 |
| 16 | a | AAAAATCTCAAAGACC | 0.00 | 0.00 | 0.00 | 0.00 | 0.00 | 0.00 | 0.00 | 0.00 | 0.00 | 0.00 | 228.81 | 0.00 | 0.00 | 0.00 | 228.81 |
| 16 | a | AAATTCGAAGGCATCC | 0.00 | 0.00 | 0.00 | 0.00 | 0.00 | 0.00 | 0.00 | 0.00 | 0.00 | 0.00 | 284.26 | 0.00 | 0.00 | 0.00 | 284.26 |
| 17 | A | AAAAGTAGAACTTTTCT | 0.00 | 0.00 | 0.00 | 0.00 | 223.16 | 0.00 | 0.00 | 0.00 | 0.00 | 0.00 | 0.00 | 0.00 | 0.00 | 0.00 | 223.16 |
| 17 | B | AAAAGTTCTACTTTTAG | 0.00 | 0.00 | 0.00 | 0.00 | 122.27 | 0.00 | 0.00 | 0.00 | 0.00 | 0.00 | 0.00 | 0.00 | 0.00 | 0.00 | 122.27 |
| 17 | A | AAAAGTTCAACTTTATG | 0.00 | 0.00 | 0.00 | 0.00 | 173.70 | 0.00 | 0.00 | 0.00 | 0.00 | 0.00 | 0.00 | 0.00 | 0.00 | 0.00 | 173.70 |
| 17 | B | AAAGTTGAACTTTTCAT | 0.00 | 0.00 | 0.00 | 0.00 | 269.49 | 0.00 | 0.00 | 0.00 | 0.00 | 0.00 | 0.00 | 0.00 | 0.00 | 0.00 | 269.49 |
| 17 | b | AAAGTAGAACTTTTGTC | 0.00 | 0.00 | 0.00 | 0.00 | 106.10 | 0.00 | 0.00 | 0.00 | 0.00 | 0.00 | 0.00 | 0.00 | 0.00 | 0.00 | 106.10 |
| 18 | a | AAAGAAGAAGAGAAGAGG | 0.00 | 0.00 | 0.00 | 0.00 | 0.00 | 0.00 | 0.00 | 57.56 | 0.00 | 0.00 | 0.00 | 0.00 | 0.00 | 0.00 | 57.56 |
| 18 | a | AAATTTCAAGGAACGTCG | 0.00 | 0.00 | 0.00 | 0.00 | 0.00 | 0.00 | 0.00 | 0.00 | 0.00 | 0.00 | 438.88 | 0.00 | 0.00 | 0.00 | 438.88 |
| 18 | b | ACGGTGCTGGACTCGGTT | 0.00 | 63.36 | 0.00 | 0.00 | 0.00 | 0.00 | 0.00 | 0.00 | 0.00 | 0.00 | 0.00 | 0.00 | 0.00 | 0.00 | 63.36 |
| 18 | a | AACGTGAACGTGATCGTG | 0.00 | 0.00 | 0.00 | 0.00 | 0.00 | 0.00 | 0.00 | 0.00 | 0.00 | 0.00 | 79.65 | 0.00 | 0.00 | 0.00 | 79.65 |
| 19 | a | AAGAAGTAAGAATAAGAGT | 0.00 | 0.00 | 0.00 | 0.00 | 0.00 | 0.00 | 0.00 | 0.00 | 0.00 | 0.00 | 168.68 | 0.00 | 0.00 | 0.00 | 168.68 |
| 20 | b | ACGTCCATCGTGGTCCATCT | 0.00 | 0.00 | 0.00 | 0.00 | 0.00 | 0.00 | 0.00 | 0.00 | 0.00 | 0.00 | 66.38 | 0.00 | 0.00 | 0.00 | 66.38 |
| 21 | a | AAAATGATGGAAATCAGGACG | 0.00 | 0.00 | 0.00 | 0.00 | 0.00 | 0.00 | 0.00 | 0.00 | 0.00 | 0.00 | 117.14 | 0.00 | 0.00 | 0.00 | 117.14 |
| 21 | a | AATAATAATAATGATAATGAT | 0.00 | 0.00 | 0.00 | 0.00 | 0.00 | 0.00 | 0.00 | 0.00 | 0.00 | 0.00 | 66.38 | 0.00 | 0.00 | 0.00 | 66.38 |
| 21 | a | AATAGTAGTAGTAGTAGTAGT | 0.00 | 0.00 | 0.00 | 0.00 | 0.00 | 0.00 | 0.00 | 0.00 | 0.00 | 0.00 | 136.66 | 0.00 | 0.00 | 0.00 | 136.66 |
| 21 | b | ACTGGTCCCGTACTGGTCCGT | 0.00 | 0.00 | 0.00 | 0.00 | 0.00 | 0.00 | 0.00 | 0.00 | 0.00 | 84.82 | 0.00 | 0.00 | 0.00 | 0.00 | 84.82 |
| 22 | b | AACTACTACATTTATTACTTTT | 63.81 | 0.00 | 0.00 | 0.00 | 0.00 | 0.00 | 0.00 | 0.00 | 0.00 | 0.00 | 0.00 | 0.00 | 0.00 | 0.00 | 63.81 |
| 22 | a | AAAGAATCGTCGAAGAATCGTC | 0.00 | 0.00 | 0.00 | 0.00 | 0.00 | 0.00 | 0.00 | 0.00 | 0.00 | 0.00 | 71.46 | 0.00 | 0.00 | 0.00 | 71.46 |
| 22 | a | ACCAGTACGGGACGAGTACGGG | 0.00 | 0.00 | 0.00 | 0.00 | 0.00 | 0.00 | 0.00 | 0.00 | 0.00 | 174.04 | 0.00 | 0.00 | 0.00 | 0.00 | 174.04 |
| 23 | a | ACCAGTACGGGACCCAGTACGGG | 0.00 | 0.00 | 0.00 | 0.00 | 0.00 | 0.00 | 0.00 | 0.00 | 0.00 | 93.21 | 0.00 | 0.00 | 0.00 | 0.00 | 93.21 |
| 23 | A | ACCAGTACGGGACCGAGTACGGG | 0.00 | 0.00 | 0.00 | 0.00 | 0.00 | 0.00 | 0.00 | 0.00 | 0.00 | 175.42 | 0.00 | 0.00 | 0.00 | 0.00 | 175.42 |
| 23 | B | ACTCGGTCCCGTACTGGTCCCGT | 0.00 | 0.00 | 0.00 | 0.00 | 0.00 | 0.00 | 0.00 | 0.00 | 41.90 | 158.93 | 0.00 | 0.00 | 0.00 | 0.00 | 158.93 |
| 24 | b | AAATTTTAGTTATATTTGAAGTTC | 0.00 | 0.00 | 0.00 | 0.00 | 0.00 | 0.00 | 0.00 | 0.00 | 0.00 | 0.00 | 151.89 | 6.69 | 0.53 | 0.00 | 151.89 |
| 24 | b | CGGTGTTGTCTTGTCTGTTGGCTT | 0.00 | 451.68 | 0.00 | 0.00 | 0.00 | 0.00 | 0.00 | 0.00 | 0.00 | 0.00 | 0.00 | 0.00 | 0.00 | 0.00 | 451.68 |
| 24 | a | AAGGCAGCGGAGTACTACACCACC | 0.00 | 0.00 | 79.43 | 6.75 | 22.22 | 0.00 | 0.00 | 0.00 | 0.00 | 0.00 | 0.00 | 0.00 | 0.00 | 0.00 | 79.43 |
| 24 | a | ACCGTCTGCTCCGCCGGCCCAGCC | 0.00 | 0.00 | 65.55 | 2.75 | 8.36 | 0.00 | 0.00 | 0.00 | 0.00 | 0.00 | 0.00 | 0.00 | 0.00 | 0.00 | 65.55 |
| 26 | b | AAATTTCCGTGTGAAGAGGAATTGAT | 0.00 | 0.00 | 0.00 | 0.00 | 0.00 | 0.00 | 0.00 | 0.00 | 0.00 | 0.00 | 0.00 | 53.63 | 6.32 | 0.00 | 53.63 |
| 28 | a | AAATAACTTATCAATTCCTCCTCGGACG | 0.00 | 0.00 | 0.00 | 0.00 | 0.00 | 0.00 | 0.00 | 0.00 | 0.00 | 0.00 | 0.00 | 56.94 | 0.00 | 0.00 | 56.94 |
| 30 | b | AAATACGTGCGTTTAGAAATTCCAGAAATC | 0.00 | 0.00 | 0.00 | 0.00 | 0.00 | 0.00 | 0.00 | 0.00 | 0.00 | 0.00 | 179.22 | 0.00 | 0.00 | 0.00 | 179.22 |
| 30 | b | ATGATGATGATGATGATGATGATGATGATT | 0.00 | 0.00 | 0.00 | 0.00 | 0.00 | 0.00 | 0.00 | 0.00 | 0.00 | 0.00 | 73.41 | 0.00 | 0.00 | 0.00 | 73.41 |
| 30 | b | AGTCCATCGTAGTCCATCTATGTCCATCGT | 0.00 | 0.00 | 0.00 | 0.00 | 0.00 | 0.00 | 0.00 | 0.00 | 0.00 | 0.00 | 99.18 | 0.00 | 0.00 | 0.00 | 99.18 |
| 30 | a | ACATAGATGGACTACGATGGGCTACGATGG | 0.00 | 0.00 | 0.00 | 0.00 | 0.00 | 0.00 | 0.00 | 0.00 | 0.00 | 0.00 | 105.04 | 0.00 | 0.00 | 0.00 | 105.04 |
| 31 | a | AAAAATAAATTATTTCGTTTGCCCACCCTTT | 0.00 | 0.00 | 0.00 | 0.00 | 0.00 | 0.00 | 0.00 | 0.00 | 157.22 | 36.33 | 0.00 | 0.00 | 0.00 | 0.00 | 157.22 |
| 31 | b | AAACGAAATAATTTAATTTTAAAGGGTGGGC | 0.00 | 0.00 | 0.00 | 0.00 | 0.00 | 0.00 | 0.00 | 0.00 | 53.34 | 48.49 | 0.00 | 0.00 | 0.00 | 0.00 | 53.34 |
| 31 | a | AAGTAGTAGTAGTAGTAGTAGTAGTAGTAGT | 0.00 | 0.00 | 0.00 | 0.00 | 0.00 | 0.00 | 0.00 | 0.00 | 0.00 | 0.00 | 106.21 | 0.00 | 0.00 | 0.00 | 106.21 |
| 33 | b | ACTCGGTCCCGTACTGGTCCCGTACTGGTCCGT | 0.00 | 0.00 | 0.00 | 0.00 | 0.00 | 0.00 | 0.00 | 0.00 | 0.00 | 476.83 | 0.00 | 0.00 | 0.00 | 0.00 | 476.83 |
| 34 | b | ATCGTTCCTGTGGCTTTATGCCGGCTTCGTGCCC | 0.00 | 0.00 | 53.12 | 0.00 | 2.06 | 0.00 | 0.00 | 0.00 | 0.00 | 0.00 | 0.00 | 0.00 | 0.00 | 0.00 | 53.12 |
| 34 | b | ACTCGGTCCCGTACTGGTCCCGTACTGGTCCCGT | 0.00 | 0.00 | 0.00 | 0.00 | 0.00 | 0.00 | 0.00 | 0.00 | 0.00 | 71.24 | 0.00 | 0.00 | 0.00 | 0.00 | 71.24 |
| 35 | b | ACTCGGTCCCGTACTCGGTCCCGTACTGGTCCCGT | 0.00 | 0.00 | 0.00 | 0.00 | 0.00 | 0.00 | 0.00 | 0.00 | 38.95 | 141.75 | 0.00 | 0.00 | 0.00 | 0.00 | 141.75 |
| 36 | b | AAATTGAAGTTACAATTTTTGTTATATTTGAAGTTC | 0.00 | 0.00 | 0.00 | 0.00 | 0.00 | 0.00 | 0.00 | 0.00 | 0.00 | 0.00 | 98.01 | 0.00 | 0.79 | 0.00 | 98.01 |
| 36 | b | AAAATTTTTGTTATATTTGAAGTTCAAGTTGAAGTT | 0.00 | 0.00 | 0.00 | 0.00 | 0.00 | 0.00 | 0.00 | 0.00 | 0.00 | 0.00 | 69.11 | 0.00 | 0.00 | 0.00 | 69.11 |
| 36 | b | AAAATTTTTGTTATATCTGAAGTTCAAATTGAAGTT | 0.00 | 0.00 | 0.00 | 0.00 | 0.00 | 0.00 | 0.00 | 0.00 | 0.00 | 0.00 | 251.07 | 59.83 | 5.15 | 0.00 | 251.07 |
| 36 | b | AAAATTTTTGTTATATCTGAAGTTCAAATGGAAGTT | 0.00 | 0.00 | 0.00 | 0.00 | 0.00 | 0.00 | 0.00 | 0.00 | 0.00 | 0.00 | 116.75 | 0.00 | 1.47 | 0.00 | 116.75 |
| 36 | b | ACCTTCTCCGTCCTTTCTCTCCTCAGCTTCATCTTC | 132.67 | 0.00 | 0.00 | 0.00 | 0.00 | 0.00 | 0.00 | 0.00 | 0.00 | 0.00 | 0.00 | 0.00 | 0.00 | 0.00 | 132.67 |
| 39 | a | AAACCAACTGAGGGAACGAGTGCCAAGCCAACAACTTTG | 0.00 | 0.00 | 0.00 | 0.00 | 0.00 | 0.00 | 0.00 | 407.43 | 0.00 | 0.00 | 0.00 | 0.00 | 0.00 | 0.00 | 407.43 |
| 45 | b | AAATATTCCAAGTATCAAGATCGAAGTAAGTTTGAGTAGATTGAG | 0.00 | 0.00 | 0.00 | 0.00 | 0.00 | 0.00 | 0.00 | 0.00 | 0.00 | 0.00 | 52.32 | 0.00 | 0.00 | 0.00 | 52.32 |
| 45 | a | AACGACGACAACGTCAACGACGACAACGTCAACGCCGACACCGAC | 0.00 | 0.00 | 0.00 | 0.00 | 0.00 | 0.00 | 0.00 | 0.00 | 0.00 | 0.00 | 102.69 | 0.00 | 0.00 | 0.00 | 102.69 |
| 45 | a | ACCAGTACGGGACCAGTACGGGACCAGTACGGGACCGAGTACGGG | 0.00 | 0.00 | 0.00 | 0.00 | 0.00 | 0.00 | 0.00 | 0.00 | 0.00 | 68.90 | 0.00 | 0.00 | 0.00 | 0.00 | 68.90 |
| 46 | a | ACCAGTACGGGACCAGTACGGGACCGAGTACGGGACCGAGTACGGG | 0.00 | 0.00 | 0.00 | 0.00 | 0.00 | 0.00 | 0.00 | 0.00 | 0.00 | 274.52 | 0.00 | 0.00 | 0.00 | 0.00 | 274.52 |
| 47 | b | AACACTCTTCCTTCTCGGATGGATTTGAGCAGTTGGAACGTGAAGAG | 0.00 | 0.00 | 139.52 | 0.00 | 0.00 | 0.00 | 0.00 | 0.00 | 0.00 | 0.00 | 0.00 | 0.00 | 0.00 | 0.00 | 139.52 |
| 47 | b | AACACTCTTCTCTTCCGGATGGATTTGAGCAGTTGGAACGTGAAGAG | 0.00 | 0.00 | 57.56 | 0.00 | 0.00 | 0.00 | 0.00 | 0.00 | 0.00 | 0.00 | 0.00 | 0.00 | 0.00 | 0.00 | 57.56 |
| 48 | a | AAAAATGTCAAAGATTGTTGTATAAGGGATATAATTGATTATTGTAAG | 0.00 | 0.00 | 0.00 | 0.00 | 0.00 | 0.00 | 0.00 | 0.00 | 0.00 | 0.00 | 63.65 | 0.00 | 0.00 | 0.00 | 63.65 |
| 48 | a | AACAACAACTTCCACAAGTGGCCCAACGACAACTACTCCTAGATCCAC | 0.00 | 0.00 | 0.00 | 0.00 | 0.00 | 0.00 | 0.00 | 123.60 | 0.00 | 0.00 | 0.00 | 0.00 | 0.00 | 0.00 | 123.60 |
| 48 | a | ACCGAGTACGGGACCGAGTACGGGACCGAGTACGGGACCTAGTACGGG | 0.00 | 0.00 | 0.00 | 0.00 | 0.00 | 0.00 | 0.00 | 0.00 | 0.00 | 122.28 | 0.00 | 0.00 | 0.00 | 0.00 | 122.28 |

**Additional file 7:** (b) Mean lengths of repeat types in different genomic regions of *Daphnia pulex* (Dappu), *Drosophila melanogaster* (DroMel) and *Apis mellifera* (ApiMel). A=normal B=reverse complement of repeat motif A; "-" denotes palindromic motifs; a and b are used if only the normal form (a) OR its reverse complement (b) were found.

| unit size [bp] | motif form | unit | Dappu v1.1 3UTR [bp] | Dappu v1.1 5UTR [bp] | Dappu v1.1 CDS [bp] | Dappu v1.1 introns [bp] | Dappu v1.1 intergenic [bp] | DroMel-5.5 3UTR [bp] | DroMel-5.5 5UTR [bp] | DroMel-5.5 CDS [bp] | DroMel-5.5 introns [bp] | DroMel-5.5 intergenic [bp] | ApiMel-2.0 CDS [bp] | ApiMel-2.0 introns [bp] | ApiMel-2.0 intergenic [bp] | min [bp] | max [bp] |
|---|---|---|---|---|---|---|---|---|---|---|---|---|---|---|---|---|---|
| 1 | A | A | 16.5 | 15.8 | 19.6 | 16.3 | 16.8 | 15.6 | 15.9 | 16.0 | 16.8 | 16.9 | 16.0 | 18.1 | 17.8 | 15.6 | 19.6 |
| 1 | B | T | 15.9 | 14.9 | 17.1 | 16.6 | 16.8 | 15.6 | 15.2 | 17.8 | 16.7 | 16.9 | 16.0 | 18.0 | 17.8 | 14.9 | 18.0 |
| 1 | A | C | 14.4 | 14.0 | 13.8 | 15.2 | 15.1 | 14.2 | 15.3 | | 14.8 | 14.8 | 13.0 | 14.7 | 14.7 | 13.0 | 15.3 |
| 1 | B | G | 15.2 | 17.0 | 14.8 | 15.1 | 15.1 | 16.3 | | | 14.7 | 15.0 | 13.0 | 15.0 | 15.3 | 13.0 | 17.0 |
| 2 | A | AC | 19.0 | 18.5 | 17.2 | 18.0 | 18.5 | 21.0 | 19.1 | 16.0 | 20.6 | 20.6 | 14.0 | 26.2 | 24.7 | 14.0 | 26.2 |
| 2 | B | GT | 17.3 | 22.4 | 17.0 | 18.2 | 18.2 | 21.7 | 17.4 | 23.0 | 20.4 | 20.6 | | 23.8 | 24.4 | 17.0 | 24.4 |
| 2 | A | AG | 17.0 | 17.3 | 16.2 | 19.0 | 18.8 | 22.5 | 22.2 | 22.0 | 21.5 | 21.4 | 17.3 | 23.9 | 23.7 | 16.2 | 23.9 |
| 2 | B | CT | 18.4 | 17.7 | 18.8 | 19.1 | 18.9 | 19.2 | 18.6 | 14.0 | 21.6 | 21.3 | 27.9 | 23.5 | 23.3 | 14.0 | 27.9 |
| 2 | - | AT | 17.5 | 14.0 | 15.0 | 18.0 | 18.1 | 24.1 | 23.1 | 24.0 | 24.2 | 24.5 | 22.0 | 27.2 | 26.8 | 14.0 | 27.2 |
| 3 | A | AAC | 17.9 | 19.5 | 20.7 | 20.0 | 19.6 | 23.3 | 20.0 | 22.2 | 22.1 | 21.8 | 31.4 | 21.3 | 23.1 | 17.9 | 31.4 |
| 3 | B | GTT | 18.7 | 21.7 | 19.5 | 19.7 | 20.0 | 18.0 | 18.1 | 24.0 | 21.3 | 21.5 | 25.5 | 22.0 | 23.9 | 18.0 | 25.5 |
| 3 | A | AAG | 18.1 | 18.3 | 22.6 | 19.8 | 20.6 | 22.0 | 16.7 | 19.2 | 18.7 | 18.3 | 38.3 | 24.1 | 24.4 | 16.7 | 38.3 |
| 3 | B | CTT | 19.0 | | 20.2 | 20.5 | 20.6 | 15.0 | 28.5 | 21.0 | 18.9 | 18.7 | 29.0 | 23.9 | 24.7 | 15.0 | 29.0 |
| 3 | A | AAT | 16.9 | 16.0 | 19.6 | 19.7 | 19.2 | 21.9 | 19.1 | 20.6 | 29.3 | 38.9 | 33.4 | 40.4 | 27.4 | 16.0 | 40.4 |
| 3 | B | ATT | 19.0 | 15.0 | 18.5 | 19.5 | 19.7 | 25.0 | 22.1 | 21.0 | 29.5 | 32.2 | 25.4 | 26.0 | 27.0 | 15.0 | 32.2 |
| 3 | A | ACC | 18.3 | 17.0 | 19.0 | 18.4 | 18.7 | 21.7 | 19.4 | 21.0 | 20.5 | 20.5 | 36.0 | 28.1 | 24.7 | 17.0 | 36.0 |
| 3 | B | GGT | | 19.0 | 18.5 | 18.6 | 18.5 | 22.0 | 22.7 | 20.9 | 20.3 | 20.7 | 34.1 | 21.7 | 25.2 | 18.5 | 34.1 |
| 3 | A | ACG | 18.0 | | 19.1 | 18.3 | 19.0 | 17.3 | 18.0 | 18.7 | 19.2 | 21.4 | 29.0 | 23.6 | 29.3 | 17.3 | 29.3 |
| 3 | B | CGT | | 15.0 | 19.4 | 18.8 | 18.7 | 16.3 | 20.3 | 21.4 | 18.6 | 19.2 | 45.8 | 29.2 | 27.5 | 15.0 | 45.8 |
| 3 | A | ACT | 27.7 | 30.0 | 22.6 | 21.7 | 21.1 | 20.3 | 15.0 | 21.0 | 25.2 | 22.8 | 136.8 | 48.4 | 44.8 | 15.0 | 136.8 |
| 3 | B | AGT | | | 19.3 | 20.8 | 20.8 | 26.0 | | 22.7 | 23.9 | 24.3 | 93.9 | 53.0 | 42.2 | 19.3 | 93.9 |
| 3 | A | AGC | 17.0 | 19.7 | 19.6 | 18.6 | 19.0 | 24.4 | 23.9 | 25.6 | 26.3 | 28.0 | 29.5 | 24.0 | 27.1 | 17.0 | 29.5 |
| 3 | B | CTG | 18.7 | 15.6 | 19.8 | 19.2 | 19.1 | 18.6 | 18.4 | 20.4 | 22.9 | 23.4 | 26.2 | 20.8 | 25.3 | 15.6 | 26.2 |
| 3 | A | AGG | | | 18.3 | 17.8 | 18.2 | 21.4 | 20.5 | 21.7 | 22.5 | 21.8 | 22.5 | 22.9 | 22.4 | 17.8 | 22.9 |
| 3 | B | CCT | 17.5 | 23.0 | 19.8 | 17.4 | 18.8 | 23.8 | 20.0 | 18.9 | 22.8 | 22.4 | 27.3 | 23.1 | 22.8 | 17.4 | 27.3 |
| 3 | A | ATC | 16.0 | 18.0 | 18.5 | 18.6 | 18.8 | 20.7 | 18.5 | 21.3 | 21.6 | 21.0 | 29.1 | 28.7 | 28.4 | 16.0 | 29.1 |
| 3 | B | ATG | 18.5 | | 18.6 | 18.3 | 18.7 | 22.8 | 19.0 | 20.3 | 21.3 | 21.1 | 51.6 | 34.9 | 27.9 | 18.3 | 51.6 |
| 3 | A | CCG | 19.0 | | 19.8 | 18.4 | 18.8 | 15.0 | 21.3 | 17.9 | 16.7 | 16.7 | 22.7 | 20.7 | 20.8 | 15.0 | 22.7 |
| 3 | B | CGG | | | 20.4 | 19.1 | 18.8 | 20.0 | 16.3 | 21.5 | 18.0 | 17.1 | 26.5 | 19.2 | 21.6 | 16.3 | 26.5 |
| 4 | A | AAAC | 21.3 | 16.5 | 20.5 | 19.7 | 21.2 | 19.9 | 22.2 | | 20.6 | 21.1 | | 28.7 | 21.8 | 16.5 | 28.7 |
| 4 | B | GTTT | 16.0 | | 20.7 | 20.5 | 21.3 | 23.5 | 22.8 | | 22.1 | 20.3 | | 18.0 | 20.5 | 16.0 | 23.5 |
| 4 | A | AAAG | 22.0 | | 16.0 | 24.0 | 23.7 | 19.4 | 21.0 | | 19.7 | 19.8 | 20.5 | 24.6 | 24.5 | 16.0 | 24.6 |
| 4 | B | CTTT | 19.0 | 22.0 | 17.6 | 21.3 | 22.2 | | | | 19.1 | 22.6 | 22.0 | 24.1 | 24.8 | 17.6 | 24.8 |
| 4 | A | AAAT | 21.6 | | | 20.2 | 20.0 | 19.2 | 21.1 | | 19.2 | 20.0 | | 21.8 | 22.7 | 19.2 | 22.7 |
| 4 | B | ATTT | 18.3 | | 16.0 | 20.7 | 20.2 | 20.1 | 20.5 | | 19.1 | 23.3 | 18.0 | 21.5 | 22.8 | 16.0 | 23.3 |
| 4 | a | AACC | 16.0 | | | 20.5 | 19.6 | 25.9 | 21.4 | 20.0 | 25.8 | 23.6 | | 19.0 | 24.0 | 16.0 | 25.9 |
| 4 | A | AAGG | | | 37.7 | 21.7 | 27.6 | 16.0 | | | 20.6 | 19.6 | 16.7 | 27.0 | 26.6 | 16.0 | 37.7 |
| 4 | B | CCTT | | | 18.0 | 23.3 | 20.7 | | | | 21.4 | 22.5 | | 23.4 | 26.2 | 18.0 | 26.2 |
| 4 | - | AATT | | | | 19.9 | 17.9 | 18.5 | 21.0 | | 19.5 | 22.3 | | 20.6 | 20.3 | 17.9 | 22.3 |
| 4 | A | ACAT | | | 16.0 | 27.4 | 24.0 | 18.6 | 20.3 | | 20.3 | 21.0 | | 28.7 | 29.0 | 16.0 | 29.0 |
| 4 | B | ATGT | | | | 22.0 | 23.1 | 18.0 | 17.5 | | 20.4 | 20.8 | | 29.3 | 27.2 | 17.5 | 29.3 |
| 4 | a | AGCC | | 16.0 | 26.8 | 20.2 | 20.7 | 19.4 | 26.7 | 19.0 | 24.3 | 24.4 | | 33.0 | 26.8 | 16.0 | 33.0 |
| 4 | A | AGGG | | | | 27.4 | 18.9 | | | | 17.3 | 25.0 | | 21.2 | 20.5 | 17.3 | 27.4 |
| 4 | B | CCCT | | | | 19.3 | 24.0 | | | | 20.7 | 26.0 | 16.0 | 20.2 | 20.0 | 16.0 | 26.0 |
| 4 | a | ATCC | 16.0 | | 38.0 | 33.5 | 25.1 | 19.5 | | | 25.3 | 25.5 | | 24.5 | 24.3 | 16.0 | 38.0 |
| 5 | A | AAAAC | 21.4 | | 19.0 | 20.0 | 22.7 | 22.0 | 25.7 | | 22.5 | 22.2 | | 21.3 | 21.7 | 19.0 | 25.7 |
| 5 | B | GTTTT | 18.3 | 17.0 | 17.0 | 20.2 | 21.6 | | 22.0 | | 21.0 | 21.1 | | 21.8 | 20.6 | 17.0 | 22.0 |
| 5 | A | AAAAG | 18.0 | 17.0 | 22.0 | 20.3 | 21.7 | 19.8 | 18.0 | | 26.0 | 41.3 | 21.0 | 25.0 | 25.0 | 17.0 | 41.3 |
| 5 | B | CTTTT | 21.5 | 25.0 | | 20.7 | 20.6 | 24.0 | | | 22.6 | 20.4 | 51.0 | 24.5 | 24.5 | 20.4 | 51.0 |
| 5 | A | AAAAT | 17.3 | 21.5 | | 20.6 | 22.6 | 19.3 | 26.3 | | 23.8 | 22.3 | | 23.2 | 22.9 | 17.3 | 26.3 |
| 5 | B | ATTTT | 20.5 | 17.0 | | 23.5 | 21.1 | 22.3 | | | 22.7 | 23.5 | | 23.6 | 24.9 | 17.0 | 24.9 |
| 5 | A | AACCT | | | | 37.0 | 74.8 | | | | | | | 37.0 | 25.3 | 25.3 | 74.8 |
| 5 | B | AGGTT | | | | 35.9 | 75.5 | | | | | | | 44.2 | 26.3 | 26.3 | 75.5 |
| 5 | A | AAGAT | | | | 24.3 | 27.1 | | | | 18.0 | 1137.4 | | 27.0 | 21.7 | 18.0 | 1137.4 |
| 5 | B | ATCTT | | | | 24.0 | 25.3 | | | | 19.2 | 236.3 | | 19.0 | 23.3 | 19.0 | 236.3 |
| 5 | A | AATAG | | | | 56.0 | 23.2 | | 18.0 | | 23.3 | 455.7 | 23.0 | 20.5 | 21.7 | 18.0 | 455.7 |
| 5 | B | ATTCT | | | | 22.2 | 30.6 | | 18.0 | | 25.6 | 701.1 | | 21.2 | 22.7 | 18.0 | 701.1 |
| 5 | A | AATAT | | | | 18.5 | 25.6 | 26.0 | | | 31.8 | 122.7 | | 31.2 | 35.8 | 18.5 | 122.7 |
| 5 | B | ATATT | 18.0 | | | 23.1 | 23.7 | 37.5 | 36.0 | | 54.9 | 83.4 | 17.0 | 31.3 | 33.8 | 17.0 | 83.4 |
| 5 | A | AATCG | | | | 18.0 | | 33.7 | 27.5 | | 27.7 | 23.9 | | 21.3 | 21.7 | 18.0 | 33.7 |
| 5 | B | ATTCG | | | | | 17.0 | | 26.6 | 17.0 | 24.9 | 25.5 | | 22.1 | 22.6 | 17.0 | 26.6 |
| 5 | A | ATCCG | | | 17.0 | | 20.0 | 22.0 | 24.3 | 18.0 | 22.5 | 23.0 | | 18.5 | 21.5 | 17.0 | 24.3 |
| 5 | B | ATCGG | | | | | 18.0 | 21.0 | 25.0 | 17.0 | 24.4 | 24.0 | | | 23.5 | 17.0 | 25.0 |
| 6 | a | AAAAAC | 24.7 | 18.0 | 23.0 | 21.3 | 26.9 | 28.0 | | | 22.9 | 23.3 | | | 22.8 | 18.0 | 28.0 |
| 6 | A | AAAAAG | 21.5 | | 25.8 | 22.4 | 22.4 | 22.0 | | 18.0 | 26.1 | 24.5 | 26.5 | 27.1 | 26.1 | 18.0 | 27.1 |
| 6 | B | CTTTTT | 20.5 | | | 23.8 | 23.3 | 21.0 | | | 22.2 | 25.0 | | 26.4 | 25.4 | 20.5 | 26.4 |
| 6 | b | ATTTTT | 18.0 | | | 23.1 | 21.7 | 23.0 | | | 22.6 | 22.9 | | 23.9 | 23.3 | 18.0 | 23.9 |
| 6 | a | AAAAGC | | | 29.0 | | 26.1 | 45.5 | 23.3 | | 25.7 | 23.6 | | | 19.5 | 19.5 | 45.5 |
| 6 | a | AAATAC | | | | | 22.7 | 27.0 | 18.0 | | 23.0 | 27.8 | 212.0 | | 23.7 | 18.0 | 212.0 |
| 6 | A | AAATAT | | | | 22.5 | 56.5 | 27.3 | 18.0 | | 21.4 | 24.5 | | 27.1 | 28.1 | 18.0 | 56.5 |
| 6 | B | ATATTT | | | | 29.0 | 20.5 | 18.0 | 22.0 | | 58.8 | 24.2 | | 66.9 | 26.8 | 18.0 | 66.9 |
| 6 | a | AACAAT | | | | | 20.7 | | | 22.1 | 20.8 | 24.5 | 108.8 | 41.0 | 30.2 | 20.7 | 108.8 |
| 6 | a | AACAGC | | | 25.9 | 44.3 | 23.1 | 28.0 | 27.4 | 30.4 | 26.4 | 26.3 | 24.0 | | 30.6 | 23.1 | 44.3 |
| 6 | A | AACCCC | | | | | 89.4 | | | | | 23.5 | | | | 23.5 | 89.4 |
| 6 | B | GGGGTT | | | | 18.0 | 93.3 | | | | 18.0 | 23.7 | | | | 18.0 | 93.3 |
| 6 | a | AAGAGT | | | | | | | | | | | 240.0 | | 18.0 | 18.0 | 240.0 |
| 6 | a | AAGCAG | | | 19.0 | 23.0 | 21.0 | 20.7 | 30.0 | 19.0 | 21.4 | 23.0 | 291.7 | 62.8 | 101.6 | 19.0 | 291.7 |
| 6 | a | AAGGCG | | | | 20.0 | | | | | | 34.0 | 272.0 | | 22.5 | 20.0 | 272.0 |
| 6 | A | AATAGT | | | | 19.0 | 24.3 | 18.0 | | | | 27.7 | 57.7 | 38.8 | 25.6 | 18.0 | 57.7 |
| 6 | B | ACTATT | | | | | | 29.0 | | | 23.0 | 25.5 | 99.0 | 24.0 | 38.0 | 23.0 | 99.0 |
| 6 | a | AATCGG | | | | | | | 29.7 | | 25.9 | 22.4 | | | 21.0 | 21.0 | 29.7 |
| 6 | a | AATGAC | | | | | 26.0 | | 21.0 | | 26.0 | 33.0 | 75.0 | | 47.0 | 21.0 | 75.0 |
| 6 | a | AATGAT | | | | 26.0 | | 18.0 | | 37.0 | 21.8 | 24.2 | 52.4 | 42.5 | 24.8 | 18.0 | 52.4 |
| 6 | A | ACAGAT | | | | | | 33.8 | 25.9 | 34.0 | 31.6 | 40.8 | | 20.0 | 19.0 | 19.0 | 40.8 |
| 6 | B | ATCTGT | | | | | 21.7 | 35.5 | | 31.0 | 30.3 | 27.8 | | | 24.0 | 21.7 | 35.5 |
| 6 | b | CGGTCT | | | | | 27.0 | | | | | 19.0 | 311.0 | | 21.0 | 19.0 | 311.0 |
| 6 | b | ATCGTG | | | | | 25.0 | | | 24.0 | 18.0 | | 187.0 | | 66.2 | 18.0 | 187.0 |
| 6 | a | ATCCGC | | | | | | 24.0 | 23.3 | 26.4 | 27.1 | 25.5 | | | | 23.3 | 27.1 |
| 7 | a | AAAAAAC | 19.0 | | 19.0 | 23.2 | 26.3 | 26.0 | 23.0 | | 25.3 | 23.4 | | 35.0 | 25.6 | 19.0 | 35.0 |
| 7 | A | AAAAAAG | | | | 24.9 | 22.8 | | 25.0 | | 29.4 | 21.5 | 20.5 | 26.5 | 25.8 | 20.5 | 29.4 |
| 7 | B | CTTTTTT | | | | 23.3 | 23.5 | | | | 26.8 | | 26.0 | 25.5 | 26.3 | 23.3 | 26.8 |
| 7 | A | AATATAT | | | | 37.5 | 41.0 | | | | 40.7 | 97.0 | | 39.8 | 35.9 | 35.9 | 97.0 |
| 7 | B | ATATATT | | | | 25.8 | 27.8 | 19.0 | | | 62.6 | 86.6 | | 33.6 | 38.0 | 19.0 | 86.6 |
| 8 | A | AAAAAAAG | | | | 25.1 | 24.3 | 20.0 | 20.0 | | 22.4 | 22.8 | 23.0 | 30.8 | 28.4 | 20.0 | 30.8 |
| 8 | B | CTTTTTTT | | | | 24.0 | 24.2 | | | | | 23.7 | | 26.6 | 27.7 | 23.7 | 27.7 |
| 8 | a | ACATATAT | | | | | 37.0 | 24.6 | 21.0 | | 26.3 | 296.0 | | 33.2 | 29.2 | 21.0 | 296.0 |
| 9 | b | CTTTTTTTT | | 40.0 | | 24.1 | 23.9 | | | | | 28.0 | | 34.4 | 28.6 | 23.9 | 40.0 |
| 9 | a | AACAGCAGC | | | 28.3 | 27.0 | 27.4 | 33.0 | 41.3 | 37.9 | 31.8 | 38.5 | 50.0 | | 41.9 | 27.0 | 50.0 |

| | | | | | | | | | | | | | | | | | |
|---|---|---|---|---|---|---|---|---|---|---|---|---|---|---|---|---|---|
| 9 | a | AATAATCAT | | | | | 26.0 | | | | 1024.7 | 27.0 | | 25.0 | 22.8 | 22.8 | 1024.7 |
| 9 | b | ACTACTATT | | | | | 23.0 | | | | | | 129.5 | | 77.0 | 23.0 | 129.5 |
| 9 | a | AATATATAT | | | | 33.0 | 48.5 | | | | 49.3 | 33.2 | | 46.0 | 42.3 | 33.0 | 49.3 |
| 10 | a | AAAGCTCGAG | | | | | | | | | | | 405.0 | | | 405.0 | 405.0 |
| 10 | b | AAGGTTCGCC | | | 542.2 | | 386.0 | | | | | | | | | 386.0 | 542.2 |
| 10 | b | ACTTGGATGG | | | | | | | | | | | 303.0 | 239.0 | 151.0 | 151.0 | 303.0 |
| 10 | b | ATCGTGGTCC | | | | | | | | | | | 388.0 | | | 388.0 | 388.0 |
| 10 | a | ACCATGGACT | | | | | | | | | | | 361.0 | | | 361.0 | 361.0 |
| 10 | b | ACGGTGGACT | | | | | | | | | | | 246.0 | | 71.0 | 71.0 | 246.0 |
| 10 | a | ACGATGGACT | | | | | | | | | | | 100.0 | 610.0 | 266.0 | 100.0 | 610.0 |
| 10 | b | CGTGCTCTGG | | | 1033.0 | | 160.7 | | | | | | | | | 160.7 | 1033.0 |
| 10 | A | ACGCCAGTGC | | | 384.0 | | 460.7 | | | | | | | | | 384.0 | 460.7 |
| 10 | B | ACTGGCGTGC | | | | | 501.0 | | | | | | | | | 501.0 | 501.0 |
| 11 | a | AAACAGCACTC | | 44.0 | | | | | | | | | | | | 44.0 | 44.0 |
| 11 | A | ACCAGTACGGG | | | | | | | | | 228.0 | 2862.7 | | | | 228.0 | 2862.7 |
| 11 | B | ACTGGTCCCGT | | | | | | | | 1308.0 | 304.4 | 2825.0 | | | | 304.4 | 2825.0 |
| 12 | a | AACAGCAGCAGC | | | 30.7 | 36.0 | 28.0 | 33.0 | | 44.6 | 48.0 | 39.2 | 32.0 | | 35.0 | 28.0 | 48.0 |
| 12 | b | ATGCCTGGTTGC | 100.0 | | | | | | | | | | | | | 100.0 | 100.0 |
| 12 | a | AATGATGATGAT | | | | | | 28.0 | | | 28.0 | | 69.5 | | 44.0 | 28.0 | 69.5 |
| 12 | A | ACCGAGTACGGG | | | | | | | | | 305.0 | 2058.1 | | | | 305.0 | 2058.1 |
| 12 | B | ACTCGGTCCCGT | | | | | | | | | 284.0 | 2914.3 | | | | 284.0 | 2914.3 |
| 12 | a | AGCAGCCTATCC | | 58.0 | | | | | | | | | | | | 58.0 | 58.0 |
| 15 | a | AAAAATAGTGGACCG | | | | | | | | | | | 499.0 | | | 499.0 | 499.0 |
| 15 | a | AAAAGTCTACTGATT | | 35.0 | | | | | | | | | | | | 35.0 | 35.0 |
| 15 | b | AACTTAGTTTATTTG | | | | | | | | | | | 364.8 | 62.5 | | 62.5 | 364.8 |
| 15 | b | AACTTAGTTCATTTG | | | | | | | | | | | 73.0 | | | 73.0 | 73.0 |
| 15 | b | ACATTTGCTCCGCCG | | | | | | | | | | | 141.0 | | | 141.0 | 141.0 |
| 15 | a | AACCCAGCCTGACCC | | | | | | | | | | | 410.0 | | | 410.0 | 410.0 |
| 15 | a | AAGTCACGGAGGACG | | | | | | | | | | | 351.0 | | | 351.0 | 351.0 |
| 15 | b | ACGATGGTGTCGATG | | | | | | | | | | | 215.0 | | | 215.0 | 215.0 |
| 16 | a | AAAAATCTCAAAGACC | | | | | | | | | | | 586.0 | | | 586.0 | 586.0 |
| 16 | a | AAATTCGAAGGCATCC | | | | | | | | | | | 728.0 | | | 728.0 | 728.0 |
| 17 | A | AAAAGTAGAACTTTTCT | | | | | 716.7 | | | | | | | | | 716.7 | 716.7 |
| 17 | B | AAAAGTTCTACTTTTAG | | | | | 785.4 | | | | | | | | | 785.4 | 785.4 |
| 17 | A | AAAAGTTCAACTTTATG | | | | | 315.8 | | | | | | | | | 315.8 | 315.8 |
| 17 | B | AAAGTTGAACTTTTCAT | | | | | 316.7 | | | | | | | | | 316.7 | 316.7 |
| 17 | b | AAAGTAGAACTTTTGTC | | | | | 681.5 | | | | | | | | | 681.5 | 681.5 |
| 18 | a | AAAGAAGAAGAGAAGAGG | | | | | | | | 1311.0 | | | | | | 1311.0 | 1311.0 |
| 18 | a | AAATTTCAAGGAACGTCG | | | | | | | | | | | 1124.0 | | | 1124.0 | 1124.0 |
| 18 | b | ACGGTGCTGGACTCGGTT | | 39.0 | | | | | | | | | | | | 39.0 | 39.0 |
| 18 | a | AACGTGAACGTGATCGTG | | | | | | | | | | | 204.0 | | | 204.0 | 204.0 |
| 19 | a | AAGAAGTAAGAATAAGAGT | | | | | | | | | | | 432.0 | | | 432.0 | 432.0 |
| 20 | b | ACGTCCATCGTGGTCCATCT | | | | | | | | | | | 170.0 | | | 170.0 | 170.0 |
| 21 | a | AAAATGATGGAAATCAGGACG | | | | | | | | | | | 100.0 | | | 100.0 | 100.0 |
| 21 | a | AATAATAATAATGATAATGAT | | | | | | | | | | | 170.0 | | | 170.0 | 170.0 |
| 21 | a | AATAGTAGTAGTAGTAGTAGT | | | | | | | | | | | 350.0 | | | 350.0 | 350.0 |
| 21 | b | ACTGGTCCCGTACTGGTCCGT | | | | | | | | | | 5404.0 | | | | 5404.0 | 5404.0 |
| 22 | b | AACTACTACATTTATTACTTTT | 101.0 | | | | | | | | | | | | | 101.0 | 101.0 |
| 22 | a | AAAGAATCGTCGAAGAATCGTC | | | | | | | | | | | 183.0 | | | 183.0 | 183.0 |
| 22 | a | ACCAGTACGGGACGAGTACGGG | | | | | | | | | | 11089.0 | | | | 11089.0 | 11089.0 |
| 23 | a | ACCAGTACGGGACCCAGTACGGG | | | | | | | | | | 5939.0 | | | | 5939.0 | 5939.0 |
| 23 | A | ACCAGTACGGGACCGAGTACGGG | | | | | | | | | | 3725.7 | | | | 3725.7 | 3725.7 |
| 23 | B | ACTCGGTCCCGTACTGGTCCCGT | | | | | | | | | 2158.0 | 2531.5 | | | | 2158.0 | 2531.5 |
| 24 | b | AAATTTTAGTTATATTTGAAGTTC | | | | | | | | | | | 129.7 | 109.0 | 109.0 | 109.0 | 129.7 |
| 24 | b | CGGTGTTGTCTTGTCTGTTGGCTT | | 278.0 | | | | | | | | | | | | 278.0 | 278.0 |
| 24 | a | AAGGCAGCGGAGTACTACACCACC | | | 203.8 | 66.3 | 267.5 | | | | | | | | | 66.3 | 267.5 |
| 24 | a | ACCGTCTGCTCCGCCGGCCCAGCC | | | 129.4 | 81.0 | 161.0 | | | | | | | | | 81.0 | 161.0 |
| 26 | b | AAATTTCCGTGTGAAGAGGAATTGAT | | | | | | | | | | | | 291.3 | 324.8 | 291.3 | 324.8 |
| 28 | a | AAATAACTTATCAATTCCTCCTCGGACG | | | | | | | | | | | | 928.0 | | 928.0 | 928.0 |
| 30 | b | AAATACGTGCGTTTAGAAATTCCAGAAATC | | | | | | | | | | | 459.0 | | | 459.0 | 459.0 |
| 30 | b | ATGATGATGATGATGATGATGATGATGATT | | | | | | | | | | | 188.0 | | | 188.0 | 188.0 |
| 30 | b | AGTCCATCGTAGTCCATCTATGTCCATCGT | | | | | | | | | | | 127.0 | | | 127.0 | 127.0 |
| 30 | a | ACATAGATGGACTACGATGGGCTACGATGG | | | | | | | | | | | 269.0 | | | 269.0 | 269.0 |
| 31 | a | AAAAATAAATTATTTCGTTTGCCCACCCTTT | | | | | | | | | 809.7 | 463.0 | | | | 463.0 | 809.7 |
| 31 | b | AAACGAAATAATTTAATTTTAAAGGGTGGGC | | | | | | | | | 686.8 | 1545.0 | | | | 686.8 | 1545.0 |
| 31 | a | AAGTAGTAGTAGTAGTAGTAGTAGTAGTAGT | | | | | | | | | | | 272.0 | | | 272.0 | 272.0 |
| 33 | b | ACTCGGTCCCGTACTGGTCCCGTACTGGTCCGT | | | | | | | | | | 30381.0 | | | | 30381.0 | 30381.0 |
| 34 | b | ATCGTTCCTGTGGCTTTATGCCGGCTTCGTGCCC | | | 454.3 | | 198.0 | | | | | | | | | 198.0 | 454.3 |
| 34 | b | ACTCGGTCCCGTACTGGTCCCGTACTGGTCCCGT | | | | | | | | | | 2269.5 | | | | 2269.5 | 2269.5 |
| 35 | b | ACTCGGTCCCGTACTCGGTCCCGTACTGGTCCCGT | | | | | | | | | 2006.0 | 9031.0 | | | | 2006.0 | 9031.0 |
| 36 | b | AAATTGAAGTTACAATTTTTGTTATATTTGAAGTTC | | | | | | | | | | | 125.5 | | 163.0 | 125.5 | 163.0 |
| 36 | b | AAAATTTTTGTTATATTTGAAGTTCAAGTTGAAGTT | | | | | | | | | | | 177.0 | | | 177.0 | 177.0 |
| 36 | b | AAAATTTTTGTTATATCTGAAGTTCAAATTGAAGTT | | | | | | | | | | | 321.5 | 162.5 | 211.4 | 162.5 | 321.5 |
| 36 | b | AAAATTTTTGTTATATCTGAAGTTCAAATGGAAGTT | | | | | | | | | | | 149.5 | | 302.0 | 149.5 | 302.0 |
| 36 | b | ACCTTCTCCGTCCTTTCTCTCCTCAGCTTCATCTTC | 210.0 | | | | | | | | | | | | | 210.0 | 210.0 |
| 39 | a | AAACCAACTGAGGGAACGAGTGCCAAGCCAACAACTTTG | | | | | | | | 9279.0 | | | | | | 9279.0 | 9279.0 |
| 45 | b | AAATATTCCAAGTATCAAGATCGAAGTAAGTTTGAGTAGATTGAG | | | | | | | | | | | 134.0 | | | 134.0 | 134.0 |
| 45 | a | AACGACGACAACGTCAACGACGACAACGTCAACGCCGACACCGAC | | | | | | | | | | | 263.0 | | | 263.0 | 263.0 |
| 45 | a | ACCAGTACGGGACCAGTACGGGACCAGTACGGGACCGAGTACGGG | | | | | | | | | | 2195.0 | | | | 2195.0 | 2195.0 |
| 46 | a | ACCAGTACGGGACCAGTACGGGACCGAGTACGGGACCGAGTACGGG | | | | | | | | | | 8745.5 | | | | 8745.5 | 8745.5 |
| 47 | b | AACACTCTTCCTTCTCGGATGGATTTGAGCAGTTGGAACGTGAAGAG | | | 1790.0 | | | | | | | | | | | 1790.0 | 1790.0 |
| 47 | b | AACACTCTTCTCTTCCGGATGGATTTGAGCAGTTGGAACGTGAAGAG | | | 1477.0 | | | | | | | | | | | 1477.0 | 1477.0 |
| 48 | a | AAAAATGTCAAAGATTGTTGTATAAGGGATATAATTGATTATTGTAAG | | | | | | | | | | | 163.0 | | | 163.0 | 163.0 |
| 48 | a | AACAACAACTTCCACAAGTGGCCCAACGACAACTACTCCTAGATCCAC | | | | | | | | 2815.0 | | | | | | 2815.0 | 2815.0 |
| 48 | a | ACCGAGTACGGGACCGAGTACGGGACCGAGTACGGGACCTAGTACGGG | | | | | | | | | | 7791.0 | | | | 7791.0 | 7791.0 |

**Additional file 7:** (c) Number of satellites found for a certain repeat motif in the different genomic regions of *Daphnia pulex* (Dappu), *Drosophila melanogaster* (DroMel) and *Apis mellifera* (ApiMel). A=normal B=reverse complement of repeat motif A; "-" denotes palindromic motifs; a and b are used if only the normal form (a) OR its reverse complement (b) were found.

| unit size | motif | | Dappu v1.1 3UTR | Dappu v1.1 5UTR | Dappu v1.1 CDS | Dappu v1.1 introns | Dappu v1.1 intergenic | DroMel-5.5 3UTR | DroMel-5.5 5UTR | DroMel-5.5 CDS | DroMel-5.5 introns | DroMel-5.5 intergenic | ApiMel-2.0 CDS | ApiMel-2.0 introns | ApiMel-2.0 intergenic | min | max |
|---|---|---|---|---|---|---|---|---|---|---|---|---|---|---|---|---|---|
| [bp] | form | unit | #sat | #sat | #sat | #sat | #sat | #sat | #sat | #sat | #sat | #sat | #sat | #sat | #sat | #sat | #sat |
| 1 | A | A | 83 | 13 | 15 | 1446 | 3647 | 259 | 145 | 7 | 3425 | 3023 | 17 | 1773 | 23843 | 7 | 23843 |
| 1 | B | T | 116 | 11 | 11 | 2289 | 3711 | 148 | 56 | 5 | 3067 | 3040 | 3 | 2534 | 23671 | 3 | 23671 |
| 1 | A | C | 43 | 1 | 9 | 971 | 1888 | 22 | 3 | 0 | 385 | 367 | 1 | 30 | 535 | 0 | 1888 |
| 1 | B | G | 15 | 1 | 6 | 908 | 1915 | 3 | 0 | 0 | 377 | 417 | 1 | 30 | 542 | 0 | 1915 |
| 2 | A | AC | 42 | 6 | 11 | 945 | 2329 | 209 | 98 | 1 | 1756 | 1890 | 1 | 100 | 1393 | 1 | 2329 |
| 2 | B | GT | 39 | 12 | 3 | 1246 | 2338 | 34 | 123 | 5 | 1935 | 1995 | 0 | 115 | 1334 | 0 | 2338 |
| 2 | A | AG | 17 | 3 | 10 | 1208 | 2588 | 28 | 56 | 2 | 372 | 393 | 3 | 922 | 13941 | 2 | 13941 |
| 2 | B | CT | 43 | 15 | 12 | 1458 | 2638 | 15 | 21 | 1 | 600 | 465 | 40 | 972 | 13582 | 1 | 13582 |
| 2 | - | AT | 20 | 2 | 2 | 342 | 680 | 451 | 84 | 7 | 1886 | 1872 | 1 | 2441 | 29688 | 1 | 29688 |
| 3 | A | AAC | 14 | 2 | 407 | 385 | 931 | 50 | 75 | 273 | 316 | 235 | 23 | 15 | 406 | 2 | 931 |
| 3 | B | GTT | 6 | 3 | 18 | 586 | 958 | 3 | 14 | 1 | 280 | 302 | 6 | 25 | 418 | 1 | 958 |
| 3 | A | AAG | 14 | 4 | 294 | 957 | 2215 | 1 | 9 | 36 | 40 | 41 | 54 | 193 | 3042 | 1 | 3042 |
| 3 | B | CTT | 10 | 0 | 37 | 1173 | 2324 | 1 | 2 | 2 | 59 | 41 | 5 | 236 | 3061 | 0 | 3061 |
| 3 | A | AAT | 12 | 1 | 8 | 209 | 549 | 28 | 9 | 37 | 247 | 351 | 28 | 409 | 4476 | 1 | 4476 |
| 3 | B | ATT | 18 | 1 | 4 | 330 | 599 | 39 | 7 | 1 | 289 | 380 | 9 | 404 | 4284 | 1 | 4284 |
| 3 | A | ACC | 3 | 2 | 127 | 149 | 347 | 17 | 11 | 133 | 89 | 58 | 25 | 16 | 370 | 2 | 370 |
| 3 | B | GGT | 0 | 1 | 58 | 153 | 315 | 1 | 3 | 55 | 62 | 77 | 35 | 27 | 362 | 0 | 362 |
| 3 | A | ACG | 1 | 0 | 126 | 150 | 387 | 3 | 6 | 56 | 38 | 34 | 14 | 41 | 605 | 0 | 605 |
| 3 | B | CGT | 0 | 1 | 49 | 167 | 358 | 3 | 3 | 9 | 69 | 46 | 4 | 32 | 650 | 0 | 650 |
| 3 | A | ACT | 3 | 1 | 8 | 101 | 193 | 6 | 1 | 2 | 55 | 36 | 14 | 14 | 173 | 1 | 193 |
| 3 | B | AGT | 0 | 0 | 3 | 91 | 176 | 1 | 0 | 3 | 38 | 32 | 20 | 33 | 182 | 0 | 182 |
| 3 | A | AGC | 2 | 6 | 445 | 514 | 1184 | 74 | 115 | 1474 | 514 | 494 | 19 | 5 | 205 | 2 | 1474 |
| 3 | B | CTG | 6 | 5 | 97 | 515 | 1262 | 9 | 7 | 30 | 393 | 429 | 17 | 6 | 156 | 5 | 1262 |
| 3 | A | AGG | 0 | 0 | 59 | 83 | 184 | 22 | 15 | 175 | 73 | 63 | 22 | 159 | 2838 | 0 | 2838 |
| 3 | B | CCT | 2 | 1 | 28 | 96 | 170 | 4 | 2 | 26 | 66 | 71 | 20 | 169 | 2842 | 1 | 2842 |
| 3 | A | ATC | 5 | 3 | 34 | 112 | 235 | 9 | 10 | 50 | 176 | 199 | 9 | 17 | 297 | 3 | 297 |
| 3 | B | ATG | 2 | 0 | 27 | 117 | 222 | 19 | 4 | 33 | 228 | 189 | 49 | 28 | 301 | 0 | 301 |
| 3 | A | CCG | 1 | 0 | 77 | 58 | 208 | 1 | 4 | 35 | 23 | 51 | 6 | 15 | 238 | 0 | 238 |
| 3 | B | CGG | 0 | 0 | 92 | 72 | 198 | 6 | 3 | 58 | 29 | 50 | 2 | 14 | 262 | 0 | 262 |
| 4 | A | AAAC | 3 | 2 | 2 | 49 | 147 | 18 | 12 | 0 | 87 | 94 | 0 | 3 | 57 | 0 | 147 |
| 4 | B | GTTT | 2 | 0 | 3 | 115 | 157 | 2 | 4 | 0 | 83 | 70 | 0 | 3 | 84 | 0 | 157 |
| 4 | A | AAAG | 1 | 0 | 1 | 134 | 284 | 7 | 2 | 0 | 17 | 15 | 6 | 149 | 2165 | 0 | 2165 |
| 4 | B | CTTT | 4 | 1 | 5 | 195 | 295 | 0 | 0 | 0 | 20 | 11 | 1 | 188 | 2212 | 0 | 2212 |
| 4 | A | AAAT | 7 | 0 | 0 | 54 | 152 | 11 | 11 | 0 | 123 | 215 | 0 | 129 | 1759 | 0 | 1759 |
| 4 | B | ATTT | 3 | 0 | 1 | 206 | 189 | 15 | 2 | 0 | 182 | 239 | 1 | 178 | 1763 | 0 | 1763 |
| 4 | a | AACC | 1 | 0 | 0 | 10 | 34 | 17 | 10 | 1 | 66 | 32 | 0 | 2 | 36 | 0 | 66 |
| 4 | A | AAGG | 0 | 0 | 3 | 14 | 42 | 1 | 0 | 0 | 9 | 7 | 3 | 50 | 655 | 0 | 655 |
| 4 | B | CCTT | 0 | 0 | 1 | 31 | 39 | 0 | 0 | 0 | 14 | 6 | 0 | 38 | 772 | 0 | 772 |
| 4 | - | AATT | 0 | 0 | 0 | 26 | 28 | 4 | 2 | 0 | 47 | 41 | 0 | 43 | 557 | 0 | 557 |
| 4 | A | ACAT | 0 | 0 | 1 | 14 | 180 | 26 | 12 | 0 | 216 | 205 | 0 | 23 | 224 | 0 | 224 |
| 4 | B | ATGT | 0 | 0 | 0 | 21 | 164 | 15 | 2 | 0 | 174 | 186 | 0 | 15 | 234 | 0 | 234 |
| 4 | a | AGCC | 0 | 1 | 4 | 33 | 97 | 7 | 6 | 1 | 68 | 54 | 0 | 8 | 72 | 0 | 97 |
| 4 | A | AGGG | 0 | 0 | 0 | 7 | 25 | 0 | 0 | 0 | 3 | 1 | 0 | 38 | 565 | 0 | 565 |
| 4 | B | CCCT | 0 | 0 | 0 | 8 | 13 | 0 | 0 | 0 | 3 | 2 | 1 | 33 | 569 | 0 | 569 |
| 4 | a | ATCC | 2 | 0 | 2 | 60 | 100 | 2 | 0 | 0 | 54 | 66 | 0 | 8 | 135 | 0 | 135 |
| 5 | A | AAAAC | 8 | 0 | 1 | 62 | 135 | 25 | 3 | 0 | 35 | 30 | 0 | 3 | 49 | 0 | 135 |
| 5 | B | GTTTT | 3 | 1 | 2 | 84 | 142 | 0 | 1 | 0 | 44 | 21 | 0 | 4 | 47 | 0 | 142 |
| 5 | A | AAAAG | 2 | 1 | 2 | 99 | 181 | 6 | 1 | 0 | 23 | 16 | 2 | 75 | 975 | 0 | 975 |
| 5 | B | CTTTT | 2 | 1 | 0 | 122 | 177 | 1 | 0 | 0 | 16 | 18 | 1 | 90 | 1008 | 0 | 1008 |
| 5 | A | AAAAT | 3 | 2 | 0 | 56 | 244 | 7 | 4 | 0 | 51 | 59 | 0 | 53 | 767 | 0 | 767 |
| 5 | B | ATTTT | 2 | 1 | 0 | 166 | 232 | 3 | 0 | 0 | 47 | 54 | 0 | 79 | 764 | 0 | 764 |
| 5 | A | AACCT | 0 | 0 | 0 | 8 | 77 | 0 | 0 | 0 | 0 | 0 | 0 | 7 | 146 | 0 | 146 |
| 5 | B | AGGTT | 0 | 0 | 0 | 10 | 67 | 0 | 0 | 0 | 0 | 0 | 0 | 13 | 187 | 0 | 187 |
| 5 | A | AAGAT | 0 | 0 | 0 | 6 | 17 | 0 | 0 | 0 | 1 | 10 | 0 | 1 | 34 | 0 | 34 |
| 5 | B | ATCTT | 0 | 0 | 0 | 5 | 23 | 0 | 0 | 0 | 5 | 16 | 0 | 2 | 24 | 0 | 24 |
| 5 | A | AATAG | 0 | 0 | 0 | 2 | 17 | 0 | 1 | 0 | 6 | 9 | 1 | 4 | 38 | 0 | 38 |
| 5 | B | ATTCT | 0 | 0 | 0 | 5 | 11 | 0 | 1 | 0 | 5 | 13 | 0 | 5 | 32 | 0 | 32 |
| 5 | A | AATAT | 0 | 0 | 0 | 4 | 14 | 1 | 0 | 0 | 29 | 65 | 0 | 90 | 1022 | 0 | 1022 |
| 5 | B | ATATT | 1 | 0 | 0 | 14 | 11 | 2 | 2 | 0 | 29 | 52 | 2 | 85 | 994 | 0 | 994 |
| 5 | A | AATCG | 0 | 0 | 0 | 2 | 0 | 3 | 6 | 0 | 42 | 41 | 0 | 9 | 81 | 0 | 81 |
| 5 | B | ATTCG | 0 | 0 | 0 | 0 | 1 | 0 | 8 | 1 | 34 | 37 | 0 | 7 | 86 | 0 | 86 |
| 5 | A | ATCCG | 0 | 0 | 1 | 0 | 3 | 2 | 7 | 1 | 24 | 38 | 0 | 2 | 13 | 0 | 38 |
| 5 | B | ATCGG | 0 | 0 | 0 | 0 | 1 | 2 | 7 | 1 | 31 | 26 | 0 | 0 | 13 | 0 | 31 |
| 6 | a | AAAAAC | 3 | 1 | 1 | 13 | 46 | 11 | 0 | 0 | 25 | 30 | 0 | 0 | 18 | 0 | 46 |
| 6 | A | AAAAAG | 2 | 0 | 4 | 57 | 163 | 1 | 0 | 2 | 8 | 8 | 2 | 49 | 703 | 0 | 703 |
| 6 | B | CTTTTT | 2 | 0 | 0 | 86 | 143 | 1 | 0 | 0 | 6 | 5 | 0 | 50 | 656 | 0 | 656 |
| 6 | b | ATTTTT | 1 | 0 | 0 | 77 | 94 | 1 | 0 | 0 | 39 | 33 | 0 | 34 | 379 | 0 | 379 |
| 6 | a | AAAAGC | 0 | 0 | 1 | 0 | 7 | 2 | 6 | 0 | 20 | 17 | 0 | 0 | 2 | 0 | 20 |
| 6 | a | AAATAC | 0 | 0 | 0 | 0 | 3 | 1 | 1 | 0 | 11 | 10 | 1 | 0 | 9 | 0 | 11 |
| 6 | A | AAATAT | 0 | 0 | 0 | 2 | 2 | 3 | 1 | 0 | 21 | 20 | 0 | 29 | 376 | 0 | 376 |
| 6 | B | ATATTT | 0 | 0 | 0 | 4 | 4 | 1 | 1 | 0 | 18 | 26 | 0 | 30 | 358 | 0 | 358 |
| 6 | a | AACAAT | 0 | 0 | 0 | 0 | 3 | 0 | 0 | 8 | 11 | 8 | 4 | 3 | 31 | 0 | 31 |
| 6 | a | AACAGC | 0 | 0 | 15 | 4 | 9 | 14 | 12 | 113 | 43 | 40 | 4 | 0 | 31 | 0 | 113 |
| 6 | A | AACCCC | 0 | 0 | 0 | 0 | 79 | 0 | 0 | 0 | 0 | 2 | 0 | 0 | 0 | 0 | 79 |
| 6 | B | GGGGTT | 0 | 0 | 0 | 1 | 75 | 0 | 0 | 0 | 1 | 3 | 0 | 0 | 0 | 0 | 75 |
| 6 | a | AAGAGT | 0 | 0 | 0 | 0 | 0 | 0 | 0 | 0 | 0 | 0 | 2 | 0 | 1 | 0 | 2 |
| 6 | a | AAGCAG | 0 | 0 | 1 | 1 | 3 | 3 | 1 | 2 | 5 | 4 | 13 | 12 | 5 | 0 | 13 |
| 6 | a | AAGGCG | 0 | 0 | 0 | 2 | 0 | 0 | 0 | 0 | 0 | 1 | 1 | 0 | 2 | 0 | 2 |
| 6 | A | AATAGT | 0 | 0 | 0 | 1 | 3 | 1 | 0 | 0 | 0 | 3 | 3 | 4 | 21 | 0 | 21 |
| 6 | B | ACTATT | 0 | 0 | 0 | 0 | 0 | 1 | 0 | 0 | 2 | 4 | 2 | 2 | 18 | 0 | 18 |
| 6 | a | AATCGG | 0 | 0 | 0 | 0 | 0 | 0 | 6 | 0 | 12 | 18 | 0 | 0 | 1 | 0 | 18 |
| 6 | a | AATGAC | 0 | 0 | 0 | 0 | 1 | 0 | 1 | 0 | 1 | 1 | 2 | 0 | 2 | 0 | 2 |
| 6 | a | AATGAT | 0 | 0 | 0 | 1 | 0 | 1 | 0 | 1 | 8 | 6 | 9 | 6 | 19 | 0 | 19 |
| 6 | A | ACAGAT | 0 | 0 | 0 | 0 | 0 | 4 | 8 | 2 | 150 | 99 | 0 | 1 | 1 | 0 | 150 |
| 6 | B | ATCTGT | 0 | 0 | 0 | 0 | 3 | 6 | 0 | 1 | 117 | 113 | 0 | 0 | 1 | 0 | 117 |
| 6 | b | CGGTCT | 0 | 0 | 0 | 0 | 1 | 0 | 0 | 0 | 0 | 2 | 1 | 0 | 4 | 0 | 4 |
| 6 | b | ATCGTG | 0 | 0 | 0 | 0 | 1 | 0 | 0 | 2 | 1 | 0 | 2 | 0 | 5 | 0 | 5 |
| 6 | a | ATCCGC | 0 | 0 | 0 | 0 | 0 | 2 | 6 | 13 | 19 | 29 | 0 | 0 | 0 | 0 | 29 |
| 7 | a | AAAAAAC | 1 | 0 | 1 | 21 | 43 | 8 | 2 | 0 | 21 | 24 | 0 | 1 | 20 | 0 | 43 |
| 7 | A | AAAAAAG | 0 | 0 | 0 | 36 | 88 | 0 | 2 | 0 | 5 | 2 | 2 | 45 | 533 | 0 | 533 |
| 7 | B | CTTTTTT | 0 | 0 | 0 | 40 | 63 | 0 | 0 | 0 | 4 | 0 | 1 | 62 | 616 | 0 | 616 |
| 7 | A | AATATAT | 0 | 0 | 0 | 2 | 2 | 0 | 0 | 0 | 18 | 28 | 0 | 41 | 558 | 0 | 558 |
| 7 | B | ATATATT | 0 | 0 | 0 | 4 | 5 | 2 | 0 | 0 | 9 | 23 | 0 | 43 | 530 | 0 | 530 |
| 8 | A | AAAAAAAG | 0 | 0 | 0 | 18 | 42 | 2 | 2 | 0 | 12 | 4 | 1 | 37 | 403 | 0 | 403 |
| 8 | B | CTTTTTTT | 0 | 0 | 0 | 21 | 50 | 0 | 0 | 0 | 0 | 3 | 0 | 43 | 386 | 0 | 386 |
| 8 | a | ACATATAT | 0 | 0 | 0 | 0 | 2 | 7 | 1 | 0 | 14 | 19 | 0 | 9 | 123 | 0 | 123 |
| 9 | b | CTTTTTTTT | 0 | 1 | 0 | 10 | 18 | 0 | 0 | 0 | 0 | 1 | 0 | 32 | 281 | 0 | 281 |

| | | | | | | | | | | | | | | | | | |
|---|---|---|---|---|---|---|---|---|---|---|---|---|---|---|---|---|---|
| 9 | a | AACAGCAGC | 0 | 0 | 4 | 2 | 5 | 2 | 3 | 53 | 13 | 14 | 1 | 0 | 18 | 0 | 53 |
| 9 | a | AATAATCAT | 0 | 0 | 0 | 0 | 1 | 0 | 0 | 0 | 3 | 1 | 0 | 1 | 4 | 0 | 4 |
| 9 | b | ACTACTATT | 0 | 0 | 0 | 0 | 1 | 0 | 0 | 0 | 0 | 0 | 2 | 0 | 4 | 0 | 4 |
| 9 | a | AATATATAT | 0 | 0 | 0 | 1 | 2 | 0 | 0 | 0 | 6 | 6 | 0 | 20 | 196 | 0 | 196 |
| 10 | a | AAAGCTCGAG | 0 | 0 | 0 | 0 | 0 | 0 | 0 | 0 | 0 | 0 | 1 | 0 | 0 | 0 | 1 |
| 10 | b | AAGGTTCGCC | 0 | 0 | 5 | 0 | 1 | 0 | 0 | 0 | 0 | 0 | 0 | 0 | 0 | 0 | 5 |
| 10 | b | ACTTGGATGG | 0 | 0 | 0 | 0 | 0 | 0 | 0 | 0 | 0 | 0 | 3 | 1 | 1 | 0 | 3 |
| 10 | b | ATCGTGGTCC | 0 | 0 | 0 | 0 | 0 | 0 | 0 | 0 | 0 | 0 | 2 | 0 | 0 | 0 | 2 |
| 10 | a | ACCATGGACT | 0 | 0 | 0 | 0 | 0 | 0 | 0 | 0 | 0 | 0 | 1 | 0 | 0 | 0 | 1 |
| 10 | b | ACGGTGGACT | 0 | 0 | 0 | 0 | 0 | 0 | 0 | 0 | 0 | 0 | 2 | 0 | 2 | 0 | 2 |
| 10 | a | ACGATGGACT | 0 | 0 | 0 | 0 | 0 | 0 | 0 | 0 | 0 | 0 | 2 | 1 | 4 | 0 | 4 |
| 10 | b | CGTGCTCTGG | 0 | 0 | 1 | 0 | 49 | 0 | 0 | 0 | 0 | 0 | 0 | 0 | 0 | 0 | 49 |
| 10 | A | ACGCCAGTGC | 0 | 0 | 5 | 0 | 3 | 0 | 0 | 0 | 0 | 0 | 0 | 0 | 0 | 0 | 5 |
| 10 | B | ACTGGCGTGC | 0 | 0 | 0 | 0 | 12 | 0 | 0 | 0 | 0 | 0 | 0 | 0 | 0 | 0 | 12 |
| 11 | a | AAACAGCACTC | 0 | 1 | 0 | 0 | 0 | 0 | 0 | 0 | 0 | 0 | 0 | 0 | 0 | 0 | 1 |
| 11 | A | ACCAGTACGGG | 0 | 0 | 0 | 0 | 0 | 0 | 0 | 0 | 1 | 55 | 0 | 0 | 0 | 0 | 55 |
| 11 | B | ACTGGTCCCGT | 0 | 0 | 0 | 0 | 0 | 0 | 0 | 2 | 5 | 57 | 0 | 0 | 0 | 0 | 57 |
| 12 | a | AACAGCAGCAGC | 0 | 0 | 3 | 3 | 2 | 1 | 0 | 32 | 6 | 5 | 1 | 0 | 4 | 0 | 32 |
| 12 | b | ATGCCTGGTTGC | 1 | 0 | 0 | 0 | 0 | 0 | 0 | 0 | 0 | 0 | 0 | 0 | 0 | 0 | 1 |
| 12 | a | AATGATGATGAT | 0 | 0 | 0 | 0 | 0 | 1 | 0 | 0 | 2 | 0 | 2 | 0 | 1 | 0 | 2 |
| 12 | A | ACCGAGTACGGG | 0 | 0 | 0 | 0 | 0 | 0 | 0 | 0 | 2 | 21 | 0 | 0 | 0 | 0 | 21 |
| 12 | B | ACTCGGTCCCGT | 0 | 0 | 0 | 0 | 0 | 0 | 0 | 0 | 4 | 22 | 0 | 0 | 0 | 0 | 22 |
| 12 | a | AGCAGCCTATCC | 0 | 1 | 0 | 0 | 0 | 0 | 0 | 0 | 0 | 0 | 0 | 0 | 0 | 0 | 1 |
| 15 | a | AAAAATAGTGGACCG | 0 | 0 | 0 | 0 | 0 | 0 | 0 | 0 | 0 | 0 | 1 | 0 | 0 | 0 | 1 |
| 15 | a | AAAAGTCTACTGATT | 0 | 1 | 0 | 0 | 0 | 0 | 0 | 0 | 0 | 0 | 0 | 0 | 0 | 0 | 1 |
| 15 | b | AACTTAGTTTATTTG | 0 | 0 | 0 | 0 | 0 | 0 | 0 | 0 | 0 | 0 | 4 | 2 | 0 | 0 | 4 |
| 15 | b | AACTTAGTTCATTTG | 0 | 0 | 0 | 0 | 0 | 0 | 0 | 0 | 0 | 0 | 2 | 0 | 0 | 0 | 2 |
| 15 | b | ACATTTGCTCCGCCG | 0 | 0 | 0 | 0 | 0 | 0 | 0 | 0 | 0 | 0 | 1 | 0 | 0 | 0 | 1 |
| 15 | a | AACCCAGCCTGACCC | 0 | 0 | 0 | 0 | 0 | 0 | 0 | 0 | 0 | 0 | 1 | 0 | 0 | 0 | 1 |
| 15 | a | AAGTCACGGAGGACG | 0 | 0 | 0 | 0 | 0 | 0 | 0 | 0 | 0 | 0 | 1 | 0 | 0 | 0 | 1 |
| 15 | b | ACGATGGTGTCGATG | 0 | 0 | 0 | 0 | 0 | 0 | 0 | 0 | 0 | 0 | 1 | 0 | 0 | 0 | 1 |
| 16 | a | AAAAATCTCAAAGACC | 0 | 0 | 0 | 0 | 0 | 0 | 0 | 0 | 0 | 0 | 1 | 0 | 0 | 0 | 1 |
| 16 | a | AAATTCGAAGGCATCC | 0 | 0 | 0 | 0 | 0 | 0 | 0 | 0 | 0 | 0 | 1 | 0 | 0 | 0 | 1 |
| 17 | A | AAAAGTAGAACTTTTCT | 0 | 0 | 0 | 0 | 30 | 0 | 0 | 0 | 0 | 0 | 0 | 0 | 0 | 0 | 30 |
| 17 | B | AAAAGTTCTACTTTTAG | 0 | 0 | 0 | 0 | 15 | 0 | 0 | 0 | 0 | 0 | 0 | 0 | 0 | 0 | 15 |
| 17 | A | AAAAGTTCAACTTTATG | 0 | 0 | 0 | 0 | 53 | 0 | 0 | 0 | 0 | 0 | 0 | 0 | 0 | 0 | 53 |
| 17 | B | AAAGTTGAACTTTTCAT | 0 | 0 | 0 | 0 | 82 | 0 | 0 | 0 | 0 | 0 | 0 | 0 | 0 | 0 | 82 |
| 17 | b | AAAGTAGAACTTTTGTC | 0 | 0 | 0 | 0 | 15 | 0 | 0 | 0 | 0 | 0 | 0 | 0 | 0 | 0 | 15 |
| 18 | a | AAAGAAGAAGAGAAGAGG | 0 | 0 | 0 | 0 | 0 | 0 | 0 | 1 | 0 | 0 | 0 | 0 | 0 | 0 | 1 |
| 18 | a | AAATTTCAAGGAACGTCG | 0 | 0 | 0 | 0 | 0 | 0 | 0 | 0 | 0 | 0 | 1 | 0 | 0 | 0 | 1 |
| 18 | b | ACGGTGCTGGACTCGGTT | 0 | 1 | 0 | 0 | 0 | 0 | 0 | 0 | 0 | 0 | 0 | 0 | 0 | 0 | 1 |
| 18 | a | AACGTGAACGTGATCGTG | 0 | 0 | 0 | 0 | 0 | 0 | 0 | 0 | 0 | 0 | 1 | 0 | 0 | 0 | 1 |
| 19 | a | AAGAAGTAAGAATAAGAGT | 0 | 0 | 0 | 0 | 0 | 0 | 0 | 0 | 0 | 0 | 1 | 0 | 0 | 0 | 1 |
| 20 | b | ACGTCCATCGTGGTCCATCT | 0 | 0 | 0 | 0 | 0 | 0 | 0 | 0 | 0 | 0 | 1 | 0 | 0 | 0 | 1 |
| 21 | a | AAAATGATGGAAATCAGGACG | 0 | 0 | 0 | 0 | 0 | 0 | 0 | 0 | 0 | 0 | 3 | 0 | 0 | 0 | 3 |
| 21 | a | AATAATAATAATGATAATGAT | 0 | 0 | 0 | 0 | 0 | 0 | 0 | 0 | 0 | 0 | 1 | 0 | 0 | 0 | 1 |
| 21 | a | AATAGTAGTAGTAGTAGTAGT | 0 | 0 | 0 | 0 | 0 | 0 | 0 | 0 | 0 | 0 | 1 | 0 | 0 | 0 | 1 |
| 21 | b | ACTGGTCCCGTACTGGTCCGT | 0 | 0 | 0 | 0 | 0 | 0 | 0 | 0 | 0 | 1 | 0 | 0 | 0 | 0 | 1 |
| 22 | b | AACTACTACATTTATTACTTTT | 1 | 0 | 0 | 0 | 0 | 0 | 0 | 0 | 0 | 0 | 0 | 0 | 0 | 0 | 1 |
| 22 | a | AAAGAATCGTCGAAGAATCGTC | 0 | 0 | 0 | 0 | 0 | 0 | 0 | 0 | 0 | 0 | 1 | 0 | 0 | 0 | 1 |
| 22 | a | ACCAGTACGGGACGAGTACGGG | 0 | 0 | 0 | 0 | 0 | 0 | 0 | 0 | 0 | 1 | 0 | 0 | 0 | 0 | 1 |
| 23 | a | ACCAGTACGGGACCCAGTACGGG | 0 | 0 | 0 | 0 | 0 | 0 | 0 | 0 | 0 | 1 | 0 | 0 | 0 | 0 | 1 |
| 23 | A | ACCAGTACGGGACCGAGTACGGG | 0 | 0 | 0 | 0 | 0 | 0 | 0 | 0 | 0 | 3 | 0 | 0 | 0 | 0 | 3 |
| 23 | B | ACTCGGTCCCGTACTGGTCCCGT | 0 | 0 | 0 | 0 | 0 | 0 | 0 | 0 | 1 | 4 | 0 | 0 | 0 | 0 | 4 |
| 24 | b | AAATTTTAGTTATATTTGAAGTTC | 0 | 0 | 0 | 0 | 0 | 0 | 0 | 0 | 0 | 0 | 3 | 1 | 1 | 0 | 3 |
| 24 | b | CGGTGTTGTCTTGTCTGTTGGCTT | 0 | 1 | 0 | 0 | 0 | 0 | 0 | 0 | 0 | 0 | 0 | 0 | 0 | 0 | 1 |
| 24 | a | AAGGCAGCGGAGTACTACACCACC | 0 | 0 | 10 | 3 | 8 | 0 | 0 | 0 | 0 | 0 | 0 | 0 | 0 | 0 | 10 |
| 24 | a | ACCGTCTGCTCCGCCGGCCCAGCC | 0 | 0 | 13 | 1 | 5 | 0 | 0 | 0 | 0 | 0 | 0 | 0 | 0 | 0 | 13 |
| 26 | b | AAATTTCCGTGTGAAGAGGAATTGAT | 0 | 0 | 0 | 0 | 0 | 0 | 0 | 0 | 0 | 0 | 0 | 3 | 4 | 0 | 4 |
| 28 | a | AAATAACTTATCAATTCCTCCTCGGACG | 0 | 0 | 0 | 0 | 0 | 0 | 0 | 0 | 0 | 0 | 0 | 1 | 0 | 0 | 1 |
| 30 | b | AAATACGTGCGTTTAGAAATTCCAGAAATC | 0 | 0 | 0 | 0 | 0 | 0 | 0 | 0 | 0 | 0 | 1 | 0 | 0 | 0 | 1 |
| 30 | b | ATGATGATGATGATGATGATGATGATGATT | 0 | 0 | 0 | 0 | 0 | 0 | 0 | 0 | 0 | 0 | 1 | 0 | 0 | 0 | 1 |
| 30 | b | AGTCCATCGTAGTCCATCTATGTCCATCGT | 0 | 0 | 0 | 0 | 0 | 0 | 0 | 0 | 0 | 0 | 2 | 0 | 0 | 0 | 2 |
| 30 | a | ACATAGATGGACTACGATGGGCTACGATGG | 0 | 0 | 0 | 0 | 0 | 0 | 0 | 0 | 0 | 0 | 1 | 0 | 0 | 0 | 1 |
| 31 | a | AAAAATAAATTATTTCGTTTGCCCACCCTTT | 0 | 0 | 0 | 0 | 0 | 0 | 0 | 0 | 10 | 5 | 0 | 0 | 0 | 0 | 10 |
| 31 | b | AAACGAAATAATTTAATTTTAAAGGGTGGGC | 0 | 0 | 0 | 0 | 0 | 0 | 0 | 0 | 4 | 2 | 0 | 0 | 0 | 0 | 4 |
| 31 | a | AAGTAGTAGTAGTAGTAGTAGTAGTAGTAGT | 0 | 0 | 0 | 0 | 0 | 0 | 0 | 0 | 0 | 0 | 1 | 0 | 0 | 0 | 1 |
| 33 | b | ACTCGGTCCCGTACTGGTCCCGTACTGGTCCGT | 0 | 0 | 0 | 0 | 0 | 0 | 0 | 0 | 0 | 1 | 0 | 0 | 0 | 0 | 1 |
| 34 | b | ATCGTTCCTGTGGCTTTATGCCGGCTTCGTGCCC | 0 | 0 | 3 | 0 | 1 | 0 | 0 | 0 | 0 | 0 | 0 | 0 | 0 | 0 | 3 |
| 34 | b | ACTCGGTCCCGTACTGGTCCCGTACTGGTCCCGT | 0 | 0 | 0 | 0 | 0 | 0 | 0 | 0 | 0 | 2 | 0 | 0 | 0 | 0 | 2 |
| 35 | b | ACTCGGTCCCGTACTCGGTCCCGTACTGGTCCCGT | 0 | 0 | 0 | 0 | 0 | 0 | 0 | 0 | 1 | 1 | 0 | 0 | 0 | 0 | 1 |
| 36 | b | AAATTGAAGTTACAATTTTTGTTATATTTGAAGTTC | 0 | 0 | 0 | 0 | 0 | 0 | 0 | 0 | 0 | 0 | 2 | 0 | 1 | 0 | 2 |
| 36 | b | AAAATTTTTGTTATATTTGAAGTTCAAGTTGAAGTT | 0 | 0 | 0 | 0 | 0 | 0 | 0 | 0 | 0 | 0 | 1 | 0 | 0 | 0 | 1 |
| 36 | b | AAAATTTTTGTTATATCTGAAGTTCAAATTGAAGTT | 0 | 0 | 0 | 0 | 0 | 0 | 0 | 0 | 0 | 0 | 2 | 6 | 5 | 0 | 6 |
| 36 | b | AAAATTTTTGTTATATCTGAAGTTCAAATGGAAGTT | 0 | 0 | 0 | 0 | 0 | 0 | 0 | 0 | 0 | 0 | 2 | 0 | 1 | 0 | 2 |
| 36 | b | ACCTTCTCCGTCCTTTCTCTCCTCAGCTTCATCTTC | 1 | 0 | 0 | 0 | 0 | 0 | 0 | 0 | 0 | 0 | 0 | 0 | 0 | 0 | 1 |
| 39 | a | AAACCAACTGAGGGAACGAGTGCCAAGCCAACAACTTTG | 0 | 0 | 0 | 0 | 0 | 0 | 0 | 1 | 0 | 0 | 0 | 0 | 0 | 0 | 1 |
| 45 | b | AAATATTCCAAGTATCAAGATCGAAGTAAGTTTGAGTAGATTGAG | 0 | 0 | 0 | 0 | 0 | 0 | 0 | 0 | 0 | 0 | 1 | 0 | 0 | 0 | 1 |
| 45 | a | AACGACGACAACGTCAACGACGACAACGTCAACGCCGACACCGAC | 0 | 0 | 0 | 0 | 0 | 0 | 0 | 0 | 0 | 0 | 1 | 0 | 0 | 0 | 1 |
| 45 | a | ACCAGTACGGGACCAGTACGGGACCAGTACGGGACCGAGTACGGG | 0 | 0 | 0 | 0 | 0 | 0 | 0 | 0 | 0 | 2 | 0 | 0 | 0 | 0 | 2 |
| 46 | a | ACCAGTACGGGACCAGTACGGGACCGAGTACGGGACCGAGTACGGG | 0 | 0 | 0 | 0 | 0 | 0 | 0 | 0 | 0 | 2 | 0 | 0 | 0 | 0 | 2 |
| 47 | b | AACACTCTTCCTTCTCGGATGGATTTGAGCAGTTGGAACGTGAAGAG | 0 | 0 | 2 | 0 | 0 | 0 | 0 | 0 | 0 | 0 | 0 | 0 | 0 | 0 | 2 |
| 47 | b | AACACTCTTCTCTTCCGGATGGATTTGAGCAGTTGGAACGTGAAGAG | 0 | 0 | 1 | 0 | 0 | 0 | 0 | 0 | 0 | 0 | 0 | 0 | 0 | 0 | 1 |
| 48 | a | AAAAATGTCAAAGATTGTTGTATAAGGGATATAATTGATTATTGTAAG | 0 | 0 | 0 | 0 | 0 | 0 | 0 | 0 | 0 | 0 | 1 | 0 | 0 | 0 | 1 |
| 48 | a | AACAACAACTTCCACAAGTGGCCCAACGACAACTACTCCTAGATCCAC | 0 | 0 | 0 | 0 | 0 | 0 | 0 | 1 | 0 | 0 | 0 | 0 | 0 | 0 | 1 |
| 48 | a | ACCGAGTACGGGACCGAGTACGGGACCGAGTACGGGACCTAGTACGGG | 0 | 0 | 0 | 0 | 0 | 0 | 0 | 0 | 0 | 1 | 0 | 0 | 0 | 0 | 1 |

**Additional file 7:** (d) Mean perfection of repeat motifs in different genomic regions of *Daphnia pulex* (Dappu), *Drosophila melanogaster* (DroMel) and *Apis mellifera* (ApiMel). A=normal B=reverse complement of repeat motif A; "-" denotes palindromic motifs; a and b are used if only the normal form (a) OR its reverse complement (b) were found.

| unit size [bp] | motif form | unit | Dappu v1.1 3UTR [%] | Dappu v1.1 5UTR [%] | Dappu v1.1 CDS [%] | Dappu v1.1 introns [%] | Dappu v1.1 intergenic [%] | DroMel-5.5 3UTR [%] | DroMel-5.5 5UTR [%] | DroMel-5.5 CDS [%] | DroMel-5.5 introns [%] | DroMel-5.5 intergenic [%] | ApiMel-2.0 CDS [%] | ApiMel-2.0 introns [%] | ApiMel-2.0 intergenic [%] | min [%] | max [%] |
|---|---|---|---|---|---|---|---|---|---|---|---|---|---|---|---|---|---|
| 1 | A | A | 99.3 | 99.6 | 98.6 | 99.4 | 99.3 | 99.5 | 99.3 | 99.4 | 99.3 | 99.4 | 98.9 | 99.0 | 99.0 | 98.6 | 99.6 |
| 1 | B | T | 99.2 | 100.0 | 98.4 | 99.3 | 99.2 | 99.3 | 99.7 | 99.0 | 99.4 | 99.4 | 98.3 | 99.0 | 99.1 | 98.3 | 100.0 |
| 1 | A | C | 100.0 | 100.0 | 100.0 | 99.9 | 99.9 | 100.0 | 100.0 |  | 99.9 | 99.9 | 100.0 | 100.0 | 99.8 | 99.8 | 100.0 |
| 1 | B | G | 99.7 | 100.0 | 100.0 | 99.9 | 99.9 | 100.0 |  |  | 99.9 | 99.9 | 100.0 | 99.8 | 99.8 | 99.7 | 100.0 |
| 2 | A | AC | 99.3 | 100.0 | 100.0 | 99.5 | 99.4 | 98.7 | 98.6 | 100.0 | 98.9 | 98.9 | 100.0 | 99.1 | 99.1 | 98.6 | 100.0 |
| 2 | B | GT | 99.8 | 97.5 | 98.5 | 99.5 | 99.4 | 98.2 | 99.2 | 99.2 | 99.0 | 98.9 |  | 98.6 | 98.9 | 97.5 | 99.8 |
| 2 | A | AG | 100.0 | 100.0 | 99.6 | 99.5 | 99.5 | 98.3 | 98.9 | 98.2 | 98.5 | 98.9 | 98.4 | 98.8 | 98.7 | 98.2 | 100.0 |
| 2 | B | CT | 99.6 | 99.3 | 99.0 | 99.5 | 99.5 | 99.1 | 98.8 | 100.0 | 98.7 | 98.7 | 98.0 | 98.5 | 98.7 | 98.0 | 100.0 |
| 2 | - | AT | 98.6 | 100.0 | 100.0 | 99.2 | 98.9 | 97.9 | 97.8 | 98.7 | 98.0 | 97.9 | 95.5 | 98.5 | 98.5 | 95.5 | 100.0 |
| 3 | A | AAC | 99.4 | 97.8 | 98.8 | 99.0 | 99.3 | 97.6 | 98.6 | 98.5 | 98.2 | 98.7 | 97.9 | 99.4 | 98.9 | 97.6 | 99.4 |
| 3 | B | GTT | 99.3 | 96.8 | 99.3 | 99.4 | 99.2 | 98.5 | 98.9 | 95.8 | 98.6 | 98.5 | 98.4 | 99.1 | 98.7 | 95.8 | 99.4 |
| 3 | A | AAG | 99.4 | 100.0 | 98.7 | 99.5 | 99.4 | 95.2 | 99.5 | 98.5 | 99.4 | 99.6 | 98.2 | 99.0 | 98.7 | 95.2 | 100.0 |
| 3 | B | CTT | 98.7 |  | 99.1 | 99.4 | 99.4 | 100.0 | 95.2 | 95.2 | 99.1 | 99.2 | 98.9 | 98.7 | 98.7 | 95.2 | 100.0 |
| 3 | A | AAT | 100.0 | 100.0 | 99.0 | 99.1 | 99.4 | 98.1 | 98.5 | 98.8 | 98.1 | 97.5 | 96.7 | 97.8 | 98.3 | 96.7 | 100.0 |
| 3 | B | ATT | 100.0 | 100.0 | 98.1 | 99.2 | 99.3 | 98.1 | 98.2 | 100.0 | 98.1 | 97.3 | 97.9 | 98.5 | 98.3 | 97.3 | 100.0 |
| 3 | A | ACC | 98.3 | 100.0 | 99.4 | 99.9 | 99.5 | 98.0 | 98.8 | 98.4 | 99.0 | 99.0 | 97.2 | 97.8 | 98.4 | 97.2 | 100.0 |
| 3 | B | GGT |  | 100.0 | 99.5 | 99.5 | 99.5 | 95.5 | 97.5 | 98.8 | 99.0 | 98.6 | 96.9 | 99.6 | 98.3 | 95.5 | 100.0 |
| 3 | A | ACG | 100.0 |  | 99.2 | 99.7 | 99.6 | 98.4 | 99.3 | 98.9 | 99.2 | 98.6 | 96.9 | 99.2 | 98.3 | 96.9 | 100.0 |
| 3 | B | CGT |  | 100.0 | 99.5 | 99.7 | 99.6 | 100.0 | 98.7 | 99.0 | 99.5 | 99.2 | 93.7 | 98.8 | 98.3 | 93.7 | 100.0 |
| 3 | A | ACT | 97.3 | 96.7 | 98.9 | 98.8 | 99.0 | 98.0 | 100.0 | 97.9 | 98.6 | 98.8 | 93.1 | 96.5 | 97.3 | 93.1 | 100.0 |
| 3 | B | AGT |  |  | 98.6 | 99.0 | 99.2 | 96.2 |  | 98.2 | 98.9 | 98.3 | 95.1 | 96.6 | 97.5 | 95.1 | 99.2 |
| 3 | A | AGC | 100.0 | 98.5 | 99.2 | 99.6 | 99.6 | 98.2 | 97.7 | 97.4 | 97.7 | 97.3 | 97.6 | 97.8 | 98.2 | 97.3 | 100.0 |
| 3 | B | CTG | 100.0 | 100.0 | 99.5 | 99.6 | 99.5 | 100.0 | 98.7 | 98.7 | 98.4 | 98.0 | 98.6 | 100.0 | 98.0 | 98.0 | 100.0 |
| 3 | A | AGG |  |  | 99.4 | 99.6 | 99.5 | 98.7 | 99.2 | 98.2 | 98.4 | 98.8 | 98.0 | 99.0 | 98.9 | 98.0 | 99.6 |
| 3 | B | CCT | 100.0 | 100.0 | 99.1 | 99.7 | 99.5 | 99.0 | 100.0 | 99.4 | 98.4 | 98.3 | 99.1 | 99.0 | 98.8 | 98.3 | 100.0 |
| 3 | A | ATC | 100.0 | 100.0 | 99.5 | 99.6 | 99.5 | 98.4 | 99.1 | 98.6 | 98.5 | 98.9 | 98.4 | 99.5 | 98.7 | 98.4 | 100.0 |
| 3 | B | ATG | 97.7 |  | 98.6 | 99.8 | 99.5 | 98.0 | 100.0 | 99.1 | 98.8 | 98.7 | 97.1 | 97.8 | 98.5 | 97.1 | 100.0 |
| 3 | A | CCG | 100.0 |  | 98.7 | 99.5 | 99.3 | 100.0 | 99.0 | 99.2 | 100.0 | 99.6 | 98.7 | 99.5 | 99.2 | 98.7 | 100.0 |
| 3 | B | CGG |  |  | 98.9 | 99.4 | 99.4 | 98.5 | 100.0 | 98.1 | 99.7 | 99.7 | 98.7 | 99.7 | 99.1 | 98.1 | 100.0 |
| 4 | A | AAAC | 98.9 | 100.0 | 97.6 | 99.2 | 98.7 | 99.3 | 98.7 |  | 99.0 | 98.8 |  | 99.2 | 98.9 | 97.6 | 100.0 |
| 4 | B | GTTT | 100.0 |  | 98.8 | 98.8 | 98.6 | 95.7 | 98.1 |  | 98.7 | 99.0 |  | 100.0 | 99.5 | 95.7 | 100.0 |
| 4 | A | AAAG | 95.7 |  | 100.0 | 99.1 | 98.7 | 99.4 | 98.0 |  | 99.5 | 99.5 | 98.5 | 98.1 | 98.5 | 95.7 | 100.0 |
| 4 | B | CTTT | 100.0 | 95.5 | 100.0 | 98.9 | 98.9 |  |  |  | 98.9 | 97.2 | 100.0 | 98.2 | 98.5 | 95.5 | 100.0 |
| 4 | A | AAAT | 97.5 |  |  | 98.6 | 99.0 | 98.5 | 98.9 |  | 99.2 | 98.7 |  | 98.3 | 98.5 | 97.5 | 99.2 |
| 4 | B | ATTT | 100.0 |  | 100.0 | 99.0 | 98.9 | 99.2 | 98.0 |  | 99.2 | 98.1 | 100.0 | 98.8 | 98.5 | 98.0 | 100.0 |
| 4 | a | AACC | 100.0 |  |  | 99.1 | 99.4 | 97.9 | 99.3 | 100.0 | 98.6 | 98.6 |  | 100.0 | 98.9 | 97.9 | 100.0 |
| 4 | A | AAGG |  |  | 97.7 | 100.0 | 99.1 | 100.0 |  |  | 99.3 | 98.1 | 100.0 | 98.9 | 99.1 | 97.7 | 100.0 |
| 4 | B | CCTT |  |  | 100.0 | 99.5 | 99.4 |  |  |  | 98.2 | 99.4 |  | 99.4 | 99.0 | 98.2 | 100.0 |
| 4 | - | AATT |  |  |  | 98.4 | 99.7 | 98.9 | 97.7 |  | 98.9 | 97.8 |  | 98.7 | 98.6 | 97.7 | 99.7 |
| 4 | A | ACAT |  |  | 100.0 | 98.4 | 98.7 | 99.4 | 97.9 |  | 99.2 | 99.0 |  | 97.8 | 98.4 | 97.8 | 100.0 |
| 4 | B | ATGT |  |  |  | 99.6 | 99.0 | 99.8 | 100.0 |  | 99.1 | 99.1 |  | 96.9 | 98.4 | 96.9 | 100.0 |
| 4 | a | AGCC |  | 100.0 | 100.0 | 99.5 | 99.5 | 99.4 | 97.3 | 100.0 | 98.6 | 98.6 |  | 98.4 | 98.8 | 97.3 | 100.0 |
| 4 | A | AGGG |  |  |  | 98.8 | 99.8 |  |  |  | 100.0 | 96.0 |  | 99.2 | 99.2 | 96.0 | 100.0 |
| 4 | B | CCCT |  |  |  | 99.5 | 99.7 |  |  |  | 98.6 | 94.1 | 100.0 | 99.4 | 99.1 | 94.1 | 100.0 |
| 4 | a | ATCC | 100.0 |  | 100.0 | 99.2 | 99.3 | 100.0 |  |  | 98.1 | 97.6 |  | 99.0 | 99.3 | 97.6 | 100.0 |
| 5 | A | AAAAC | 98.5 |  | 100.0 | 99.0 | 98.4 | 98.3 | 94.9 |  | 98.1 | 98.8 |  | 100.0 | 98.9 | 94.9 | 100.0 |
| 5 | B | GTTTT | 100.0 | 100.0 | 100.0 | 99.5 | 98.8 |  | 95.7 |  | 98.5 | 98.9 |  | 100.0 | 99.6 | 95.7 | 100.0 |
| 5 | A | AAAAG | 100.0 | 100.0 | 98.2 | 99.0 | 98.6 | 100.0 | 100.0 |  | 96.8 | 99.1 | 97.8 | 97.6 | 98.0 | 96.8 | 100.0 |
| 5 | B | CTTTT | 98.0 | 95.8 |  | 98.9 | 98.9 | 95.8 |  |  | 98.1 | 98.6 | 89.8 | 97.7 | 98.0 | 89.8 | 98.9 |
| 5 | A | AAAAT | 100.0 | 98.0 |  | 99.3 | 99.0 | 99.0 | 96.5 |  | 97.8 | 98.2 |  | 98.2 | 98.4 | 96.5 | 100.0 |
| 5 | B | ATTTT | 97.8 | 100.0 |  | 99.0 | 99.1 | 98.6 |  |  | 97.8 | 98.3 |  | 98.3 | 98.4 | 97.8 | 100.0 |
| 5 | A | AACCT |  |  |  | 98.9 | 98.9 |  |  |  |  |  |  | 98.0 | 98.7 | 98.0 | 98.9 |
| 5 | B | AGGTT |  |  |  | 98.1 | 98.2 |  |  |  |  |  |  | 98.3 | 98.8 | 98.1 | 98.8 |
| 5 | A | AAGAT |  |  |  | 97.4 | 98.0 |  |  |  | 100.0 | 97.9 |  | 100.0 | 99.4 | 97.4 | 100.0 |
| 5 | B | ATCTT |  |  |  | 98.5 | 98.3 |  |  |  | 99.1 | 99.2 |  | 100.0 | 98.9 | 98.3 | 100.0 |
| 5 | A | AATAG |  |  |  | 96.4 | 98.8 |  | 100.0 |  | 98.3 | 96.4 | 95.5 | 98.9 | 98.8 | 95.5 | 100.0 |
| 5 | B | ATTCT |  |  |  | 99.4 | 99.2 |  | 100.0 |  | 98.1 | 96.5 |  | 97.4 | 98.6 | 96.5 | 100.0 |
| 5 | A | AATAT |  |  |  | 100.0 | 97.8 | 96.3 |  |  | 96.8 | 96.8 |  | 96.3 | 96.4 | 96.3 | 100.0 |
| 5 | B | ATATT | 100.0 |  |  | 98.2 | 97.8 | 93.7 | 90.9 |  | 97.1 | 97.6 | 100.0 | 97.0 | 96.8 | 90.9 | 100.0 |
| 5 | A | AATCG |  |  |  | 100.0 |  | 94.2 | 97.1 |  | 97.6 | 98.5 |  | 99.6 | 99.5 | 94.2 | 100.0 |
| 5 | B | ATTCG |  |  |  |  | 100.0 |  | 96.9 | 100.0 | 97.7 | 97.4 |  | 100.0 | 99.3 | 96.9 | 100.0 |
| 5 | A | ATCCG |  |  | 100.0 |  | 100.0 | 97.9 | 98.3 | 100.0 | 98.9 | 98.5 |  | 100.0 | 99.7 | 97.9 | 100.0 |
| 5 | B | ATCGG |  |  |  |  | 100.0 | 97.9 | 98.2 | 100.0 | 98.1 | 98.1 |  |  | 98.3 | 97.9 | 100.0 |
| 6 | a | AAAAAC | 100.0 | 100.0 | 100.0 | 98.1 | 97.8 | 95.3 |  |  | 98.1 | 97.9 |  |  | 98.8 | 95.3 | 100.0 |
| 6 | A | AAAAAG | 98.0 |  | 96.5 | 98.0 | 98.3 | 100.0 |  | 100.0 | 96.8 | 97.5 | 97.1 | 96.5 | 97.2 | 96.5 | 100.0 |
| 6 | B | CTTTTT | 97.9 |  |  | 97.4 | 97.7 | 100.0 |  |  | 98.8 | 97.6 |  | 96.9 | 97.4 | 96.9 | 100.0 |
| 6 | b | ATTTTT | 100.0 |  |  | 98.6 | 99.0 | 95.8 |  |  | 98.1 | 97.6 |  | 98.1 | 98.2 | 95.8 | 100.0 |
| 6 | a | AAAAGC |  |  | 96.6 |  | 99.1 | 95.4 | 97.6 |  | 97.9 | 97.9 |  |  | 100.0 | 95.4 | 100.0 |
| 6 | a | AAATAC |  |  |  |  | 97.3 | 100.0 | 100.0 |  | 98.2 | 98.2 | 100.0 |  | 99.3 | 97.3 | 100.0 |
| 6 | A | AAATAT |  |  |  | 97.9 | 92.2 | 98.2 | 100.0 |  | 99.0 | 98.1 |  | 97.5 | 97.3 | 92.2 | 100.0 |
| 6 | B | ATATTT |  |  |  | 96.7 | 99.0 | 100.0 | 100.0 |  | 99.3 | 98.0 |  | 97.1 | 97.8 | 96.7 | 100.0 |
| 6 | a | AACAAT |  |  |  |  | 98.6 |  |  | 98.5 | 99.4 | 97.7 | 94.9 | 94.1 | 99.0 | 94.1 | 99.4 |
| 6 | a | AACAGC |  |  | 96.5 | 93.5 | 99.0 | 97.2 | 97.1 | 96.4 | 97.9 | 97.8 | 98.7 |  | 96.4 | 93.5 | 99.0 |
| 6 | A | AACCCC |  |  |  |  | 99.4 |  |  |  |  | 98.2 |  |  |  | 98.2 | 99.4 |
| 6 | B | GGGGTT |  |  |  | 100.0 | 99.6 |  |  |  | 100.0 | 100.0 |  |  |  | 99.6 | 100.0 |
| 6 | a | AAGAGT |  |  |  |  |  |  |  |  |  |  | 92.4 |  | 100.0 | 92.4 | 100.0 |
| 6 | a | AAGCAG |  |  | 100.0 | 100.0 | 100.0 | 98.7 | 100.0 | 100.0 | 100.0 | 98.0 | 91.4 | 94.0 | 94.3 | 91.4 | 100.0 |
| 6 | a | AAGGCG |  |  |  | 100.0 |  |  |  |  |  | 94.1 | 100.0 |  | 100.0 | 94.1 | 100.0 |
| 6 | A | AATAGT |  |  |  | 100.0 | 97.5 | 100.0 |  |  |  | 96.0 | 93.8 | 96.4 | 98.2 | 93.8 | 100.0 |
| 6 | B | ACTATT |  |  |  |  |  | 100.0 |  |  | 100.0 | 97.6 | 90.0 | 96.7 | 97.1 | 90.0 | 100.0 |
| 6 | a | AATCGG |  |  |  |  |  |  | 98.1 |  | 98.4 | 98.5 |  |  | 100.0 | 98.1 | 100.0 |
| 6 | a | AATGAC |  |  |  |  | 100.0 |  | 100.0 |  | 96.2 | 93.9 | 98.1 |  | 93.6 | 93.6 | 100.0 |
| 6 | a | AATGAT |  |  |  | 96.2 |  | 100.0 |  | 97.3 | 99.6 | 98.4 | 97.7 | 96.4 | 98.0 | 96.2 | 100.0 |
| 6 | A | ACAGAT |  |  |  |  |  | 95.8 | 97.1 | 97.8 | 97.5 | 98.1 |  | 100.0 | 100.0 | 95.8 | 100.0 |
| 6 | B | ATCTGT |  |  |  |  | 97.2 | 96.4 |  | 100.0 | 97.6 | 97.7 |  |  | 95.8 | 95.8 | 100.0 |
| 6 | b | CGGTCT |  |  |  |  | 100.0 |  |  |  |  | 100.0 | 90.7 |  | 100.0 | 90.7 | 100.0 |
| 6 | b | ATCGTG |  |  |  |  | 96.2 |  |  | 98.3 | 100.0 |  | 90.7 |  | 96.0 | 90.7 | 100.0 |
| 6 | a | ATCCGC |  |  |  |  |  | 96.6 | 98.6 | 97.8 | 97.5 | 97.9 |  |  |  | 96.6 | 98.6 |
| 7 | a | AAAAAAC | 100.0 |  | 100.0 | 98.7 | 98.0 | 97.7 | 100.0 |  | 97.6 | 98.2 |  | 94.1 | 98.1 | 94.1 | 100.0 |
| 7 | A | AAAAAAG |  |  |  | 98.4 | 98.5 |  | 96.0 |  | 95.7 | 100.0 | 100.0 | 97.4 | 97.5 | 95.7 | 100.0 |
| 7 | B | CTTTTTT |  |  |  | 98.2 | 98.3 |  |  |  | 97.9 |  | 96.2 | 97.6 | 97.4 | 96.2 | 98.3 |
| 7 | A | AATATAT |  |  |  | 95.8 | 93.5 |  |  |  | 93.2 | 95.6 |  | 95.3 | 96.0 | 93.2 | 96.0 |
| 7 | B | ATATATT |  |  |  | 98.2 | 97.5 | 100.0 |  |  | 95.4 | 97.5 |  | 95.9 | 95.8 | 95.4 | 100.0 |
| 8 | A | AAAAAAAG |  |  |  | 98.7 | 98.6 | 100.0 | 100.0 |  | 99.1 | 99.1 | 100.0 | 96.6 | 97.3 | 96.6 | 100.0 |
| 8 | B | CTTTTTTT |  |  |  | 98.6 | 99.2 |  |  |  |  | 98.9 |  | 97.8 | 97.6 | 97.6 | 99.2 |
| 8 | a | ACATATAT |  |  |  |  | 95.0 | 98.1 | 100.0 |  | 97.6 | 97.4 |  | 96.5 | 97.7 | 95.0 | 100.0 |
| 9 | b | CTTTTTTTT |  | 92.3 |  | 99.0 | 98.9 |  |  |  |  | 96.3 |  | 97.7 | 97.7 | 92.3 | 99.0 |
| 9 | a | AACAGCAGC |  |  | 97.3 | 98.2 | 98.2 | 97.3 | 94.3 | 96.5 | 96.6 | 95.6 | 92.0 |  | 95.2 | 92.0 | 98.2 |
| 9 | a | AATAATCAT |  |  |  |  | 100.0 |  |  |  | 94.1 | 96.3 |  | 100.0 | 99.1 | 94.1 | 100.0 |
| 9 | b | ACTACTATT |  |  |  |  | 100.0 |  |  |  |  |  | 92.8 |  | 96.1 | 92.8 | 100.0 |
| 9 | a | AATATATAT |  |  |  | 94.3 | 95.2 |  |  |  | 94.6 | 94.7 |  | 93.7 | 95.4 | 93.7 | 95.4 |

| | | | | | | | | | | | | | | | | |
|---|---|---|---|---|---|---|---|---|---|---|---|---|---|---|---|---|
| 10 | a | AAAGCTCGAG | | | | | | | | | | 88.6 | | | 88.6 | 88.6 |
| 10 | b | AAGGTTCGCC | | | 99.6 | | 100.0 | | | | | | | | 99.6 | 100.0 |
| 10 | b | ACTTGGATGG | | | | | | | | | | 94.3 | 97.5 | 97.4 | 94.3 | 97.5 |
| 10 | b | ATCGTGGTCC | | | | | | | | | | 94.1 | | | 94.1 | 94.1 |
| 10 | a | ACCATGGACT | | | | | | | | | | 91.4 | | | 91.4 | 91.4 |
| 10 | b | ACGGTGGACT | | | | | | | | | | 95.4 | | 97.3 | 95.4 | 97.3 |
| 10 | a | ACGATGGACT | | | | | | | | | | 91.6 | 86.4 | 89.5 | 86.4 | 91.6 |
| 10 | b | CGTGCTCTGG | | | 89.1 | | 94.8 | | | | | | | | 89.1 | 94.8 |
| 10 | A | ACGCCAGTGC | | | 90.3 | | 91.2 | | | | | | | | 90.3 | 91.2 |
| 10 | B | ACTGGCGTGC | | | | | 90.5 | | | | | | | | 90.5 | 90.5 |
| 11 | a | AAACAGCACTC | | 100.0 | | | | | | | | | | | 100.0 | 100.0 |
| 11 | A | ACCAGTACGGG | | | | | | | | 94.1 | 90.8 | | | | 90.8 | 94.1 |
| 11 | B | ACTGGTCCCGT | | | | | | | 88.5 | 95.1 | 90.7 | | | | 88.5 | 95.1 |
| 12 | a | AACAGCAGCAGC | | | 100.0 | 95.8 | 100.0 | 97.0 | 95.7 | 94.0 | 95.4 | 100.0 | | 97.8 | 94.0 | 100.0 |
| 12 | b | ATGCCTGGTTGC | 96.0 | | | | | | | | | | | | 96.0 | 96.0 |
| 12 | a | AATGATGATGAT | | | | | | 100.0 | | 100.0 | | 95.2 | | 95.5 | 95.2 | 100.0 |
| 12 | A | ACCGAGTACGGG | | | | | | | | 90.0 | 90.7 | | | | 90.0 | 90.7 |
| 12 | B | ACTCGGTCCCGT | | | | | | | | 94.0 | 89.8 | | | | 89.8 | 94.0 |
| 12 | a | AGCAGCCTATCC | | 91.4 | | | | | | | | | | | 91.4 | 91.4 |
| 15 | a | AAAAATAGTGGACCG | | | | | | | | | | 92.3 | | | 92.3 | 92.3 |
| 15 | a | AAAAGTCTACTGATT | | 100.0 | | | | | | | | | | | 100.0 | 100.0 |
| 15 | b | AACTTAGTTTATTTG | | | | | | | | | | 96.2 | 98.9 | | 96.2 | 98.9 |
| 15 | b | AACTTAGTTCATTTG | | | | | | | | | | 97.2 | | | 97.2 | 97.2 |
| 15 | b | ACATTTGCTCCGCCG | | | | | | | | | | 93.1 | | | 93.1 | 93.1 |
| 15 | a | AACCCAGCCTGACCC | | | | | | | | | | 100.0 | | | 100.0 | 100.0 |
| 15 | a | AAGTCACGGAGGACG | | | | | | | | | | 93.2 | | | 93.2 | 93.2 |
| 15 | b | ACGATGGTGTCGATG | | | | | | | | | | 94.0 | | | 94.0 | 94.0 |
| 16 | a | AAAAATCTCAAAGACC | | | | | | | | | | 92.7 | | | 92.7 | 92.7 |
| 16 | a | AAATTCGAAGGCATCC | | | | | | | | | | 93.1 | | | 93.1 | 93.1 |
| 17 | A | AAAAGTAGAACTTTTCT | | | | | 90.5 | | | | | | | | 90.5 | 90.5 |
| 17 | B | AAAAGTTCTACTTTTAG | | | | | 89.4 | | | | | | | | 89.4 | 89.4 |
| 17 | A | AAAAGTTCAACTTTATG | | | | | 93.2 | | | | | | | | 93.2 | 93.2 |
| 17 | B | AAAGTTGAACTTTTCAT | | | | | 94.3 | | | | | | | | 94.3 | 94.3 |
| 17 | b | AAAGTAGAACTTTTGTC | | | | | 89.6 | | | | | | | | 89.6 | 89.6 |
| 18 | a | AAAGAAGAAGAGAAGAGG | | | | | | | 88.7 | | | | | | 88.7 | 88.7 |
| 18 | a | AAATTTCAAGGAACGTCG | | | | | | | | | | 93.8 | | | 93.8 | 93.8 |
| 18 | b | ACGGTGCTGGACTCGGTT | | 100.0 | | | | | | | | | | | 100.0 | 100.0 |
| 18 | a | AACGTGAACGTGATCGTG | | | | | | | | | | 87.8 | | | 87.8 | 87.8 |
| 19 | a | AAGAAGTAAGAATAAGAGT | | | | | | | | | | 86.8 | | | 86.8 | 86.8 |
| 20 | b | ACGTCCATCGTGGTCCATCT | | | | | | | | | | 96.5 | | | 96.5 | 96.5 |
| 21 | a | AAAATGATGGAAATCAGGACG | | | | | | | | | | 97.2 | | | 97.2 | 97.2 |
| 21 | a | AATAATAATAATGATAATGAT | | | | | | | | | | 96.0 | | | 96.0 | 96.0 |
| 21 | a | AATAGTAGTAGTAGTAGTAGT | | | | | | | | | | 91.1 | | | 91.1 | 91.1 |
| 21 | b | ACTGGTCCCGTACTGGTCCGT | | | | | | | | | 86.9 | | | | 86.9 | 86.9 |
| 22 | b | AACTACTACATTTATTACTTTT | 96.0 | | | | | | | | | | | | 96.0 | 96.0 |
| 22 | a | AAAGAATCGTCGAAGAATCGTC | | | | | | | | | | 91.3 | | | 91.3 | 91.3 |
| 22 | a | ACCAGTACGGGACGAGTACGGG | | | | | | | | | 86.8 | | | | 86.8 | 86.8 |
| 23 | a | ACCAGTACGGGACCCAGTACGGG | | | | | | | | | 86.7 | | | | 86.7 | 86.7 |
| 23 | A | ACCAGTACGGGACCGAGTACGGG | | | | | | | | | 88.4 | | | | 88.4 | 88.4 |
| 23 | B | ACTCGGTCCCGTACTGGTCCCGT | | | | | | | | 89.8 | 90.3 | | | | 89.8 | 90.3 |
| 24 | b | AAATTTTAGTTATATTTGAAGTTC | | | | | | | | | | 90.3 | 91.7 | 91.7 | 90.3 | 91.7 |
| 24 | b | CGGTGTTGTCTTGTCTGTTGGCTT | | 93.5 | | | | | | | | | | | 93.5 | 93.5 |
| 24 | a | AAGGCAGCGGAGTACTACACCACC | | | 100.0 | 99.1 | 99.3 | | | | | | | | 99.1 | 100.0 |
| 24 | a | ACCGTCTGCTCCGCCGGCCCAGCC | | | 97.0 | 97.5 | 96.7 | | | | | | | | 96.7 | 97.5 |
| 26 | b | AAATTTCCGTGTGAAGAGGAATTGAT | | | | | | | | | | | 97.4 | 95.6 | 95.6 | 97.4 |
| 28 | a | AAATAACTTATCAATTCCTCCTCGGACG | | | | | | | | | | | 94.1 | | 94.1 | 94.1 |
| 30 | b | AAATACGTGCGTTTAGAAATTCCAGAAATC | | | | | | | | | | 98.9 | | | 98.9 | 98.9 |
| 30 | b | ATGATGATGATGATGATGATGATGATGATT | | | | | | | | | | 90.4 | | | 90.4 | 90.4 |
| 30 | b | AGTCCATCGTAGTCCATCTATGTCCATCGT | | | | | | | | | | 95.6 | | | 95.6 | 95.6 |
| 30 | a | ACATAGATGGACTACGATGGGCTACGATGG | | | | | | | | | | 89.9 | | | 89.9 | 89.9 |
| 31 | a | AAAAATAAATTATTTCGTTTGCCCACCCTTT | | | | | | | | 87.7 | 89.4 | | | | 87.7 | 89.4 |
| 31 | b | AAACGAAATAATTTAATTTTAAAGGGTGGGC | | | | | | | | 88.2 | 87.6 | | | | 87.6 | 88.2 |
| 31 | a | AAGTAGTAGTAGTAGTAGTAGTAGTAGTAGT | | | | | | | | | | 89.3 | | | 89.3 | 89.3 |
| 33 | b | ACTCGGTCCCGTACTGGTCCCGTACTGGTCCGT | | | | | | | | | 85.4 | | | | 85.4 | 85.4 |
| 34 | b | ATCGTTCCTGTGGCTTTATGCCGGCTTCGTGCCC | | | 95.3 | | 95.5 | | | | | | | | 95.3 | 95.5 |
| 34 | b | ACTCGGTCCCGTACTGGTCCCGTACTGGTCCCGT | | | | | | | | | 89.2 | | | | 89.2 | 89.2 |
| 35 | b | ACTCGGTCCCGTACTCGGTCCCGTACTGGTCCCGT | | | | | | | | 89.3 | 88.1 | | | | 88.1 | 89.3 |
| 36 | b | AAATTGAAGTTACAATTTTTGTTATATTTGAAGTTC | | | | | | | | | | 94.4 | | 92.1 | 92.1 | 94.4 |
| 36 | b | AAAATTTTTGTTATATTTGAAGTTCAAGTTGAAGTT | | | | | | | | | | 94.4 | | | 94.4 | 94.4 |
| 36 | b | AAAATTTTTGTTATATCTGAAGTTCAAATTGAAGTT | | | | | | | | | | 95.3 | 95.4 | 94.0 | 94.0 | 95.4 |
| 36 | b | AAAATTTTTGTTATATCTGAAGTTCAAATGGAAGTT | | | | | | | | | | 95.3 | | 91.9 | 91.9 | 95.3 |
| 36 | b | ACCTTCTCCGTCCTTTCTCTCCTCAGCTTCATCTTC | 98.6 | | | | | | | | | | | | 98.6 | 98.6 |
| 39 | a | AAACCAACTGAGGGAACGAGTGCCAAGCCAACAACTTTG | | | | | | | 88.8 | | | | | | 88.8 | 88.8 |
| 45 | b | AAATATTCCAAGTATCAAGATCGAAGTAAGTTTGAGTAGATTGAG | | | | | | | | | | 98.5 | | | 98.5 | 98.5 |
| 45 | a | AACGACGACAACGTCAACGACGACAACGTCAACGCCGACACCGAC | | | | | | | | | | 97.7 | | | 97.7 | 97.7 |
| 45 | a | ACCAGTACGGGACCAGTACGGGACCAGTACGGGACCGAGTACGGG | | | | | | | | | 92.0 | | | | 92.0 | 92.0 |
| 46 | a | ACCAGTACGGGACCAGTACGGGACCGAGTACGGGACCGAGTACGGG | | | | | | | | | 89.0 | | | | 89.0 | 89.0 |
| 47 | b | AACACTCTTCCTTCTCGGATGGATTTGAGCAGTTGGAACGTGAAGAG | | | 86.3 | | | | | | | | | | 86.3 | 86.3 |
| 47 | b | AACACTCTTCTCTTCCGGATGGATTTGAGCAGTTGGAACGTGAAGAG | | | 86.7 | | | | | | | | | | 86.7 | 86.7 |
| 48 | a | AAAAATGTCAAAGATTGTTGTATAAGGGATATAATTGATTATTGTAAG | | | | | | | | | | 97.0 | | | 97.0 | 97.0 |
| 48 | a | AACAACAACTTCCACAAGTGGCCCAACGACAACTACTCCTAGATCCAC | | | | | | | 96.6 | | | | | | 96.6 | 96.6 |
| 48 | a | ACCGAGTACGGGACCGAGTACGGGACCGAGTACGGGACCTAGTACGGG | | | | | | | | | 86.4 | | | | 86.4 | 86.4 |
